# American Society for Microbiology evidence-based laboratory medicine practice guidelines to reduce blood culture contamination rates: a systematic review and meta-analysis

**DOI:** 10.1128/cmr.00087-24

**Published:** 2024-11-04

**Authors:** Robert L. Sautter, James Scott Parrott, Irving Nachamkin, Christen Diel, Ryan J. Tom, April M. Bobenchik, Judith Young Bradford, Peter Gilligan, Diane C. Halstead, P. Rocco LaSala, A. Brian Mochon, Joel E. Mortensen, Lindsay Boyce, Vickie Baselski

**Affiliations:** 1 RL Sautter Consulting LLC, Lancaster, South Carolina, USA; 2 Department of Interdisciplinary Studies, Rutgers School of Health Professions, Newark, New Jersey, USA; 3 Department of Epidemiology, Rutgers School of Public Health, Newark, New Jersey, USA; 4 The State University of New Jersey, New Brunswick, New Jersey, USA; 5 Department of Public Health and Community Medicine, Tufts Medical School, Boston, Massachusetts, USA; 6 Department of Pathology and Laboratory Medicine, University of Pennsylvania Perelman School of Medicine, Philadelphia, Pennsylvania, USA; 7 Wellstar MCG Health, Augusta, Georgia and The State University of New Jersey, New Brunswick, New Jersey, USA; 8 Garnet Health Medical Center - Catskills, New York, Harris, New York, USA; 9 The State University of New Jersey, New Brunswick, New Jersey, USA; 10 Department of Pathology and Laboratory Medicine, Penn State Hershey Medical Center, Penn State College of Medicine, Hershey, Pennsylvania, USA; 11 College of Nursing and Health Sciences, Southeastern Louisiana University, Hammond, Louisiana, USA; 12 University of North Carolina School of Medicine, Chapel Hill, North Carolina, USA; 13 Global Infectious Disease Consultants, LLC, Jacksonville, Florida, USA; 14 Department of Pathology and Laboratory Medicine, University of Connecticut Health, Farmington, Connecticut, USA; 15 Department of Pathology, College of Medicine–Phoenix, University of Arizona, Phoenix, Arizona, USA; 16 Banner Health/Sonora Quest Laboratories, Phoenix, Arizona, USA; 17 Department of Pathology and Laboratory Medicine, Cincinnati Children’s Hospital Medical Center, Cincinnati, Ohio, USA; 18 Department of Research Informatics, MSK Library, Memorial Sloan Kettering Cancer Center, New York, New York, USA; 19 Department of Pathology and Laboratory Medicine, University of Tennessee Health Science Center, Memphis, Tennessee, USA; Rush University Medical Center, Chicago, Illinois, USA; National Institutes of Health, Bethesda, Maryland, USA

**Keywords:** blood cultures, blood culture contamination, bloodstream infections, bacteremia, practice guidelines, systematic reviews

## Abstract

Blood cultures (BCs) are one of the critical tests used to detect bloodstream infections. BC results are not 100% specific. Interpretation of BC results is often complicated by detecting microbial contamination rather than true infection. False positives due to blood culture contamination (BCC) vary from 1% to as high as >10% of all BC results. False-positive BC results may result in patients undergoing unnecessary antimicrobial treatments, increased healthcare costs, and delay in detecting the true cause of infection or other non-infectious illness. Previous guidelines from the Clinical and Laboratory Standards Institute, College of American Pathologists, and others, based on expert opinion and surveys, promoted a limit of ≤3% as acceptable for BCC rates. However, the data supporting such recommendations are controversial. A previous systematic review of BCC examined three practices for reducing BCC rates (venipuncture, phlebotomy teams, and pre-packaged kits). Subsequently, numerous studies on different practices including using diversion devices, disinfectants, and education/training to lower BCC have been published. The goal of the current guideline is to identify beneficial intervention strategies to reduce BCC rates, including devices, practices, and education/training by providers in collaboration with the laboratory. We performed a systematic review of the literature between 2017 and 2022 using numerous databases. Of the 11,319 unique records identified, 311 articles were sought for full-text review, of which 177 were reviewed; 126 of the full-text articles were excluded based on pre-defined inclusion and exclusion criteria. Data were extracted from a total of 49 articles included in the final analysis. An evidenced-based committee’s expert panel reviewed all the references as mentioned in Data Collection and determined if the articles met the inclusion criteria. Data from extractions were captured within an extraction template in the US Agency for Healthcare Research and Quality’s Systematic Review Data Repository (https://srdr.ahrq.gov/). BCC rates were captured as the number of events (contaminated samples) per arm (standard practice versus improvement practice). Modified versions of the National Heart, Lung, and Blood Institute Study Quality Assessment Tools were used for risk of bias assessment (https://www.nhlbi.nih.gov/health-topics/study-quality-assessment-tools). We used Grading of Recommendations, Assessment, Development and Evaluations to assess strength of evidence. There are several interventions that resulted in significant reduction in BCC rates: chlorhexidine as a disinfectant for skin preparation, using a diversion device prior to drawing BCs, using sterile technique practices, using a phlebotomy team to obtain BCs, and education/training programs. While there were no substantial differences between methods of decreasing BCC, our results indicate that the method of implementation can determine the success or failure of the intervention. Our evidence-based systematic review and meta-analysis support several interventions to effectively reduce BCC by approximately 40%–60%. However, devices alone without an education/training component and buy-in from key stakeholders to implement various interventions would not be as effective in reducing BCC rates.

##  INTRODUCTION

Blood cultures (BCs) are one of the most frequently used tests for detecting bloodstream infections, primarily in hospitalized patients. Bloodstream infections and associated sepsis have a high mortality rate with an estimated 200,000 deaths per year in the United States (1). Early treatment of significant bacteremia and sepsis is essential for good clinical outcomes (2). However, the interpretation of positive BCs by clinicians is complicated by not all positive BCs being clinically significant (3). Results from BCs may be falsely positive due to microbial contamination, with blood culture contamination (BCC) rates varying from 1% to higher than 10% (4). Many clinicians have difficulty in interpreting positive BCs when certain bacteria associated with normal skin microbiota are isolated (3). Controlling and lowering BCC rates is important to minimize the inappropriate use of antimicrobial agents, adverse events associated with antimicrobial use, unnecessary removal of lines, additional laboratory testing, increased length of stay, improper diagnosis, and controlling healthcare costs (4, 5). Medical institutions frequently use ≤3% BCC rates as a quality indicator determined by expert opinions and BCC rate surveys (6, 7) rather than evidence-based practice (8, 9), but lower rates may be achievable (4, 8, 10).

Doern and colleagues (4), in an expert opinion review, described specific elements for reducing BCC, focusing on specific changes that have been shown to reduce the contamination rate. However, little guidance is provided for how to successfully implement these changes within a laboratory or hospital system. In addition, there are a number of different approaches to defining BCC that make comparison of interventions to reduce BCC challenging (11). Implementation of individual elements is no guarantee of a meaningful reduction in BCC, and even a multicomponent intervention (tethering several desired practices) may founder for want of a systematic approach that fits within the particular context (12). Thus, the “how” of process improvement efforts is as important as the “what” if the effort is to ultimately reduce the level of contamination.

The purpose of this systematic review and meta-analysis is to update the 2012 BCC guideline published by the American Society for Microbiology (ASM) and the American Association for Clinical Chemistry (13) and to determine the effectiveness of different approaches (conditions of implementation success) to decreasing BCC rates (Box 1). The approach used in the current meta-analysis is different from the that previously used by ASM. The history of Laboratory Medicine Best Practices origins and its adaptation to Evidence-based Laboratory Medicine Practice Guidelines processes are described by Weissfeld and colleagues (14). Previous guidelines have also been published by the Emergency Nursing Association (15) and Centers for Disease Control and Prevention quality measure 3658, Adult Blood Culture Contamination Rate: A National Measure and Standard for clinical laboratories and antibiotic stewardship programs (https://p4qm.org/measures/3658).

Box 1. **Executive Summary**

Blood cultures are one of the most critical tests performed in clinical microbiology laboratories. Contamination of BCs with bacteria not associated with infection commonly occurs and is primarily due to inadequate procedures for collecting blood cultures. We examined several interventions for reducing BCC rates. While no single method was clearly superior, we recommend a series of interventions that were effective in reducing BCC that can be considered based on effectiveness and best fit with institutional needs.

1. Key action statement: Institutions (facilities) that draw BCs should consider incorporating chlorhexidine (with or without alcohol) into the protocol for skin antisepsis prior to drawing peripheral BCs in adult or pediatric populations (evidence quality: II, recommendation strength: moderate).

 Aggregate evidence quality: II

 Benefits: use of chlorhexidine skin antisepsis reduces BCC by an average of 57% which, in turn, may lead to more appropriate therapy for bloodstream infection.

 Risk, harm, and cost: use of chlorhexidine may be harmful in patients with sensitivity to chlorhexidine.

 Benefit–harm assessment: preponderance of benefit.

 Exclusions: patients with sensitivity to chlorhexidine.

2. Key action statement: institutions (facilities) that draw BCs should consider implementing a diversion device as part of the procedure for drawing peripheral BCs (evidence quality: II, recommendation strength: moderate).

 Aggregate evidence quality: II

 Benefits: diversion devices reduce BCC by an average of 64% and may lead to more appropriate therapy for bloodstream infections.

 Risk, harm, and cost: there is a potential to contribute to iatrogenic anemia in patients with prolonged hospital stays with frequent phlebotomy to obtain BCs if large amounts of blood are discarded. Diversion tubes must be labeled with patient information as with any other specimen tube to avoid unlabeled or mislabeled tubes being processed for other lab studies. The cost of using a non-commercial diversion tube should keep additional costs to a minimum.

 Benefit–harm assessment: preponderance of benefit.

3. Key action statement: clinical laboratory and institutional leadership should endorse having a specially trained team of phlebotomists (laboratory, nursing, and other medical professionals) perform peripheral venipunctures for obtaining BCs (evidence quality: II, recommendation strength: moderate).

 Aggregate evidence quality: II

 Benefits: blood cultures obtained by peripheral venipuncture and drawn by trained phlebotomists result in an average 41% decrease in BCC rates; less harm to patients (i.e., multiple sticks and bruising) when performed by trained phlebotomists and other medical professionals trained in blood-drawing techniques.

 Risk, harm, and cost: there is a significant cost to maintaining a trained phlebotomy team, but costs may be offset by fewer false-positive BC results.

 Benefit–harm assessment: preponderance of benefit.

4. Key action statement: institutions (facilities) should consider a standardized procedure for using sterile technique for drawing BCs by peripheral venipuncture (evidence quality: II, recommendation strength: moderate).

 Aggregate evidence quality: II

 Benefits: using a standardized sterile technique by all providers for obtaining BCs reduces BCC rates by an average of 56% and may lead to more appropriate therapy for bloodstream infections. Fewer false-positive BCs may reduce patient harm with inappropriate antimicrobial therapy and subsequent adverse events.

 Risk, harm, and cost: implementing a standard method for sterile collection of BCs may increase costs but may be offset by fewer false-positive BCs.

 Benefit–harm assessment: preponderance of benefit

5. Key action statement: clinical laboratories are responsible for mandating procedures for obtaining BCs and should work with institutional leaders to develop strong education programs (which include skills training and/or feedback and “continuous” monitoring) that may be integrated into larger quality management/quality assurance initiatives [quality management–quality improvement (QMQI)] for teams who draw BCs (laboratory phlebotomists, nurses, residents, and attendings) (evidence quality: I, recommendation strength: strong).

 Aggregate evidence quality: I

 Benefits: integrating intensive training programs into larger QMQI efforts to reduce BCC rates is demonstrated to bring about an average 56% reduction in BCC rates compared to only a 15% reduction when neither intensive training nor QMQI improvement efforts are used (*P* = 0.032).

 Analyses demonstrate that even using only one of these process improvement modalities (intensive training or integrating BCC reduction techniques into a larger QMQI effort) results in a 57% reduction in BCC rates.

 Risk, harm, and cost: the costs of implementing education programs or integrating BCC reduction techniques into a larger QMQI effort may be offset by reducing BCC rates and the adverse effects associated with false-positive BCs. Additionally, there is no known risk for a multicomponent approach to process improvement.

Benefit–harm assessment: preponderance of benefit.

**Fig 1:**
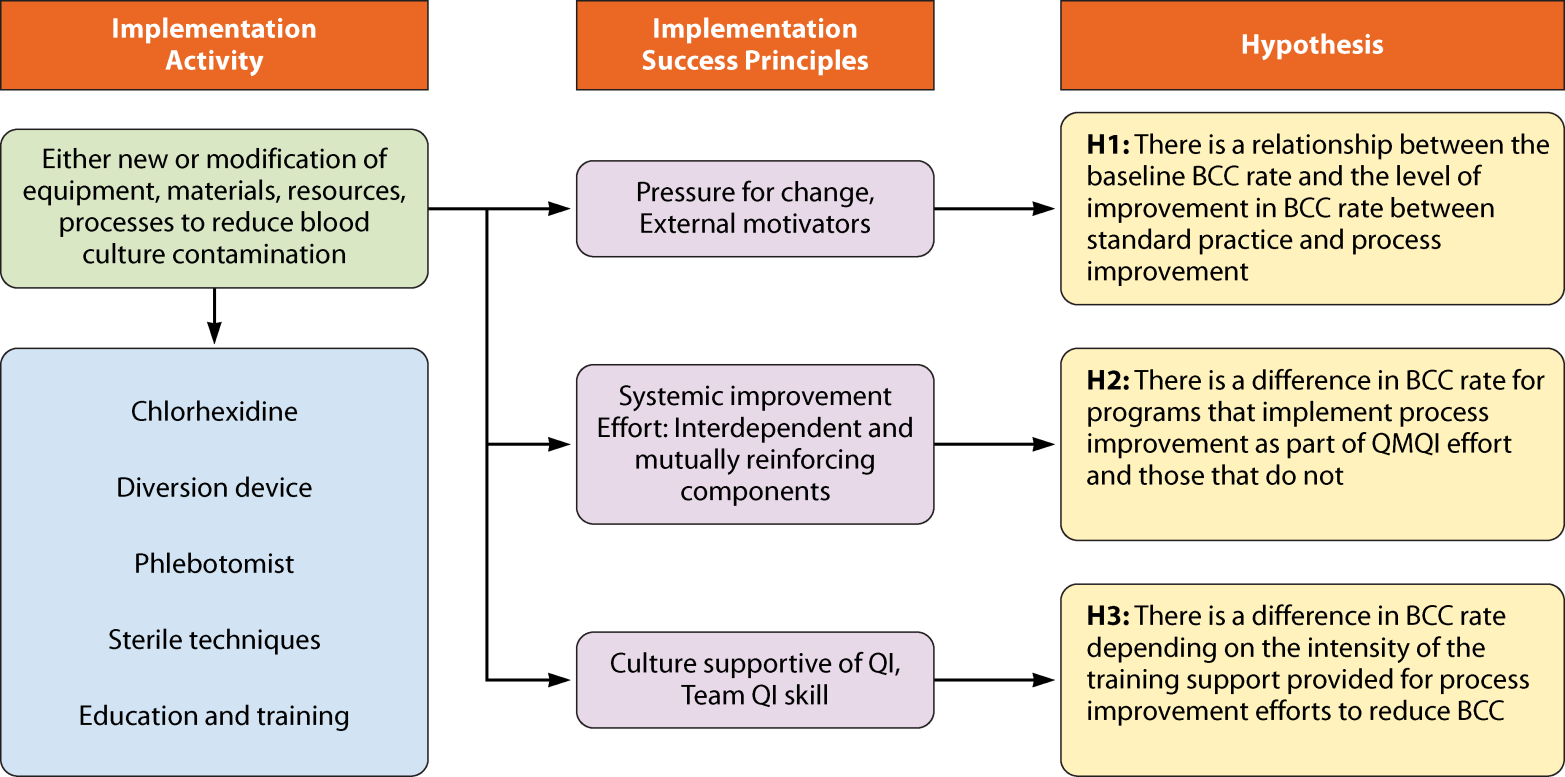
Conceptual framework for blood culture contamination analyses. QI, quality improvement.

Because process improvement interventions to decrease BCC rates are often multicomponent, our focus was not merely on particular technologies or equipment but also on the principles of implementation which are described below in Fig. 1 and reported in the implementation science literature to be successful. We drew from the Model for Understanding Success in Quality (MUSIQ) model of quality improvement (16) to generate our hypotheses. We hypothesized that, across different types of efforts to reduce BCC (i.e., different devices, different techniques, different communication strategies, and their combination), the following factors should be associated with greater improvement in reducing BCC: (i) baseline pressure for change (i.e., unacceptably high BCC rates); (ii) improvement plans that were systemic (i.e., were part of a larger quality improvement effort); and (iii) a culture conducive to learning and team skill (i.e., more intensive training efforts). The analytic approach is presented in Fig. 1.

Thus, in addition to evaluating the effectiveness of different discrete techniques for lowering BCC rates, our research question is, among institutions seeking to reduce BCC rates, are efforts consistent with implementation science principles (apart from the specific technologies, procedures, or communication strategies) effective in lowering contamination rates?

## METHODS

Details including search history, search strategy, record identification, and record screening

can be found in Supplemental File ASM-BCC Guideline Documentation and BCC Meta-Analysis.

### Information sources

The initial search was created and conducted by a medical librarian in October 2017 in eight databases: MEDLINE (Ovid), Embase (Ovid), Cochrane Central Register of Clinical Trials (Wiley), CINAHL (EBSCO), Scopus (Elsevier), EconLit (EBSCO), ClinicalTrials.gov, and the National Technical Information Service (ntis.gov). The updated searches were conducted by a medical librarian in February 2018 and December 2018 in a subset of the initial eight databases.

The final update in September 2022 was conducted by a second medical librarian (L.B.) and included the following six databases: MEDLINE (Ovid), Embase (Elsevier), Cochrane Central Register of Clinical Trials (Wiley), CINHAL (EBSCO), Scopus (Elsevier), and ClinicalTrials.gov. After the September 2022 update, retrieved systematic reviews were then hand searched for additional records.

### Search strategy

The search strategies consisted of keyword searches and, when applicable, controlled vocabulary related to BCs, phlebotomy, infection control, and contamination in each database using both Boolean operators and proximity searching to focus on the search results (see Supplemental File ASM-BCC Guideline Documentation 8.6.24)

### Selection process

Records were deduplicated, organized, and stored using the bibliographic management system EndNote and systematic review management system Covidence. Duplicates were identified using automated duplicate identification in EndNote and Covidence. Screening occurred at both the title and abstract stages, followed by the full-text stage concurrent with data extraction, using inclusion/exclusion criteria developed a priori.

**Fig 2:**
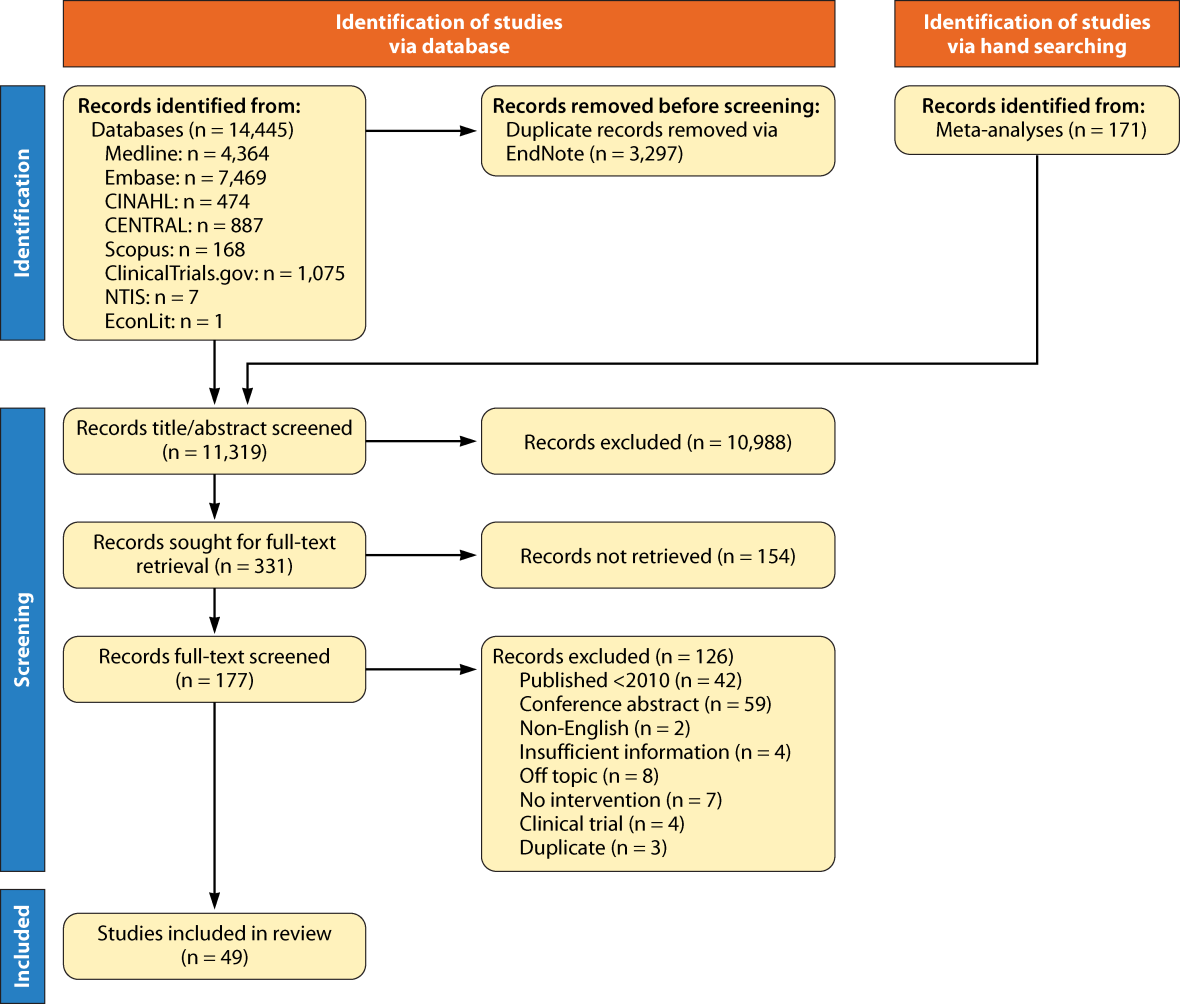
Preferred Reporting Items for Systematic Reviews and Meta-Analysis flow diagram for the systematic review process.

Across all the searches conducted, 14,616 total records were identified, of which there were 11,319 unique records following the automatic removal of duplicates. Of the 11,319 unique records identified, a total of 331 were sought for full-text screening, and 177 were ultimately retrieved and included (154 were not full-text studies, absent data, or abstracts). Through full-text review, an additional 126 records were excluded based on pre-defined inclusion and exclusion criteria. Data were extracted from a total of *n* = 49 articles (incorporating data on *n* = 53 comparisons) included in the final analysis (Fig. 2).

Following the references found on the subject, the evidence-based committee’s expert panel reviewed all the references as mentioned in Data Collection and determined if the articles were of substantial data that were unbiased and detailed interventions that were useful to possibly achieve BCC goals.

### Data collection

Data from extractions were captured within an extraction template in the US Agency for Healthcare Research and Quality’s Systematic Review Data Repository (https://srdr.ahrq.gov/). The extraction template was created and pilot tested (17) for this project. All sources were double blind extracted by two analysts, and conflicts were resolved by a third team member.

### Data items

In addition to arm and outcome information, data were also collected on study design details (e.g., study design, sampling strategy, funding source, and setting), arm details (examining the combination of implementing equipment/materials/resource changes or modifications along with the intensity of staff training efforts), sample characteristics (organisms identified), results of the outcomes of interest (BCC rate), and risk of bias (ROB).

### Study risk of bias assessment

Modified versions of the National Heart, Lung, and Blood Institute Study Quality Assessment Tools were used for ROB assessment (https://www.nhlbi.nih.gov/health-topics/study-quality-assessment-tools). Question wording and instructions included in the three ROB tools relevant for this project (before–after, cohort, and controlled intervention) were tailored to fit the lab improvement process context. To determine the overall ROB for each study, questions were organized into three domains (patient selection, intervention related, and outcome related), and critical flaw criteria relevant to lab improvement processes were determined by the team. An assessment algorithm was developed and then applied to each study. The study team then reviewed individual study ROB ratings and reached consensus on all studies. All studies were double blind evaluated for ROB with a third team member adjudicating any disagreement.

### Strength of evidence

We adapted the Grading of Recommendations, Assessment, Development and Evaluations (GRADE) method for evaluating the strength of the evidence (18, 19) (see Table S1). The purpose of GRADE is to help researchers creating systematic reviews of interventions or clinicians seeking to answer a clinical question to evaluate the overall strength of the evidence (i.e., across all studies included) (20, 21). While GRADE was designed for use with treatment questions, it has been adapted for use with diagnostic accuracy questions (21). GRADE has not, however, been fully adapted for use in an organizational process improvement context (22).

While the GRADE approach assumes an evidence hierarchy (with randomized controlled trials as the primary design for determining causality), implementation science scholars have suggested that an evidence typology (where the preferred design is tailored to the nature of the question) is more appropriate within the context of process improvement studies (22). Indeed, principles for the reduction bias in treatment studies (e.g., random allocation and blinding) may be at direct odds with principles demonstrated to be effective in implementation studies (a shared understanding of the need for improvement, teams and leaders committed to continued monitoring of change, coordinated effort and accountability to bring about change, etc.) (23, 24). For example, while a before–after study design that uses census sampling of administrative data may be less trustworthy for determining the effectiveness of a new drug than a randomized controlled trial (RCT), it may be the ideal study design to evaluate the effectiveness of an organization or system’s improvement efforts. While the goal of an RCT is to identify a causal relationship decontextualized from variations in patient sample, organizational context, etc., process improvement efforts are highly embedded within particular organizations with particular stakeholders and are thus heavily context dependent (25). Thus, when evaluating the strength of evidence of our findings, we use GRADE criteria but adjust the baseline “level of evidence” to fit the context of lab process improvement.

### Statistical methods

#### Effect measures

Blood culture contamination rates were captured as the number of events (contaminated samples) per arm (standard practice versus improvement practice). These values were used to compute risk ratios (RRs). Risk ratios (rather than odds ratios) were used to express the effect size since for rare events they are very similar and because the large proportion of the studies included used census sampling, where an accurate measure of the at-risk population could be determined (26). In some studies, the intervention effort was implemented in stages (e.g., standard process → improvement stage 1 → improvement stage 2), and so outcomes were reported at multiple times. For this analysis, only the last, most comprehensive stage of the implementation was used for comparison to baseline.

#### Synthesis eligibility, missing data, and data estimation

To be included in the study, contamination rates (described above) had to be reported or able to be estimated. Where contamination rates were reported as a standardized rate (e.g., *X*/1,000 patients), the typical census rate (when reported) was used to estimate the expected number of contamination events per improvement period. For multicenter studies that reported outcomes separately for the different centers (27, 28), data were captured and analyzed on a by-center basis if the interventions differed across centers.

#### Statistical methods

For all models, random effects meta-analyses were used. For binary outcomes, the Paule–Mandel procedure was used to estimate *τ*^2^ due to its good performance across analysis scenarios (29). Binary outcomes risk ratios were computed. R packages meta, metafor and demetar were used for the analyses. Outcomes are reported as risk ratios. Meta-regression was used to examine the effect of continuous moderators.

While most interventions were multicomponent, for articles that focused on discrete techniques or approaches for reducing BCC rates, we were able to examine the effectiveness of the following interventions:

- Use of chlorhexidine (with and without alcohol) versus standard practice (*n* = 10 studies).
- Use of a diversion device versus standard practice (*n* = 6 studies).
- Use of sterile techniques versus standard practice (*n* = 6 studies).
- Use of a dedicated phlebotomy team versus standard practice (*n* = 2 studies).
- Education/training as the only intervention versus standard practice (*n* = 16 studies).

The general effect of any process improvement was evaluated by analyzing data from all included studies. We then tested the three hypotheses (Fig. 1):

Hypothesis 1: There is a relationship between the baseline BCC rate and the level of improvement in BCC rate between standard practice and process improvement.

Hypothesis 2: There is a difference in BCC rate for programs that implement process improvement as part of a quality management–quality improvement (QMQI) effort and those that do not.

Hypothesis 3: There is a difference in BCC rate, depending on the intensity of the training support provided for process improvement efforts to reduce BCC.

In addition to testing each of these hypotheses separately, we additionally tested whether combining intensive training with a QMQI (combined hypotheses 2 and 3) resulted in different results compared to either process improvement strategy alone and compared to interventions where neither process improvement strategy was used. This allowed us to differentiate between efforts that used both, either, or none of the process improvement strategies.

#### Diagnostics and reporting bias

Publication bias was assessed via funnel plots and using Peters et al.’s regression (30). Outliers and influential cases were screened using the R {dmetar} package. The results of the sensitivity analyses based on outlier and influential cases (as well as the operational definition for outliers and influence) are available in the supplemental material (BCC Meta-Analyses). Publication bias was assessed across hospital unit type.

#### Heterogeneity and sensitivity analysis

Heterogeneity is reported using *I*^2^ (31). Analyses were carried out for all studies as well as broken out by units: emergency department (ED), general units, intensive care unit (ICU)/heme/oncology units, pediatric units, and combined units (i.e., results reported only across multiple different units).

We carried out a sensitivity analysis dropping formal outliers. Because of the broad definition of what could count as “process improvements to improve BCC rate,” we anticipated a wide variation in the structure of different efforts, made even more variable by the heterogeneity of the processes and structures in place prior to the process. Thus, by carrying out a sensitivity analysis dropping the formal outliers, this provides a sense of the effects that most “average” improvement efforts could expect. We define outliers as cases (i) for which the upper bound of the 95% confidence interval (CI) is lower than the lower bound of the pooled effect confidence interval (i.e., extremely small effects) and (ii) for which the lower bound of the 95% CI is higher than the upper bound of the pooled effect confidence interval (i.e., extremely large effects) (32).

Outlying studies are reported by outcome, and more detailed examination of “overperforming” and “underperforming” intervention efforts are provided.

## DESCRIPTION OF STUDIES

### Included studies

Forty-nine published articles met the inclusion criteria for the meta-analysis. This comprised 65 arms (e.g., outcomes reported separately for different units), *n* = 968,796 observations (samples) with *n* = 18,880 contamination events.

### Excluded studies

These are studies that did not include interventions to reduce BCC rates. Seven studies were excluded after the initial analysis and after additional review due to a lack of interventions reported (peripheral versus catheter-drawn BCs) or unrelated interventions to reduce contamination of peripherally drawn BCs.

### Risk of bias in included studies

Summary ROB plots for the three different classes of study designs used in this analysis are presented below. ROB criteria for individual studies are presented in the methodological supplement.

#### Before–after designs

**Fig 3:**
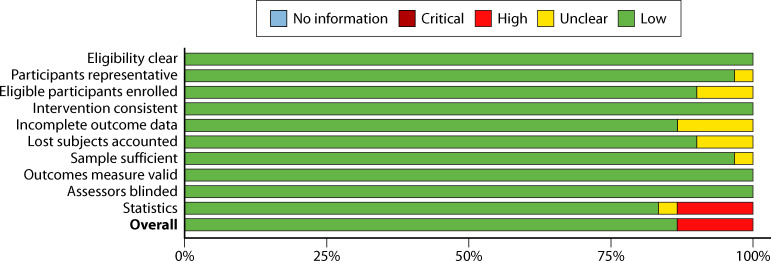
Summary risk of bias: before–after study designs.

Before–after designs were the most common study design used (63.3% of included studies) (Fig. 3). Typically, these studies relied on administrative data and utilized census sampling. The preference for this type of design is unsurprising, given the nature of clinic, hospital, or system improvement efforts where expense and training can be extensive (e.g., the cost to purchase new devices and time to train clinicians across the organization). The use of administratively collected data and census sampling (i.e., all patients or samples within a particular time frame) decreased bias. The primary risk identified for studies with this design was statistical; specifically, approximately 10% of these studies reported raw BCC rates without attempting to test for the statistical significance between the standard and process improvement rates.

#### Cohort design

**Fig 4:**
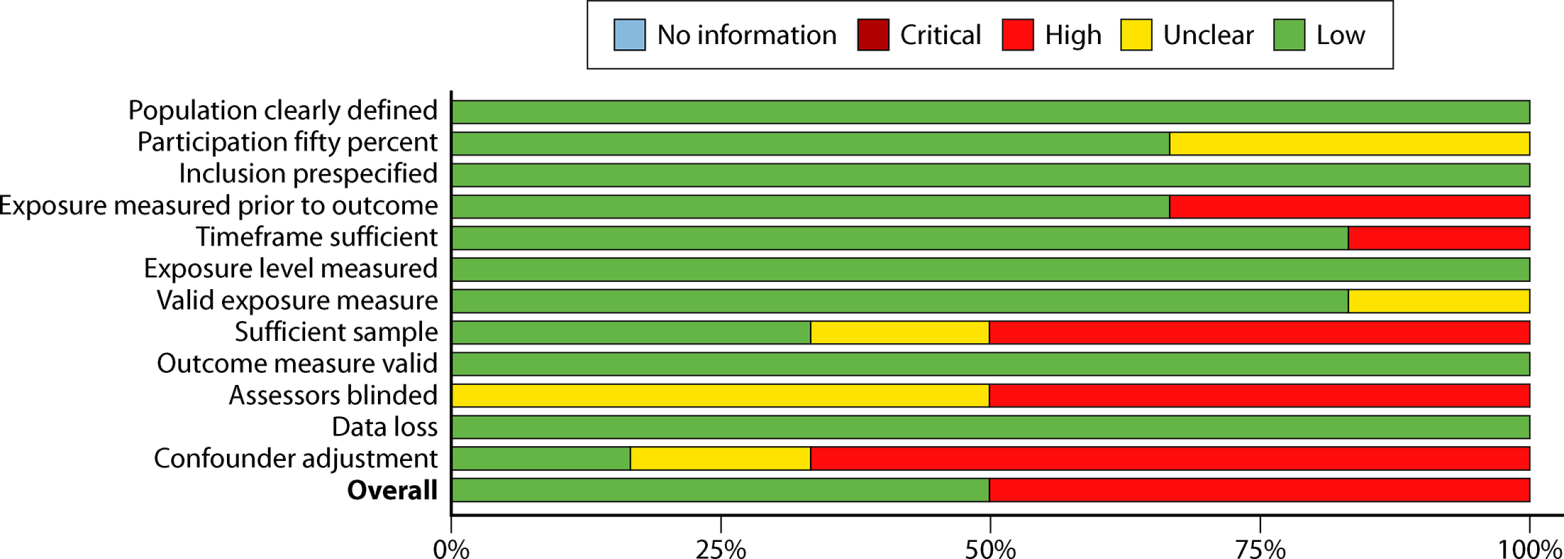
Summary risk of bias: cohort study designs.

Six studies (12.2%) utilized a cohort design (Fig. 4). These studies had a much larger proportion (50% weighted) of high-risk studies. Three criteria were of most concern. The most serious was lack of adjustment for confounders. Unlike the before–after designs, which followed changes in BCC rates for organization-wide process improvement efforts, cohort designs typically examined performance in contemporary units (which could have very different characteristics, utilize different personnel for blood collection, etc.). These characteristics would confound the differences in BCC rates. Also, because cohorts were defined based on a subsample of available units, there was often (>50%) question among evaluators whether the sample was sufficient for the analyses. This is in contrast to before–after designs where census sampling was typically used. Finally, blinding of the assessors was deemed to be a *greater* risk for this type of design since interventionists could likely be aware of competing practices and so (in the standard care arm) alter their usual practices, thus possibly biasing the comparison.

#### Controlled interventions

**Fig 5:**
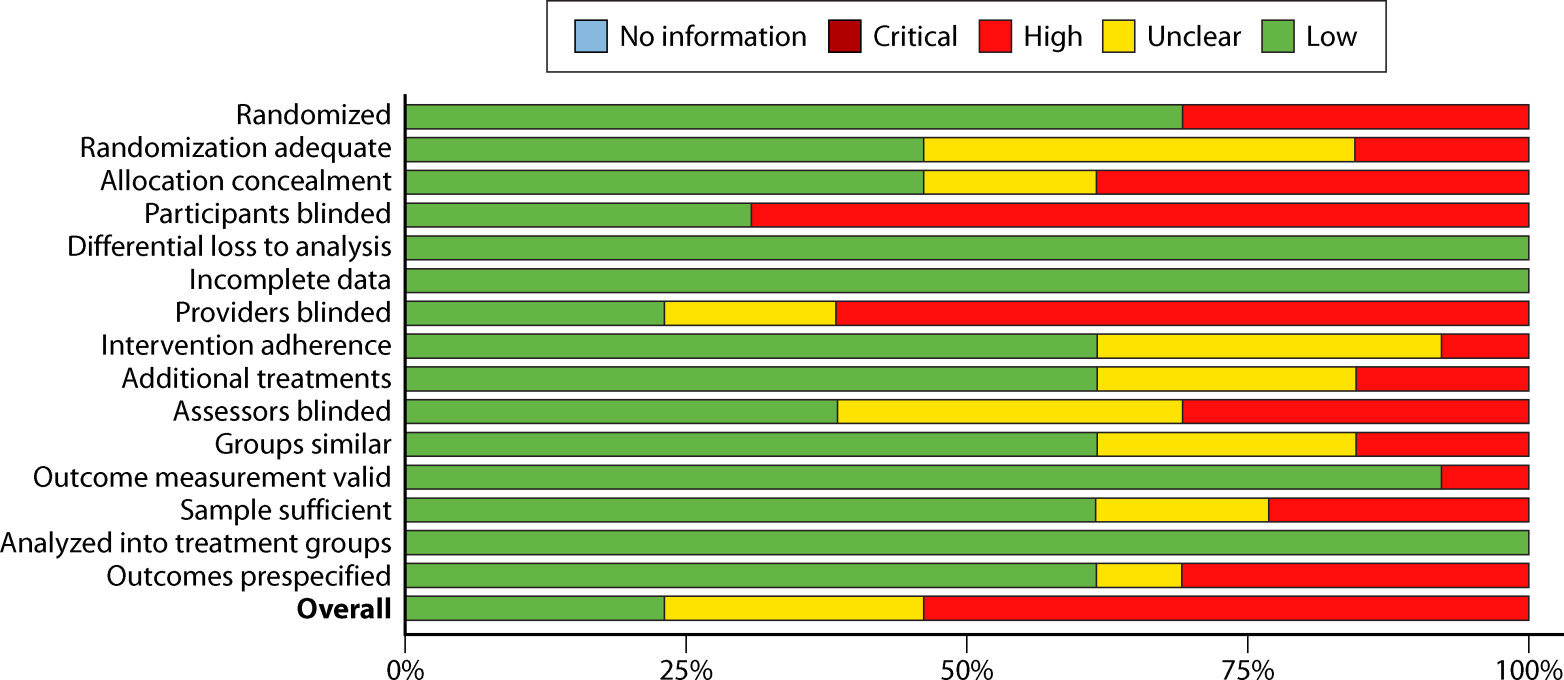
Summary risk of bias: controlled study designs.

Fourteen (26.5%) of the studies used a controlled design (Fig. 5). Although 71% of these studies described the study as randomized, the quality of the randomization and allocation concealment were a significant concern for over half the studies—largely due to a lack of description of how these procedures were carried out. Lack of blinding was also a major concern. While patient blinding is likely of little concern, provider blinding (in this context, very difficult to achieve) could lead to alteration of behaviors among providers in the comparison condition (e.g., more careful skin disinfection than would otherwise happen without knowledge of the intervention). As a result of these problems, fewer than 25% of the studies with this design were rated as overall low ROB.

Indeed, it may be that RCTs—the gold standard for typical treatment questions—may be generally unsuited for what often amounts to an institutional process improvement effort. Rather than conceive of these process improvement efforts as “research studies,” it may be more appropriate to consider them from an implementation science perspective, where factors such as stakeholder buy-in (clear lack of blinding) and performance incentives are known to be key characteristics of successful implementation efforts (33). Recent work in the area of implementation science recommends that evidence be conceived as a typology rather than a hierarchy (as with GRADE), such that “The choice and strength of study design is dependent on the research questions and setting, particularly the context for the study” (22). From this perspective, the higher risk evidence from controlled trials would be of less importance than the lower risk before–after implementation designs.

## RECOMMENDATIONS

Forty-nine publications encompassing 42 discrete interventions were included in our analysis, and results from five interventions were analyzed and presented in Table 1.

**TABLE 1:** Interventions examined in this study for reducing BCC rates^*a*^

Comparison	*n* studies	*n* observations	RR	RR LCL	RR UCL	*I*^2^ (%)	*P* value
Chlorhexidine	10	35,744	0.435	0.233	0.814	80.3	0.009
Diversion device	6	76,015	0.361	0.215	0.605	92.0	<0.001
Sterile technique	6	56,126	0.443	0.365	0.539	41.4	<0.001
Phlebotomist team	2	162,770	0.592	0.501	0.699	0	<0.001
Education/training	16	481,726	0.480	0.365	0.631	92.6	<0.001

^*a*^ *I*^2^, heterogeneity; RR, relative risk; RR LCL, relative risk lower confidence limit; RR UCL, relative risk upper confidence limit.

### Chlorhexidine

Question 1. Does using chlorhexidine (chlorhexidine gluconate with or without alcohol) as a skin preparation disinfection reduce blood culture contamination rates?

#### Recommendation

Institutions (facilities) that draw BCs should incorporate chlorhexidine (with or without alcohol) into the protocol for skin antisepsis prior to drawing peripheral BCs in adult or pediatric populations.

#### Literature review summary

There were 10 studies (10 interventions) that compared chlorhexidine as a skin disinfectant with other disinfectants such as tincture of iodine, povidone–iodine, or isopropyl alcohol (Table 2). Five studies used alcoholic chlorhexidine, and five used chlorhexidine alone. Five studies were in adult populations, and five studies involved pediatric populations. A variety of study designs were used, before–after designs (34–37), randomized controlled trials (38–40), cohort designs (41, 42), and non-randomized controlled design (43).

**TABLE 2:** Summary of studies on effectiveness of chlorhexidine for skin antisepsis to reduce BCC rates^*a*^

Study (reference)	Design	A or P	Control group	Intervention group	Pre-BCC rate/control group (%)	Post-BCC rate (%)
Kai et al. (34)	Before–after	A	10% povidone–iodine	1% CHG–alcohol	6.3	1.1
O’Connor et al. (36)	Before–after	P	70% isopropyl alcohol	2% CHG/70% isopropyl alcohol	3.8	0.96
Ryan (37)	Before–after	A	Alcohol preparation pads	CHG swabs	4.5	1.9
Marlowe et al. (35)	Before–after	P	10% povidone–iodine	3% CHG	2.5	1.7
Nuntnarumit and Sangsuksawang (38)	Randomized control	P	10% povidone–iodine	1% aqueous CHG	2.9	0
Story-Roller and Weinstein (40)	Randomized control	A	2% iodine tincture	2% CHG	3.93	3.88
Martinez et al. (39)	Randomized control	A	70% isopropyl alcohol	2% CHG/70% isopropyl alcohol	0.9	1.9
Maeda et al. (41)	Cohort design	P	10% povidone–iodine	0.5% wt/vol CHG ethanol	2.5	0.4
Tangsathapompong et al. (42)	Cohort design	P	70% isopropyl alcohol	2% CHG/70% isopropyl alcohol	3.21	2.28
Ge et al. (43)c	Non-randomized controlled design	A	0.45% chlorhexidine acetate plus 0.2% iodine	2.5% tincture of iodine plus 70% alcohol	1.25	0.16

^*a*^ A, adult; CHG, chlorhexidine gluconate; P, pediatric.

Overall, using chlorhexidine as a skin disinfection was associated with a BCC rate reduction of 56.5% (19%–77%) across all studies. Limitations to various studies included poor or limited definitions of BCC (38), different formulations of chlorhexidine (43), and multiple elements of interventions (34).

#### Evidence summary

**Fig 6:**
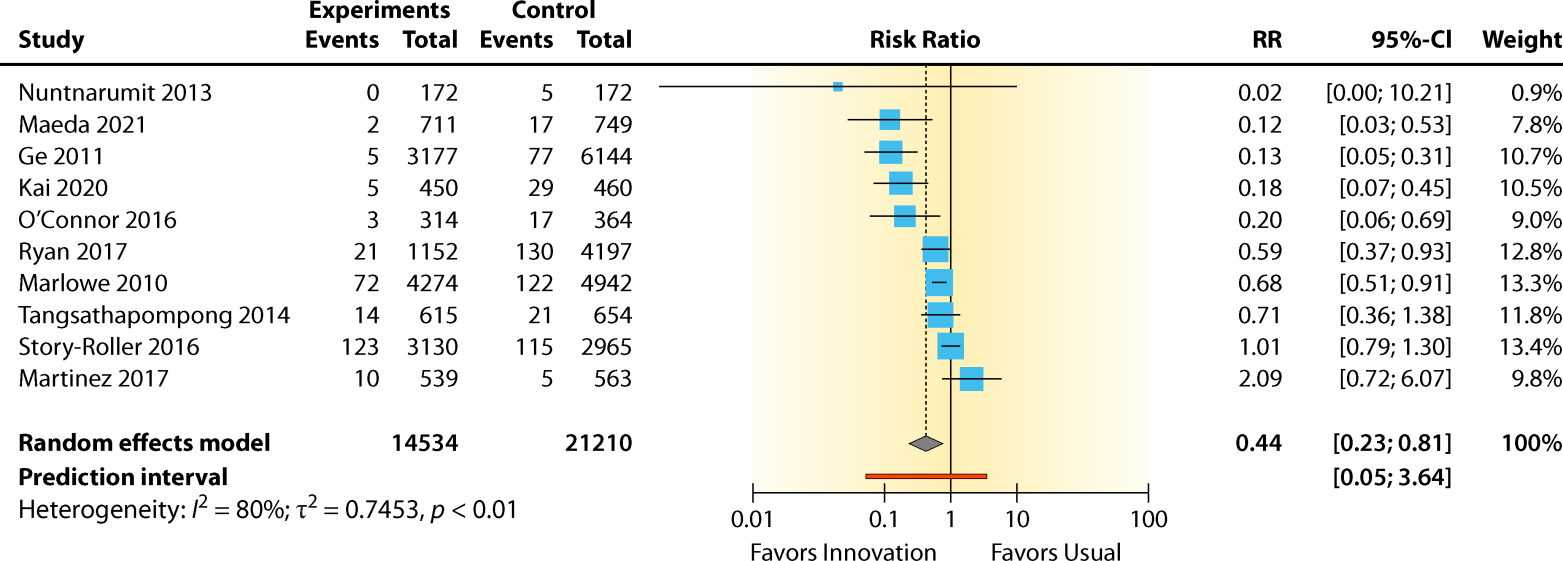
Forest plot of included studies with chlorhexidine as a skin antiseptic. References are as follows: Nuntnarumit and Sangsuksawang (38), Maeda et al. (41), Ge et al. (43), Kai et al. (34), O’Connor et al. (36), Ryan (37), Marlowe et al. (35), Tangsathapompong et al. (42), Story-Roller and Weinstein (40), and Martinez et al. (39).

The summary for the RR for chlorhexidine versus standard practice was 0.44 (95% CI 0.23–0.81, *P* = 0.009) for the BCC rate. A high heterogeneity effect was observed: *I*^2^ = 80.3% (95% CI 64.7–89.1%). The RR effect estimates favored chlorhexidine over standard practice for reducing contamination (Fig. 6). The overall contamination reduction was 56.5%. The intervention showed a significant benefit in lowering contamination (*P* = 0.009). However, this analysis has a substantial between-study heterogeneity, not including sampling error (*I*^2^ = 80.3%, *P* < 0.0001).

**Fig 7:**
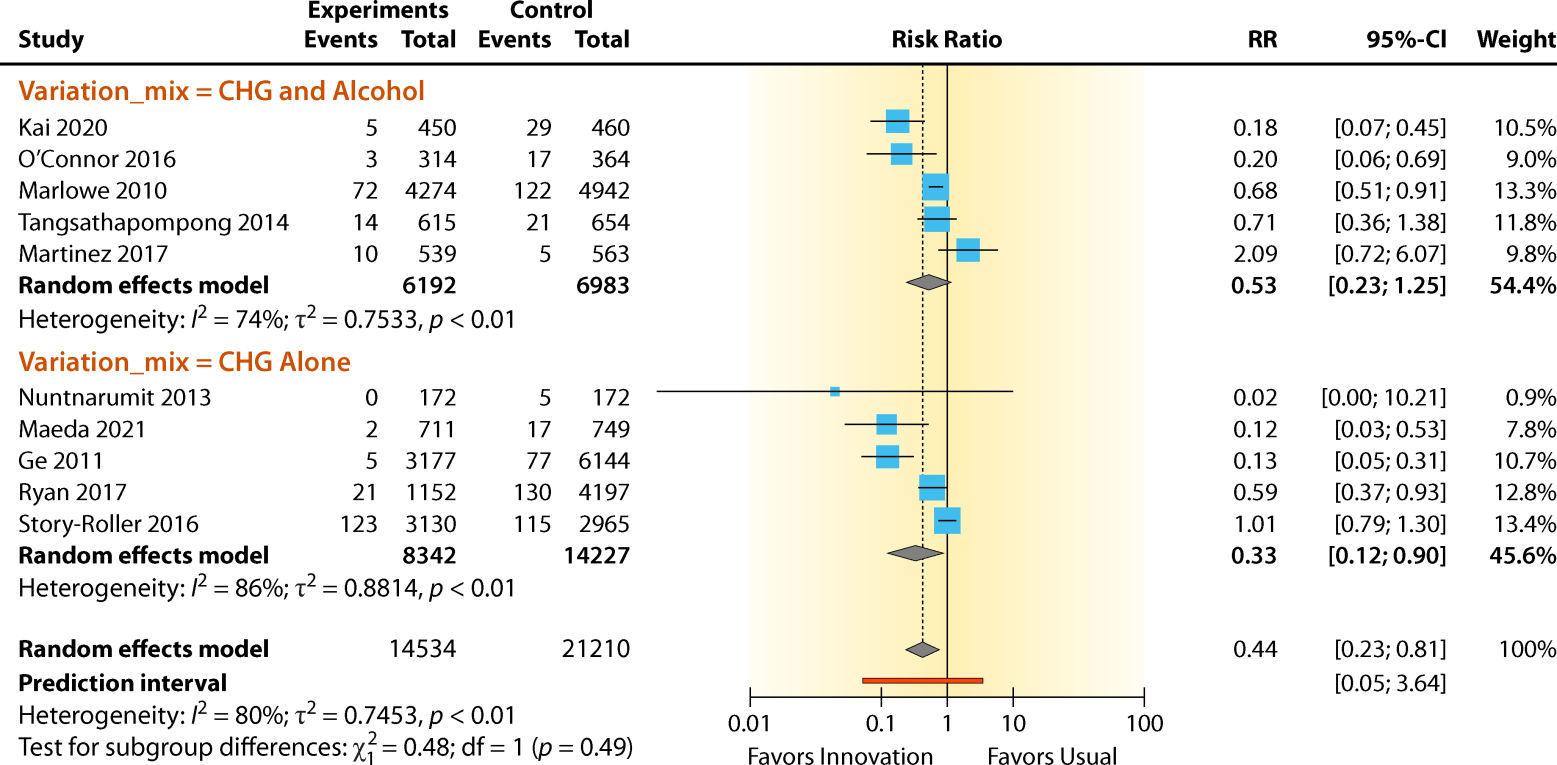
Forest plot of studies comparing chlorhexidine with other skin antiseptics, chlorhexidine alone, or chlorhexidine/alcohol. References are as follows: Kai et al. (34), O’Connor et al. (36), Marlowe et al. (35), Tangsathapompong et al. (42), Martinez et al. (39), Nuntnarumit and Sangsuksawang (38), Maeda et al. (41), Ge et al.(43), Ryan (37), and Story-Roller and Weinstein (40).

The summary for the RR for chlorhexidine gluconate (CHG) using CHG alone versus CHG with alcohol was RR = 0.33 (95% CI 0.12–0.90) and RR = 0.53 (95% CI 0.23–1.25) for the BCC rate, respectively (Fig. 7). High and moderate heterogeneity effects were observed for both CHG and CHG with alcohol, and they were *I*^2^ = 86% and 74%, respectively. The RR effect estimates favored chlorhexidine intervention in both groups over standard practice for reducing contamination. The overall contamination reduction for CHG was 67%, and that for CHG with alcohol was 47%. The heterogeneity for both groups were significant (*P* < 0.01). The differences between the two groups were insignificant (*P* = 0.488).

**Fig 8:**
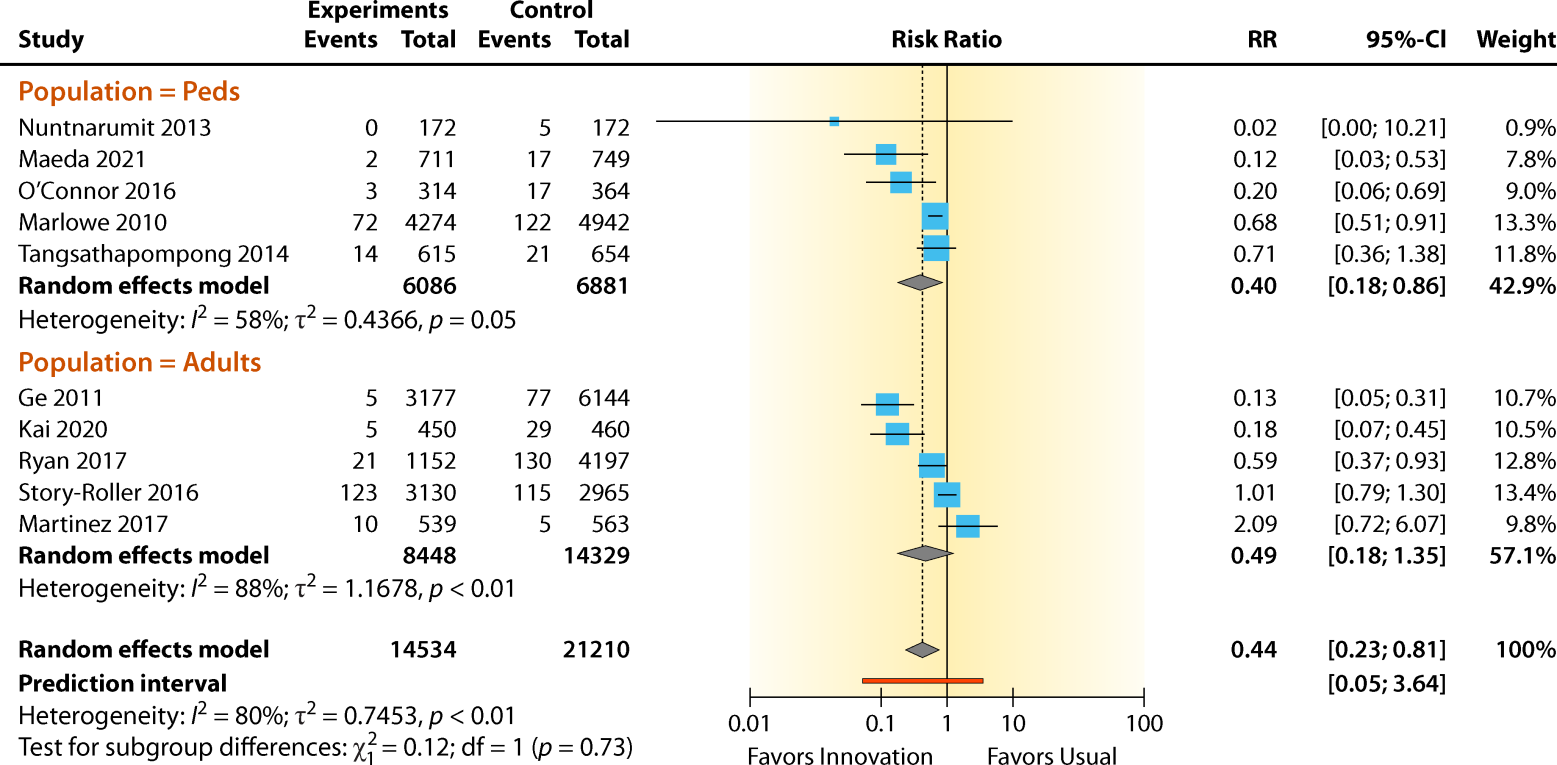
Forest plot of studies comparing chlorhexidine skin antisepsis in adult or pediatric populations. References are as follows: Nuntnarumit and Sangsuksawang (38), Maeda et al. (41), O’Connor et al. (36), Marlowe et al. (35), Tangsathapompong et al. (42), Ge et al. (43), Kai et al,. (34), Ryan (37), Story-Roller and Weinstein (40), and Martinez et al. (39).

The summary for the RRs for chlorhexidine intervention in the adult population versus the same intervention in the pediatric population was RR = 0.49 (95% CI 0.18–1.35) and RR = 0.40 (95% CI 0.18–0.86) for the BCC rate, respectively (Fig. 8). High and moderate heterogeneity effects were observed for the adult and pediatric populations with the chlorhexidine intervention and were *I*^2^ = 88% and 58%, respectively. The RR effect estimates favored chlorhexidine intervention in both groups over standard practice for reducing contamination. The overall contamination reduction for the adult population was 51% and that for the pediatric population was 60%. The heterogeneity for both groups was significant for the adult population (*P* < 0.01) but insignificant for the pediatric population (*P* > 0.05). The differences between the two groups were insignificant (*P* = 0.730).

### Diversion devices

Question 2. Does using a diversion device as part of the blood culture collection procedure reduce blood culture contamination rates?

#### Recommendation

Institutions (facilities) that draw BCs should implement using a diversion device as part of the procedure for drawing peripheral BCs.

#### Literature review summary

There were six studies that were compared using a diversion device, which removes a small sample of blood for discard before filling BC bottles, as part of the BC collection protocol, for reducing BCC rates (Table 3). Two studies used before–after designs (44, 45); three used randomized controlled trials (46–48); and one was a non-randomized controlled trial (49). Four studies used sterile discard tubes (non- or sterile Vacutainer tubes, sterile lithium heparin tubes, and gold/green top tubes) as the diversion device (44, 46–48), and two studies used a commercial diversion device kit (45, 49). Overall, using a diversion device resulted in 64% BCC rate reduction (40%–79%) across included studies.

**TABLE 3:** Summary of studies using diversion devices to reduce BCC rates^*a*^

Study (reference)	Design	A or P	Control	Diversion device (amount of blood used)	Pre-BCC rate (%)	Post-BCC rate (%)
Syed et al. (44)	Before–after	A	Skin prep immediately followed by BC collection	7-mL gold- or green-top tube collected before BC (7 mL)	2.46	1.7
Bell et al. (45)	Before–after	A	Skin prep immediately followed by BC collection	Steripath commercial device (1–2 ml)	3.52	0.6
Lalezari et al. (46)	Randomized control	A	Usual draw, 70% alcohol skin prep	Na heparin tube (N.S.)	5.0	1.7
Zimmerman et al. (48)	Randomized control	A	BC collection followed by lithium heparin tube	Lithium heparin tube followed by BC (N.S.)	5.0	2.0
Patton and Schmitt (47)	Randomized control	A	Skin prep immediately followed by BC collection	1 mL in a 3-mL Vacutainer collection tube (1 mL)	2.8	1.4
Rupp et al. (49)	Non-randomized control	A	Skin prep immediately followed by BC collection	Steripath commercial device (1.5–2.0 mL)	1.78	0.22

^*a*^ A, adult; N.S., not specified; P, pediatric.

#### Evidence summary

**Fig 9:**
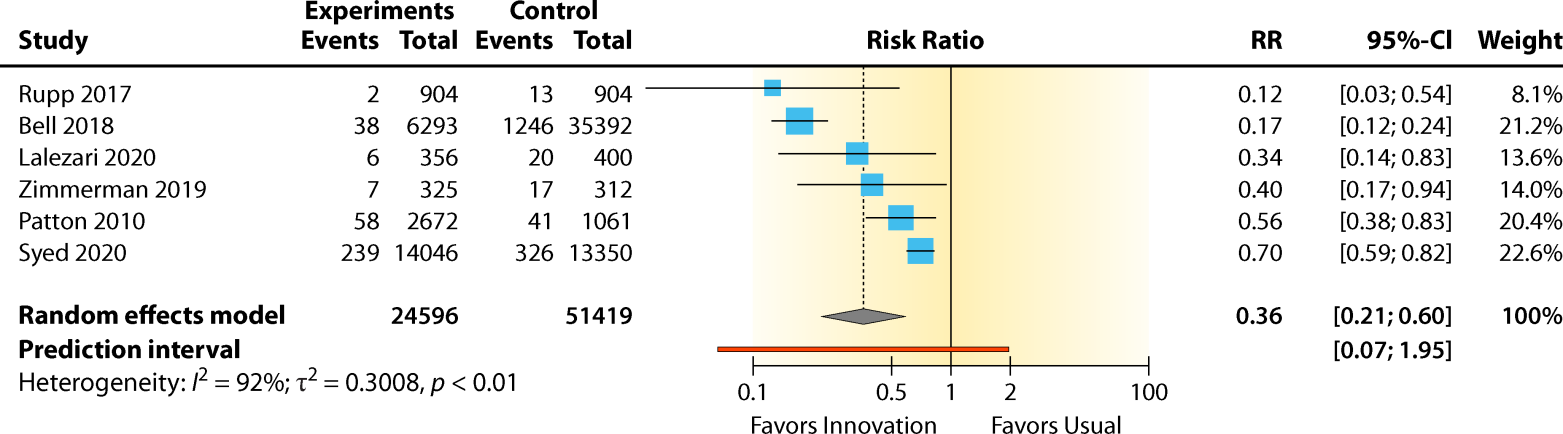
Forest plot of studies examining diversion devices for reducing BCC rates. References are as follows: Rupp et al. (49), Bell et al. (45), Lalezari et al. (46), Zimmerman et al. (48), Patton and Schmitt (47), and Syed et al. (44).

The summary for the RR for diversion device versus standard practice was 0.36 (95% CI 0.21–0.60, *P* < 0.01) for the BCC rate. A high heterogeneity effect was observed: *I*^2^ = 92.0% (95% CI 85.4%–95.6%). The RR effect estimates favored diversion device over standard practice for reducing contamination (Fig. 9). The overall contamination reduction was 64%. The intervention showed a significant benefit in lowering contamination (*P* < 0.01). However, this analysis has a substantial between-study heterogeneity, not including sampling error (*I*^2^ = 92.0%, *P* < 0.01).

### Phlebotomy teams

Question 3. Do trained phlebotomists have a lower BCC rate than other providers who draw blood cultures?

#### Recommendation

Clinical laboratory and institutional leadership should endorse having a specially trained team of phlebotomists perform peripheral venipunctures for obtaining BCs.

#### Literature review summary

Two studies (50, 51) used before–after study designs (Table 4). In addition to reducing BCC rates, phlebotomist teams also increased the volume of blood drawn in BCs from 2.1 ± 0.7 mL (SD) in the control group to 5.6 ± 1.3 mL (SD) with the phlebotomy team (50).

**TABLE 4:** Summary of studies using phlebotomy teams for reducing BCC rates

Study (reference)	Design	A or P^*c*^	Control	Intervention	Pre-BCC rate	Post-BCC rate
Santos et al. (51)	Before–after	A	Nursing, physicians	Trained phlebotomists	1.3/1,000	0.8/1,000^*a*^
Bae et al. (50)	Before–after	A	MD interns	Trained phlebotomists	0.45^*b*^	0.27

^*a*^ Rate/1,000 patient days.

^*b*^ Rate (%).

^*c*^ A, adult; MD, medical doctor; P, pediatric.

#### Evidence summary

**Fig 10:**
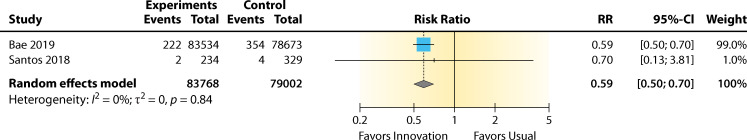
Forest plot of studies on phlebotomy teams and effect on reducing BCC rates. References are as follows: Bae et al. (50) and Santos et al. (51).

The summary for the RR phlebotomist team versus standard practice was 0.59 (95% CI 0.50–0.70, *P* < 0.01) for the BCC rate. There was no detectable heterogeneity (*I*^2^ = 0%). The RR effect estimates favored the phlebotomist team over standard practice for reducing contamination (nursing, physicians, and medical doctor interns) (Fig. 10). The overall contamination reduction was 41%. The intervention showed a significant benefit in lowering contamination (*P* < 0.01). This analysis showed no between-study heterogeneity; however, the results proved to be insignificant and do not include sampling error (*I*^2^ = 0%, *P* < 0.84).

### Sterile techniques

Question 4. Does using procedures that incorporate sterile techniques reduce blood culture contamination rates?

#### Recommendation

Institutions (facilities) should have a standardized procedure for using sterile technique for drawing BCs by peripheral venipuncture.

#### Literature review summary

A variety of studies examined using protocols that promoted sterile techniques for obtaining BCs (Table 5). Our analysis included randomized controlled trials (52, 53), cohort design (28), before–after design (54–56). “Sterile” kits were used (28, 52, 55, 56); two studies examined sterile versus non-sterile glove use (52, 53).

**TABLE 5:** Summary of studies that incorporated sterile protocols for obtaining BCs to reduce BCC rates

Study (reference)	Design	A or P^*a*^	Control group	Intervention group	Pre-BCC rate/control group (%)	Post-BCC rate (%)
Self et al. (56)	Before–after	A	Clean (non-sterile gloves, 2% CGH/70% isopropyl, non-sterile field)	Sterile (sterile gloves, 2% chlorhexidine, fenestrated drape, needle, checklist)	4.3	1.7
Hall et al. (54)	Before–after	P	Usual procedure, not sterile	Sterile (sterile gloves and sterile field)	3.9	1.6
Krajčinović et al. (55)	Before–after	P	Clean (non-sterile gloves, no well-defined sterile field, 70% isopropyl alcohol and 0.1% butanediol, no checklist)	Sterile (sterile gloves, sterile field, sterile gauze, 70% isopropyl alcohol and 0.1% butanediol, checklist)	16.4	7.6
Frota et al. (52)	Randomized control	A	Clean (non-sterile gloves)	Sterile (sterile gloves)	1.0, compared to a baseline of 6.1	1.0, compared to a baseline of 6.1
Kim et al. (53)	Randomized control	A	Optional sterile (sterile gloves worn only if needed)	Routine sterile (sterile gloves worn for every venipuncture)	0.9	0.5
Self et al. (28)	Cohort design	A	Full sterile process	Modified sterile process	2.71, compared to baseline of 4.83	0.91, compared to baseline of 2.51

^*a*^ A, adult; P, pediatric.

#### Evidence summary

**Fig 11:**
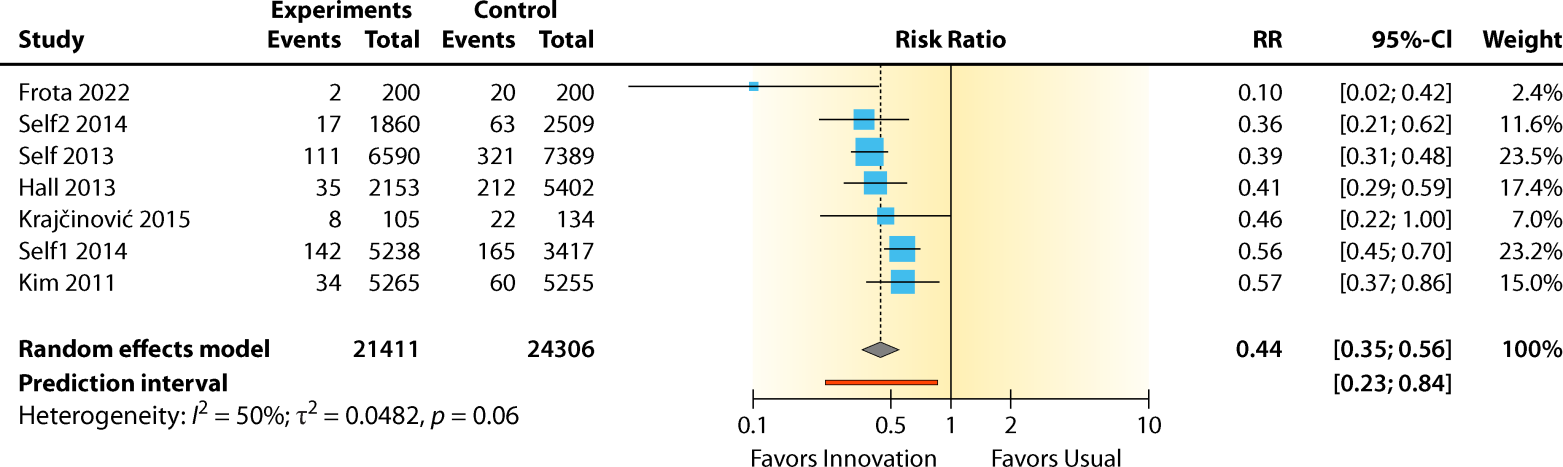
Forest plot of studies on using sterile protocols for reducing BCC rates. References are as follows: Frota et al. (52), Self et al. (28, 56), Hall et al. (54), Krajčinović et al. (55), and Kim et al. (53).

The summary for the RR for sterile technique versus standard practice was 0.44 (95% CI 0.35–0.56, *P* < 0.0001) for the BCC rate. Heterogeneity was not significant: *I*^2^ = 49.6% (95% CI 0.0%–78.6%, *P* = 0.103). The RR effect estimates favored sterile technique over standard practice for reducing contamination (Fig. 11). The overall contamination reduction was 56%. The intervention showed a significant benefit in lowering contamination (*P* < 0.01). However, this analysis has moderate and non-significant between-study heterogeneity, not including sampling error (*I*^2^ = 49.6%, *P* = 0.103).

### Education and training

Question 5. Do education and training as part of the overall blood culture policy reduce blood culture contamination rates?

#### Recommendation

Clinical laboratories are responsible for prescribing procedures for obtaining BCs and should work with institutional leaders to develop strong education programs for teams who draw BCs (laboratory phlebotomists, nurses, residents, and attendings).

#### Literature review summary

Most studies (Table 6) that examined the effect of education/training on reducing BCC rate used before–after study designs (27, 57–67). One study used a cohort design (68).

**TABLE 6:** Summary of studies using education and training interventions to reduce BCC rates^*a*^

Study (reference)	Design	A or P	Control	Intervention	Pre-BCC rate (%)	Post-BCC rate (%)
Al-Hamad et al. (58)	Before–after	A and P	Usual institutional practice	Workshops with questionnaire, PowerPoint, video, demo, and Q&A sessions	8.1	5.2
Al-Hamad (57)	Before–after	A	Usual practice in ED	Workshop with lecture, video, and demo	7.8	<3.0
Halstead et al.—A (27)	Before–after	A and P	Usual institutional practice	MDT and comprehensive education and training video	5.5	<1.6
Halstead et al.—B (27)	Before– after	A and P	Usual institutional practice	MDT and comprehensive education and training video	10.0	<2.0
Halstead et al.—C(27)	Before–after	A	Usual practice in ED	MDT, comprehensive education and training video	3.9	0.78–2.3
Halstead et al.—D (27)	Before– after	A, *P*	Usual practice in ED	MDT and comprehensive education and training video	7.4	2.2
Harding and Bollinger (59)	Before–after	A	Usual practice in ED	Individual training and feedback with retraining	1.8	1.0
He et al. (60)	Before–after	A	Usual practice in ICU	Standardized order, online learning, weekly reports, feedback, and training	4.5	2.6
Lin et al. (61)	Before–after	A	Usual practice in ED	Training on protocol and feedback with retraining	3.4	2.0–2.7
Marini and Truog (62)	Before–after	P	Usual practice in ED	Online module, supply kit with procedure, andgroup and individual training	2.1	1.4
Moeller (63)	Before–after	A	Usual practice in ED	Nursing shared governance with lab, revised collection procedure, and feedback	5.4	1.8
Murillo et al. (64)	Before–after	P	Usual practice in ED	Slide presentation and simulated observation	All cultures: 5.0 Frequent collectors: 4.1 Infrequent collectors: 8.0	All cultures: 4.9 Frequent collectors: 2.7 Infrequent collectors: 8.1
Park et al. (65)	Before–after	A	Usual practice	Clinical skills test, followed by separate institutional education program	Pre-clinical test intervention: 1.4 Pre-education intervention: 1.4	Post-clinical test intervention: 1.4 Post-education intervention: 1.0
Ramirez et al. (68)	Cohort	A	Usual practice in ICU	In-person education program	6.6	4.2
Roth et al. (66)	Before–after	A	Usual institutional practice	Structured presentations	2.6	2.2
Sánchez-Sánchez et al. (67)	Before–after	A	Usual practice in ICU	Training program in critical care units, standardized collection protocol, and MDT	14/100 cultures	5.6/100 cultures

^*a*^ A, adult; ED, emergency department; MDT, multidisciplinary team; P, pediatric.

#### Multidisciplinary teams

There was significant heterogeneity for the types of education/training programs used in these studies. In general, however, studies used multidisciplinary teams to develop education/training programs, procedure checklists, video or computer learning modules, and regular reporting of BCC rates at the unit level. Several studies reported sustainable results following education and training. Five studies discussed approaches to sustaining low BCC rates and were able to maintain low BCC rates for months to years (27, 57, 59, 60, 63). Education, training, feedback and retraining of collectors, annual reviews, and support of multidisciplinary teams were important aspects of these studies to instituting a sustainable plan to reduce BCC rates (27).

##### Evidence summary

**Fig 12:**
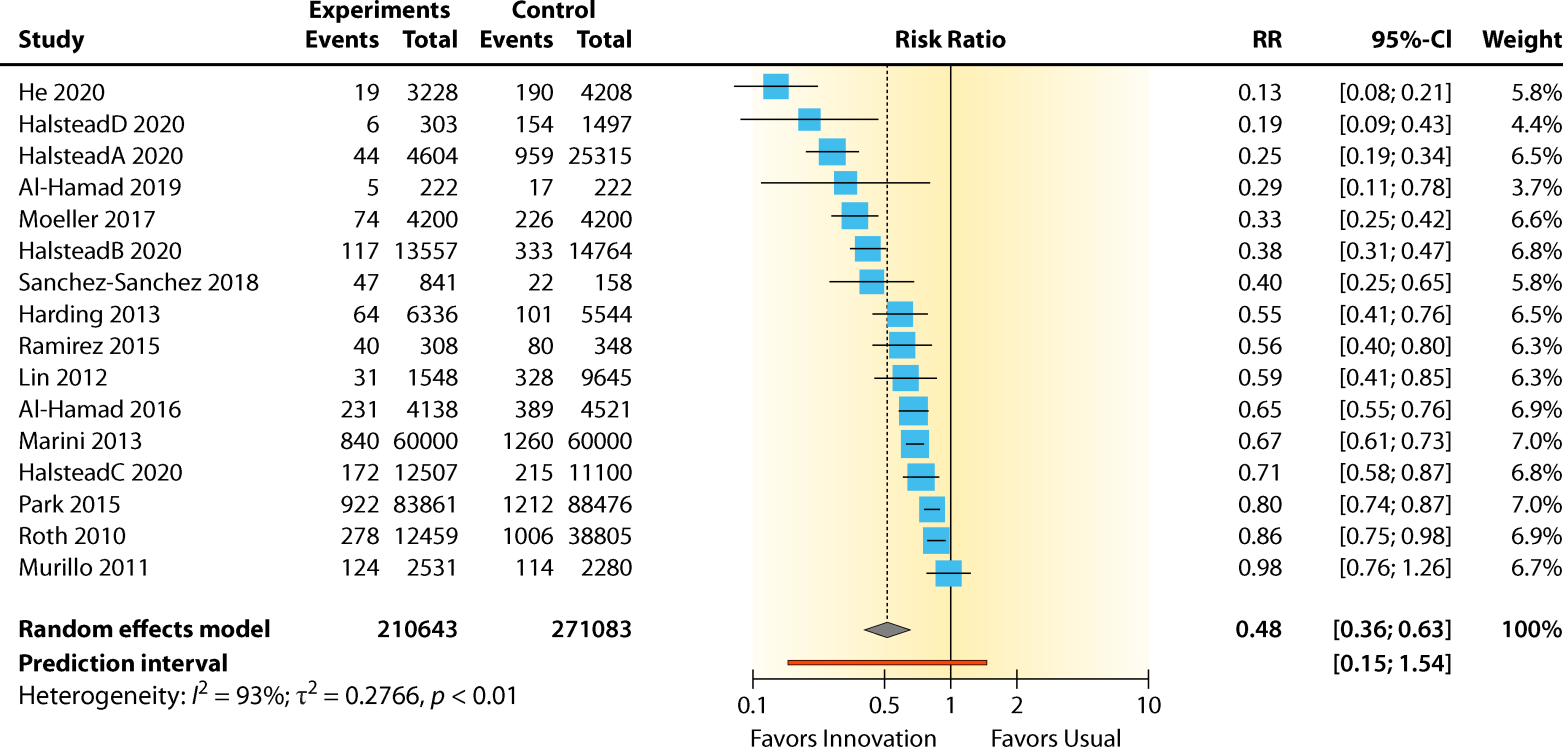
Forest plot of studies on the effect of education/training programs on reducing BCC rates. References are as follows: He et al. (60), Halstead et al. (27), Al-Hamad (57), Moeller (63), Sánchez-Sánchez et al.(67), Harding and Bollinger (59), Ramirez et al. (68), Lin et al. (61), Al-Hamad et al. (58), Marini and Truog (62), Park et al. (65), Roth et al. (66), and Murillo et al. (64).

The summary for the RR for education/training versus standard practice was 0.48 (95% CI 0.37–0.63, *P* < 0.01) for the BCC rate. A high heterogeneity effect was observed: *I*^2^ = 92.6% (95% CI 89.6%–94.8%). The RR effect estimates favored education/training over standard practice for reducing contamination (Fig. 12). The overall contamination reduction was 52%. The intervention showed a significant benefit in lowering contamination (*P* < 0.01). However, this analysis has a substantial between-study heterogeneity, not including sampling error (*I*^2^ = 92.0%, *P* < 0.01).

## ADDITIONAL OUTCOMES

Most studies reported BCC rates outcomes by unit. The meta-analytic results are presented in Table 2, and raw values for BCC change are reported in Table 7. There was a reduction in the risk of BCC across units of between 38% and 54% with the process improvement effort. Although there were differences between unit types, these differences were not statistically significant (*P* = 0.872).

**TABLE 7:** Blood culture contamination rate outcomes by hospital unit^*b*^

Unit	*n* studies	*n* samples	*n* BCC	RR	RR LCL	RR UCL	*I* ^2^	*I*^2^ LCL	*I*^2^ UCL	*P* value within unit
ED	18	152,339	5,061	0.49	0.36	0.66	86.8	80.5	91.0	<0.0001
General units	9	157,363	1,620	0.57	0.39	0.84	65.0	26.6	82.8	0.0036
ICU/heme/oncology	10	104,240	1,380	0.34	0.23	0.49	76.4	56.3	87.2	<0.0001
Pediatric units	12	243,618	5,015	0.56	0.41	0.76	66.6	38.7	81.8	0.0002
Combined units^*a*^	16	512,745	7,422	0.66	0.47	0.92	92.1	88.8	94.5	0.0135

^*a*^ .Combined units: outcomes from multiple hospital units.

^*b*^ ED, emergency department; *I*^2^, heterogeneity; LCL, lower confidence limit; RR, risk ratio; UCL, upper confidence limit

The high levels of heterogeneity are unsurprising since the range of strategies to decrease BCC varied widely.

To put the risk ratios in context, Table S2 provides the baseline and follow-up BCC rates, as well as the change from baseline to follow-up by unit.

When using the common quality indicator of 3% BCC rate as the upper limit of acceptability (4, 8), most facilities included in our analysis (75%) achieved <3% BCC rates. Just over 60% of facilities achieved BCC rates less than 2%, and over a quarter (28.6%) achieved <1% BCC rates. The frequency and proportion of units that were below 3% BCC are presented in Table 8.

**TABLE 8:** Achievement of different quality target rates for BCC

BCC target rate (%)	Baseline BCC rate		Follow-up BCC rate	
	***n***	%	***n***	%
≤3	23	41.1	42	75.0
≤2	10	17.9	34	60.7
≤1	2	3.6	16	28.6

## EVIDENCE OF PUBLICATION BIAS

There is little evidence for serious publication (small study) bias. The results of Peters et al.’s regression indicated no significant bias present (*t* = −0.98, df = 51, *P* = 0.333), and with the exception of one small study (38), the funnel plot is reasonably symmetric (Fig. S1).

## *A PRIORI* HYPOTHESES: IMPLEMENTATION SCIENCE PRINCIPLES

In addition to whether a discrete improvement effort (e.g., 3% chlorhexidine gluconate versus 10% povidone–iodine for skin disinfection) was effective, we questioned whether there were process improvement principles that could explain success reducing BCC, regardless of the particular type of change made. We suggest that efforts to decrease BCC within health organizations can best be viewed via an implementation science framework (16, 23): changes are typically multifaceted (requiring technology, time, and training to implement new practices), depend on stakeholder’s buy in to the change process, and require both resources and know-how to bring about the targeted change.

### Hypothesis 1: pressure for change and BCC rate

Implementation success depends on, among other things, the “tension for change” (23). This is the degree to which stakeholders believe that the current situation is unacceptable or untenable. In light of this, we would expect there to be a relationship between the initial BCC rate and the improvement in the risk of BCC: higher baseline BCC rates would be associated with lower risk ratios (indicating greater improvement in the reduction of BCC from standard to process improvement). In other words, whatever improvement strategy institutions seek to implement, they are more likely to be successful if there is agreement that the problem is pressing.

In our sample, 41.1% of the facilities reported a base BCC rate of ≤3% (Table 8); 36.7% reported base BCC rates between 3% and 6%, while 24.5% reported base rates of >6% (with six studies reporting base rates of 10% or higher).

Our analysis provided weak support for the hypothesis that the level of improvement was associated with a greater tension for change (as operationalized by the baseline BCC rate). Meta-regressing the RR of the overall facility BCC rate change on the baseline BCC rate, while the slope of the baseline BCC was −2.7 (95% CI −6.59 to 1.19), indicating greater reduction in BCC rate, the baseline rate explained a relatively small amount of variance (2.3%) of the variability in RR between studies (*P* = 0.173) (Fig. S2).

### Hypothesis 2: BCC reduction as part of a larger process improvement initiative

Drawing from research identifying predictors of quality improvement success (69–71), we hypothesized that there would be a difference in the effectiveness of process improvement efforts that were part of a quality management/quality improvement effort. Many studies explicitly stated that the change in BC collection was a direct response to a higher BCC rate than was desirable. In response, these institutions formed committees or task forces to come up with a comprehensive solution to the problem. Our hypothesis was motivated by the implementation science literature, which suggests that broad institutional change may be more effective with widespread stakeholder buy-in, etc.

Thirty-seven studies indicated that the change in blood collection practices was part of a larger QMQI effort. Eighteen studies did not indicate that the results were part of a QMQI effort. The results of the subgroup analysis indicated that BC collection changes which were part of a QMQI effort showed a 56% (95% CI 45.5%–64.0%) reduction in BCC, compared to a 43% (95% CI 21%–59%) reduction of BCC for efforts not explicitly part of a QMQI effort (*P* = 0.216). There was still substantial to considerable heterogeneity across the subgroups (86%–91%), indicating wide variation in approaches and differences in local contexts.

### Hypothesis 3: BCC reduction and training intensity

Changes in equipment or processes require that staff be equipped with the knowledge and training to successfully implement these changes. We hypothesized that we should see a greater reduction in BCC with a greater level of training intensity. We classified studies in terms of the level of intensity as follows (lowest to highest intensity):

- No mention of education, training, or feedback.
- Report of education only (didactic sessions with no hands-on training).
- Report of training sessions [hands-on practice with the new equipment or process (e.g., checklists)].
- Report of on-going feedback (continued monitoring) on performance of the new process or use of the new equipment.

Because author descriptions were sometimes less thorough than hoped (sometimes providing no information on how clinicians were educated on new procedures or devices), we collapsed the above categories into “low-intensity” versus “high-intensity” training interventions. The results are presented in Table 9. A meta-regression of the relationship indicated that training intensity explained 4.7% of the heterogeneity (*P* = 0.093), with high-intensity training showing a 57% reduction in contamination rates compared with only a 40% reduction with low-intensity training.

**TABLE 9:** BCC risk reduction by training intensity

	*n* studies	RR	RR LCL	RR UCL	*I* ^2^
Low intensity	20	0.60	0.44	0.81	87.1
High intensity	33	0.43	0.35	0.53	90.8

**Fig 13:**
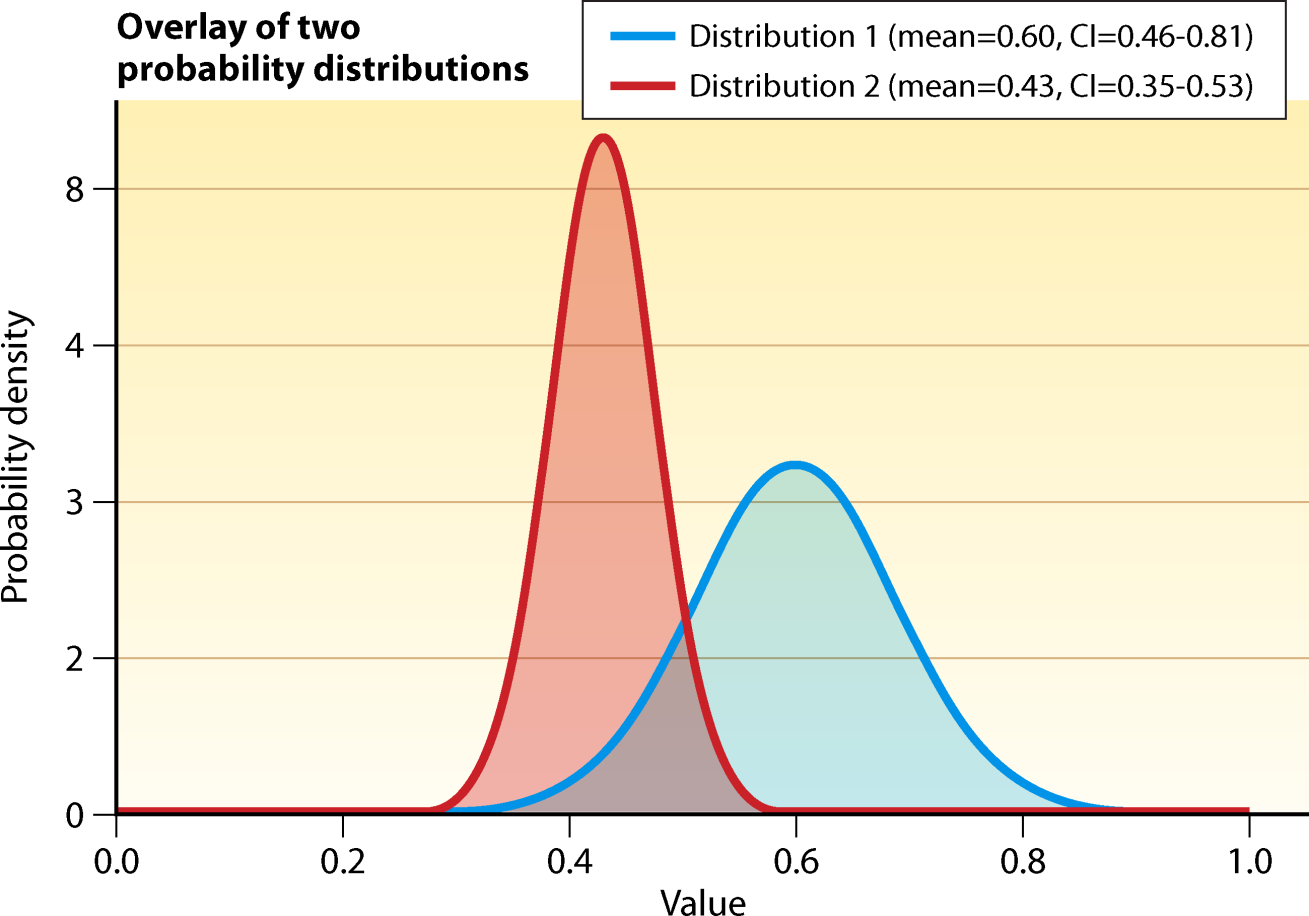
Comparison of probability distributions of BCC reduction rates for high-intensity versus low-intensity training interventions.

Although the difference between the two distributions did not reach *P* < 0.05, a plot of the probability distributions demonstrates that the likelihood of a greater reduction in BCC is higher with high-intensity (Fig. 13, distribution 2) compared to low-intensity (Fig. 13, distribution 1) educational interventions. In other words, while it is possible to achieve comparable BCC reductions with low-intensity training, it is much less likely.

Finally, recognizing that many organizations integrated high-intensity training within larger QMQI process improvement strategies, we compared results in reduction of BCC rates between organizations that used both process improvement strategies compared to those that only used one strategy or no process improvement strategies. The results (Table 10) show a statistically significant improvement in BCC rates for organizations that implemented one or both of the process improvement strategies compared to those that reported no process improvement strategy (*P* = 0.032). While organizations that used either or both process improvement strategies reduced BCC rates by an average of approximately 56%, those that used no process implementation strategies reduced BCC rates by only 15%.

**TABLE 10:** Comparison of BCC reduction rates for organizations that used two, one, or no process improvement strategies

	*n* studies	RR	RR LCL	RR UCL	*I*^2^ (%)
Both QAQI and high-intensity training	20	0.44	0.35	0.56	92.9
One process improvement strategy	20	0.43	0.32	0.58	77.1
No process improvement strategy	9	0.85	0.54	1.34	88.6

## EXPLORATORY HYPOTHESES: FOCUS, PROFESSION, AND YEAR OF PUBLICATION

While not part of the a priori hypotheses set, we explored two additional explanations for the typically high heterogeneity explained above:

- Whether the specific improvement focus of the intervention (education focused, procedure focused, solution focused, device focused, and profession focused) made a difference in terms of BCC rate.
- Whether the year of publication of the BCC improvement report is related to the BCC rate.

We provide these analyses to inform future guideline projects rather than explanatory in any way. Even though our formal hypotheses all demonstrated significant reductions in the between-study heterogeneity, substantial heterogeneity remained for most tests, reflecting the diversity of context-embedded improvement processes. Future guidelines may seek to answer different or more focused questions.

### Improvement focus and BCC rate

While nearly all studies identified explicitly used multicomponent interventions, they differed in the primary focus of the intervention. We classified intervention focus into the following types:

- **Education focused:** Whatever procedure, device or solution was currently in place, the primary intervention effort focused on improving staff knowledge/skills in executing BC collection to minimize BCC.
- **Procedure focused:** Improvements that focused on the procedures used to collect blood samples (e.g., checklists, corrective action, bundles, and sterile techniques)
- **solution focused:** improvements that focused on the comparison using different disinfectant solutions.
- **Device focused:** Improvements that focused on integrating a new blood collection device (e.g., diversion devices, catheter, and peripheral versus arterial)
- **Profession focused:** Improvement that focused on who collects blood (e.g., nurses versus phlebotomists)
- **Combined focus:** Improvements that expressly combined any number of the above.

The results of the subgroup meta-analysis are presented in Table 11.

**TABLE 11:** Reduction in blood culture contamination by intervention focus

Focus	*n* studies	RR	RR LCL	RR UCL	*I* ^2^
Education focused	14	0.52	0.41	0.68	91.7
Procedure focused	9	0.48	0.27	0.87	92.8
Solution focused	11	0.53	0.31	0.90	64.9
Device focused	8	0.53	0.50	0.70	84.2
Profession focused	2	0.59	0.17	2.04	0.0
Combined focus	9	0.41	0.27	0.60	89.9

As can be seen in Table 11, there was no difference in the improvement of BCC by intervention focus (*P* = 0.393). Consequently, classifying by intervention focused explained none of the heterogeneity. This may indicate either that the particular focus of the improvement effort is less important than the explanatory process improvement efforts we describe above, or that the particular focus depends heavily on the needs of the local context—that is, the key between effort and improvement may be more a matter of “fit” than a particular approach.

This analysis may also suggest that very focused clinical practice guideline comparisons (e.g., “is X solution or device “better” than some comparator?”) may be of little benefit for future guidelines since whether or not a particular approach is better than an alternative may depend very heavily on the context in which it is being implemented.

### Publication year and BCC rate

The year of publication was significantly associated with RR, explaining 10.92% of the heterogeneity (*P* < 0.001). Predictably, heterogeneity remained considerable (95.0%). This improvement over time not only is likely related to enhanced publication standards but also may indicate a process of institutional learning of good practices related to reduction of BCC (see Fig. S3).

## SENSITIVITY AND SUBGROUP ANALYSES: RISK OF BIAS

Because of the diversity of intervention and differences in contexts (different units, different baseline BCC rates, and different populations), heterogeneity among the studies remained high. Thus, we carried out three additional analyses to identify potential reasons for this heterogeneity. We carried out the following analyses: (i) an outlier analysis (dropping outlying studies and examining differences between overperforming and underperforming interventions), (ii) an analysis of the effect of ROB, and (iii) an analysis of the effect of study design.

### Sensitivity analyses: outlier analysis

A sensitivity analysis was performed with outlying studies omitted from the analyses. Interventions could be either overperforming (i.e., where the lower 95% CI of the study effect was above the upper 95% CI of the pooled effect) or underperforming (i.e., the upper 95% CI of the study effect was below the lower 95% CI of the pooled effect). The results of the outlier sensitivity analyses are reported in Table S3.

For ED, ICU/heme/oncology, and pediatric units, removal of the outliers made little substantive difference (i.e., RRs ± 2% of original estimate). However, for general units and combined units, the effect of the intervention decreased (i.e., the risk of BCC was higher when outliers were removed). For both units, this was due to the removal of overperforming interventions: Ge et al. (43) for the general units and three of the four sites from the Halstead et al. (27) study.

Details on the characteristics of over- and underperforming interventions are described in Table S4.

## EFFECT OF RISK OF BIAS AND STUDY DESIGN

### Risk of bias

We present the ROB subgroup analysis without regard to unit in Fig. S4. Our analysis finds that there are only slight differences in the reduction of BCC risk between ROB groups, with the low ROB group having the largest (53%) reduction in BCC. However, there was no significant difference between group differences (*P* = 0.96), and heterogeneity remained considerable except for the moderate-risk studies.

### The effect of study design

We additionally carried out a subgroup analysis to determine whether there were differences in effect based on study design. While there was a large difference between cohort design studies (RR = 0.75, 95% CI 0.31–1.83) and before–after (RR = 0.45, 95% CI 0.39–0.53) and controlled studies (RR = 0.49, 95% CI 0.31–0.73), these differences did not reach statistical significance (*P* = 0.5369), in large part because of the imprecision of the cohort study effect size.

It is noteworthy that the reduction in BCC was similar between the lower-risk before–after studies (54.6% reduction in BCC) and the higher-risk controlled design studies (50.9% reduction in BCC). Meta-epidemiologic research indicates that inadequate concealment and blinding (and overall higher risk) tend to be associated with larger effect sizes (72). These were problems noted in the controlled studies included in this analysis. However, from the perspective of process improvement implementation, where stakeholder buy-in and a culture of improvement (quite the opposite of “uninterested” research) are key to success (24, 73), what would count as “bias” in a classic RCT would be considered desirable from an implementation science perspective.

## DISCUSSION

While pre-analytic, analytic, and post-analytic factors contribute to the overall cycle of reporting BC results (8), it is the pre-analytic phase that has a significant impact on BCC rates. This phase includes the body site where BCs are obtained (i.e. peripheral draw and catheter draw), preparation of the body site with antiseptics (i.e. alcohols, chlorhexidine, and povidone–iodine), and maintaining sterile conditions while obtaining BCs.

Previous studies on reduction of BCC identified a number of practices that may be important for minimizing BCC (4, 5, 9). Furthermore, BCs are overutilized, and although there are no benchmarks for utilization (1, 5), reduction of unnecessary BC will reduce potential harm associated with contaminated BCs (5, 74).

The current study expands the results of the previous ASM meta-analysis by Snyder et al. (13). In the Snyder et al. study, venipuncture was compared with catheter-drawn BCs; phlebotomy teams were compared with non-phlebotomy trained personnel; and pre-packaged preparation kits for drawing BCs were compared with usual non-packaged supplies. We expand their findings by examining chlorhexidine versus other skin disinfectants for skin preparation, use of diversion devices to reduce BCC, use of sterility protocols versus standard practice for obtaining BCs, and education/training and its effect on reducing BCC.

### Skin antisepsis

Preparation of the venipuncture site with antiseptic agents is one of the most critical steps in the BC collection procedure. Many studies promote the use of chlorhexidine gluconate and have found it to be superior or non-inferior to iodine containing agents. Maiwald and Chan (75) performed a systematic review and meta-analysis and concluded that CHG with alcohol was superior to povidone–iodine alone, but no evidence that CHG alone was any more effective. We only examined studies that included chlorhexidine in our systematic review. Most studies (*n* = 6) used CHG–alcohol, and some used CHG alone (*n* = 4). Most studies compared CHG–alcohol or CHG alone with povidone–iodine alone; one study used tincture of iodine (40); and one used alcohol alone (36). We found that using chlorhexidine containing antiseptics, grouped as a whole, resulted in a significant reduction in BCC rates compared with standard procedures. Use of alcoholic CHG or alcoholic tincture of iodine was recommended by Doern and colleagues in their expert review (4).

### Diversion devices

Diversion devices for reducing BCC rates were assessed in this current study and included six studies. Diversion devices allow the provider obtaining the BC to withdraw a few milliliters of blood after the initial venipuncture for discard, then draw blood into the appropriate BC bottles. By removing the initial blood sample, this removes the skin plug that may be associated with BC contamination. This approach has been used in transfusion medicine for decades to reduce blood product contamination (76).

Diversion devices can be simply employed by using a sterile blood tube (without additives) or a pre-packaged commercial diversion device. We found that use of diversion devices resulted in a significant reduction in BCC rates compared with no diversion device. The six studies on diversion devices used either a commercial diversion device (*n* = 2) or a sterile blood collection tube prepared on site (*n* = 4). For these studies, skin antisepsis included chlorhexidine–alcohol (*n* = 3), CHG or povidone–iodine (*n* = 1), and 70% isopropyl alcohol (*n* = 1), or the type of skin preparation was not specified. We conclude from these studies that the type of diversion device was not critical, as long as a sample of blood was removed prior to collecting BCs. Our results confirm a recent study using a commercial diversion device (77) and are supported by a systematic review and meta-analysis of using BC diversion devices by Callado et al. (78) that found a significant reduction in BCC rates using diversion devices.

Several concerns might be raised about using diversion devices including cost and potential harms. Most of the studies included in our analysis used a sterile tube as the diversion tube, and using sterile tubes is an inexpensive alternative to using commercially packaged diversion devices. We did not identify any studies that directly compared commercially packaged diversion devices with sterile tubes. Considering that there is no evidence that commercial devices are superior to using sterile tubes, the additional cost for implementing a sterile tube diversion device would be minimal. There was a range of blood volume discarded in the six studies from 1 to 7 mL of discard per draw. While small volume discards (<2 mL) are likely to cause little harm to patients, discarding larger volumes of blood (e.g., 7 mL in the Syed et al. study) might contribute to the development of iatrogenic anemia in patients with prolonged hospital stays and frequent BCs (79). We are not aware of any studies comparing the volume of blood drawn for diversion and relationship to contamination rates. In addition, providers using a diversion tube must adhere to specimen (tube) labeling requirements to avoid unlabeled discard tubes being used for testing and/or assigned to the incorrect patient if not disposed properly (80).

### Sterile technique

It seems intuitive that sterile technique should be used when obtaining BCs. However, there is no standard for what is considered sterile technique for this procedure. There are numerous components to the action of obtaining BCs, including skin antisepsis (various agents), locating a suitable vein for peripheral access, gloving (sterile versus non-sterile), preparation on BC bottles, and inoculation of bottles.

We examined studies that focused on using a sterile procedure when obtaining BCs on BCC rates. As a group, using a sterile procedure resulted in a reduction in BCC rates. However, we could not compare specific components in most studies comprising sterile procedures to determine whether all components of each study were necessary. Two studies (52, 53) did examine using sterile versus non-sterile gloves and showed reduction in BCC rates from 43% (53) to 90% (52).

### Phlebotomy teams

Phlebotomy teams have been an important part of the clinical laboratory’s ability to obtain blood samples for daily chemical and hematologic analysis in hospitalized patients. In general, phlebotomy teams have been focused on drawing blood samples on large numbers of patients during early morning hours. However, BCs are a time-intensive and specialized procedure that may not be amenable to logistics of the daily high-volume blood draws performed by phlebotomists. Despite the logistical issues with drawing BCs, many institutions have dedicated phlebotomists available to draw BCs. Numerous studies including the two in our analysis show that BCC rates are lower than those drawn by other providers. In the 2012 meta-analysis by Snyder et al. (13), all five studies that examined the role of phlebotomy teams for reducing BCC rates favored phlebotomy teams over non-phlebotomy teams. The two studies in our paper (50, 51) focused on drawing BCs via venipuncture only. Lower BCC rates were reported with venipuncture by trained phlebotomists. However, the studies were limited by the lack of details for education and training of the interns or nurses drawing BCs. The emergency room was excluded in the Bae et al. study (50) as they were normally drawn by laboratory phlebotomy, nurses or interns. The studies mentioned referred to as a “dedicated” laboratory phlebotomy. The following section on Education and Training will show how appropriate training with follow-up, re-training when necessary, results in low BCC rates well below the guideline recommended level of <3%. In most cases, the levels achieved were <2%.

### Training/education

We examined the role of training and education in reducing BCC rates. Training and educational activities can vary from simple to extensive programs. Components of training/education programs mostly had in common the use of multidisciplinary teams to develop programs. Online or in person presentations, regular reporting of BCC rates, feedback was common in these studies. As shown in Table 9, we found that high intensive training resulted in improved reduction of BCC rates over low intensive training. Roadblocks to developing effective programs concern time constraints of trainers and the ability to train numerous individuals throughout an institution. Many approaches to training providers are used in healthcare institutions, but programs can be optimized by using the “train the trainer” model. Several articles used a train the trainer plan that would allow more “experts” in the nursing field. Following feedback and including results and management and administration oversight are very helpful (27). In a four-independent hospital study, one hospital showed that a phlebotomy team was needed to lower rates; however, the other three resulted in low rates for all wards without the use of phlebotomy teams.

### Blood culture contamination rates

In a subanalysis of studies that achieved below 3% BCC rates (see Table 8), we found approximately three-fourths of the outcomes from the studies included in our meta-analysis resulted in BCC rates of <2%, and approximately one-third resulted in <1% BCC rates. Although the BCC rate of ≤3% has been used for many years as a benchmark for BC quality, a recent survey suggested that a majority of survey respondents achieved contaminations well below 3% and supports the goal of developing new lower BCC rate benchmarks (10), and others promote a BCC of ≤1% as achievable (4, 8). Given the above findings, however, definitions for BCC varied among the studies included in our analysis.

### Process improvement

We are not aware of any prior study to identify the importance of process improvement principles in improving BCC rates.

Interestingly, our analyses revealed that the particular focus of the improvement effort (e.g., device, solution, and process; see “Description of studies,” above) was less important for success than:

1. **The perceived need for improvement:** baseline contamination rates were positively associated with greater reduction in BCC (see “Hypothesis 1,” above),
2. **The improvement being part of a larger quality improvement effort:** improvement efforts that were described as part of a larger comprehensive quality management effort were more successful than those that were not (see “Hypothesis 2,” above),
3. **Staff training intensity:** higher-intensity training (involving education, training, and feedback) was more successful at reducing BCC rates (see “Hypothesis 3,” above).

Whether the practices identified by Doern and colleagues (4) are indeed “essential” is an open question. Our findings suggest that efforts to reduce BCC rates may not need to include all items identified by Doern et al., so long as the process for improvement is perceived as needed, part of a comprehensive effort, and involves more intensive staff training and monitoring.

Our findings suggest practical guidance for lab managers and administrators seeking to reduce their unit or organization’s BCC rates.

**Approach 1.** Before beginning the process improvement effort, make sure that key stakeholders (e.g., clinicians drawing blood) understand the importance of the effort. Making the changes will require buy-in from clinical staff to implement changes consistently and overcome entrenched habits that have led to unacceptable contamination rates. Leader sponsorship and acceptance are particularly important in this regard (16).
**Approach 2:** Prior to implementing the improvement effort, planning—involving both leadership and clinical staff—is crucial. Process improvement efforts require substantial and meaningful contributions of personnel, expertise, money, equipment, or other important resources. Thus, coordination is crucial to manage the interdependence of all parties involved (23).
**Approach 3:** Successful efforts to reduce BCC require not only “know-how” but also shared motivation and perspective. This implies that merely “showing” clinicians how to make an improvement may be of limited value. Rather, more intensive efforts that include education, hands-on training, and, crucially, monitoring and accountability have a higher likelihood of success.

As we noted above, the success of an intervention effort depends highly on the local context. Thus, the choice of which elements to implement should be aligned to organizational needs and goals. Ensuring “fit” between the intervention and the organizational context may likely require alignment with organizational needs and metrics, alignment with organizational resources and capabilities, and alignment with organizational priorities and culture. What may “work” for one unit or organization or system may not be ideal in another context.

A number of toolkits exist to help administrators and stakeholders implement planned process improvements within the context of a larger quality management quality improvement framework (e.g., see the Institute for Healthcare Improvement Tools at https://www.ihi.org/resources/tools). Additionally, we encourage stakeholders planning to implement new multicomponent BCC prevention practices to familiarize themselves with some of the major implementation frameworks, including the Consolidated Framework for Implementation Research, the Theoretical Domains Framework, and the MUSIQ.

### Effect estimates, heterogeneity, and confidence in the findings: what to expect

The reader might reasonably ask what sort of improvement could be expected by adhering to the process improvement principles we identified. For instance, holding the particular implementation effort constant, should a hospital or unit really expect only a 5.2% greater reduction in BCC rates when the improvement effort is part of a QMQI versus not (e.g., 53.2% reduction in BCC for QMQI efforts, compared to a 48% reduction of BCC for efforts not explicitly part of a QMQI effort)? Or would we always expect to see at least a 24% greater reduction in BCC rates when high-intensity training is used (compared to low-intensity staff training)?

The short answer is no. As reported above, heterogeneity across analyses remained high, indicating that differences in local efforts can have a large effect on the success of an implementation effort. Additionally, as is evident from Table S4, context matters—similar efforts in different types of units may not perform equally well—some efforts “overperformed” the average effects, while some “underperformed.” The key to understanding the analyses above is not to expect a precise level of improvement,but to identify key features that are likely to lead to greater success in reducing BCC rates.

Even though we do not encourage readers to view the above effects as precise levels of improvement to be expected, we do have high confidence in the general findings. Using the GRADE criteria to evaluate the strength of evidence (21), the team concluded that there was high confidence in the findings across two of the study design types (before–after and controlled trial designs), holding all else equal (Table S1).

## LIMITATIONS

The primary challenge of this study was the necessity of examining multicomponent interventions to reduce BCC rates. The traditional synthesis approach of comparing discrete practices (e.g., diversion device versus no diversion device) when all else is equal was not feasible since “all else” was never equal. Studies mixed different improvement components and implemented them in different ways and in different contexts. Thus, as we noted above, the exact local results that might be expected from any particular effort to reduce BCC rates are likely to vary somewhat from the findings reported in this analysis. However, in the presence of complex interventions, splitting comparisons into finer, purer, and more discrete comparisons is not appropriate since an adequate treatment of complex interventions requires the integration of heterogeneous data and seeking to “explain” some of that heterogeneity via planned subgroup analyses and meta-regression.

Another limitation is our inability to speak to predictors of sustainability. Although several studies reported sustainability of reduced BCC rates over time (27), even the before–after studies (which typically had a multiyear timeline) generally did not report longer-term (e.g., >5 years) outcomes. We do not know whether the results reported in the different studies examined here were able to be sustained after the publication of the reports.

Lastly, we show that various interventions significantly reduced BCC rates; however, a lack of a standard definition for BCC could affect results.

## CONCLUSIONS

Our analysis of 49 studies (*n* = 958,387 observations) reporting on different types of efforts to reduce BCC rates found five interventions that all resulted in reducing BCC rates. The particular approach to reducing BCC rates seems to matter much less than the approaches used to implement these improvements. A perceived need for the improvement, along with high-intensity staff training within the context of a comprehensive quality improvement effort, was all more strongly associated with greater reductions in BCC rates than merely changing devices or techniques of blood sample collection. There does not appear to be a simple silver bullet for reducing BCC rates. Rather, administrators and stakeholders seeking to reduce BCC rates should implement the targeted improvement by adhering to evidence-based principles of successful quality improvement.

## FUTURE RESEARCH NEEDS

- Develop a standardized national definition for BCC.
- Studies on the sustainability for achieving lower BCC rates.
- Best practices for education and training, including how to monitor BCC rates and provide feedback.
- How BCC impacts National Healthcare Safety Network definitions.
- Additional studies in pediatric populations.

## SUPPLEMENTAL MATERIAL

Supplemental Figures S1 to S4 cmr.00087-24-s0001.docx Fig. S1 (funnel plot of all studies of publication bias), Fig. S2 (relationship of risk ratio of BCC improvement to base BCC rate), Fig. S3 (meta-regression of risk ratio of BCC improvement and year of publication), and Fig. S4 (risk of bias subgroup analysis without regard to unit).

Supplemental Tables S1 to S4 cmr.00087-24-s0002.docx Table S1 (GRADE evidence summary table), Table S2 (change between baseline and follow-up blood culture contamination rates by unit), Table S3 (outlier sensitivity analysis by unit), and Table S4 (study detail on outlying over- versus underperforming interventions).

ASM-BCC Guideline Documentation cmr.00087-24-s0003.docx Details of the search history, search strategy, records identification, screening, and methodology.

BCC Meta-Analyses cmr.00087-24-s0004.pdf Codes used for the main analyses, hypothesis tests, sources for heterogeneity, analysis for GRADE, interventions and hypothesis tests.

BCC Characteristics of Included Studies cmr.00087-24-s0005.xlsx Characteristics of included studies.

ASM does not own the copyrights to Supplemental Material that may be linked to, or accessed through, an article. The authors have granted ASM a non-exclusive, world-wide license to publish the Supplemental Material files. Please contact the corresponding author directly for reuse.

## APPENDIX

**Blood culture contamination** – Indicated when indigenous skin bacteria are isolated from a single blood culture bottle and is not indicative of a true bloodstream infection. There is no standard definition for what constitutes blood culture contamination, but typically, isolation of one of the following bacteria from a single blood culture bottle (or set) out of multiple blood culture sets in a 24-hour period may be defined as a contaminant. Coagulase negative staphylococci, *Corynebacterium* spp. (aka diptheroid), *Bacillus* sp. (not *B. anthracis*), *Micrococcus* sp., certain alpha hemolytic *Streptococcus* sp., and *Cutibacterium acnes*.

**Blood culture contamination rates** – Usually expressed as a percentage of all blood cultures (sets) with a blood culture contaminant, drawn over a given period of time. The BCC rate is used as a quality measure for using proper technique in obtaining blood cultures. A rate of <3% is commonly used as the upper limit of acceptable practice, although many groups are recommending a lower rate as an acceptable quality measure.

**Diversion device** – Either a commercial or non-commercial device (blank blood tube) used for the first few milliliters of blood being drawn for a blood culture and then discarded. The first few milliliters of blood generally contain the initial skin plug that contributes to blood culture contamination.

**GRADE** Grading of Recommendations, Assessment, Development, and Evaluations (GRADE) is a transparent framework for developing and presenting summaries of evidence and provides a systematic approach for making clinical practice recommendations. It is the most widely adopted tool for grading the quality of evidence and for making recommendations with over 100 organizations worldwide officially endorsing GRADE (https://bestpractice.bmj.com/info/us/toolkit/learn-ebm/what-is-grade/) (https://training.cochrane.org/grade-approach).

**Heterogeneity** – The proportion of variance in a meta-analysis attributable to differences (i.e., in methodology, sample characteristics, and procedures) between studies rather than within study sampling error. High levels of heterogeneity may be interpreted to indicate coherent subpopulations within a sample of studies.

**Implementation science** – The scientific study of methods and processes by which practices (often evidence-based) are put to practical use by policymakers, clinicians, administrators, etc. It is based on the recognition that the methods by which policies are put into practice are integral to the success or failure of the uptake or institutionalization of those practices.

**PRISMA –** Preferred Reporting Items for Systematic Reviews and Meta-Analysis, a flowchart representing the process used to identify and select published data for a systematic review or meta-analysis.

**Publication bias** – Systematic bias toward positive effects that result from the lack of publication of small studies that show no effect, especially when small studies that show a positive effect are preferentially published; also called small study bias.

**Random effects meta-analysis** – Two types of models in meta-analysis are common: fixed effects models and random effects models. Fixed effects models assume a homogeneous underlying population and one true effect size across this population. Random effects models are used when multiple subpopulations are suspected and allow for differences in treatment or exposure effects from study to study.

**ROB** – Risk of bias, an estimate of the threats to study internal validity. Specific criteria (e.g., selection bias, performance bias, detection bias, and reporting bias) are systematically and transparently evaluated to generate a judgment of the trustworthiness of the findings of a research report.

**Skin antisepsis** – The procedure used to prepare the skin prior to venipuncture for the blood culture technique. A variety of skin antiseptics include chlorhexidine, povidone–iodine, and isopropyl alcohol.

**SRDR+** – Systematic Review Data Repository Plus (SRDR+) is a data collection and warehousing platform supported by the US Agency for Healthcare Research and Quality. Among other things, SRDR+ functions as a collaborative web-based tool for systematic review data extraction and sharing.

**Typology** – A classification scheme the divides a phenomena into basic types. Items within classificatory types are known as tokens.
