# Supplementary material for: American Society for Microbiology evidence-based laboratory medicine practice guidelines to reduce blood culture contamination rates: a systematic review and meta-analysis
Source: Clin Microbiol Rev. 2024 Nov 4;37(4):e00087-24. doi: 10.1128/cmr.00087-24 (PMC11629619; doi:10.1128/cmr.00087-24)
Supplement: Supplemental Figures S1 to S4 — Fig. S1 (funnel plot of all studies of publication bias), Fig. S2 (relationship of risk ratio of BCC improvement to base BCC rate), Fig. S3 (meta-regression of risk ratio of BCC improvement and year of publication), and Fig. S4 (risk of bias subgroup analysis without regard to unit). [file cmr.00087-24-s0001.docx]

Figure S1. Funnel plot of all studies of publication bias


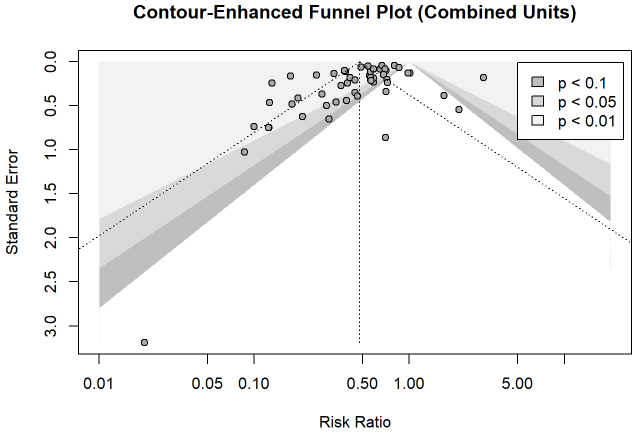


There is little evidence for serious publication (small study) bias. The results of Peter’s regression indicated no significant bias present (t=-0.98, df=51, p=0.333), and with the exception of one small study (Nuntnarumit 2013) the funnel plot is reasonably symmetric.

Figure S2. Relationship of Risk Ratio of BCC Improvement to Base BCC Rate


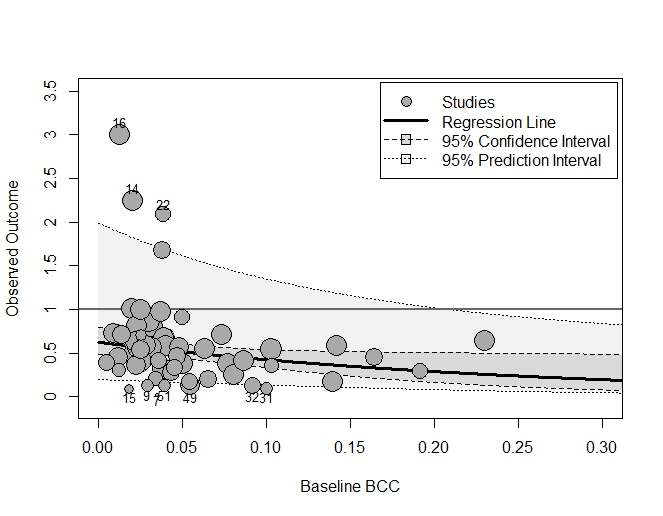


Meta-regressing the RR of the overall facility BCC rate change on the baseline BCC rate, while the slope of the baseline BCC was -2.7 (95% CI: -6.59, 1.19), indicating greater reduction in BCC rate, the baseline rate explained a relatively small amount of variance (2.3%) of the variability in RR between studies (p= 0.173)

Figure S3. Meta-regression of Risk Ratio of BCC Improvement and Year of

Publication


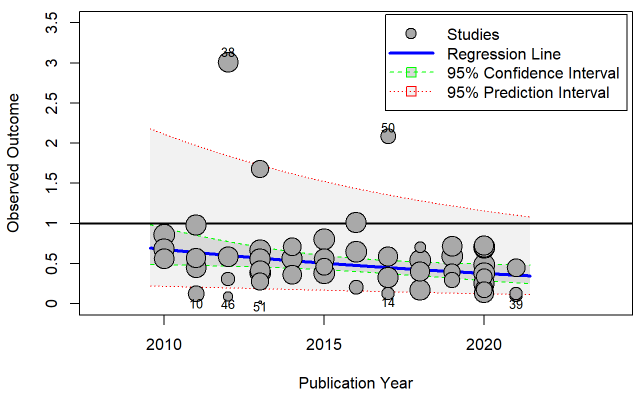


The year of publication was significantly associated with RR, explaining 10.92% of the heterogeneity (p<0.001). Predictably, heterogeneity remained considerable (95.0%). This improvement over time is likely related to not only enhanced publication standards but may indicate a process of institutional learning of good practices related to reduction of BCC.

Figure S4. Risk of bias subgroup analysis without regard to unit (1-50)


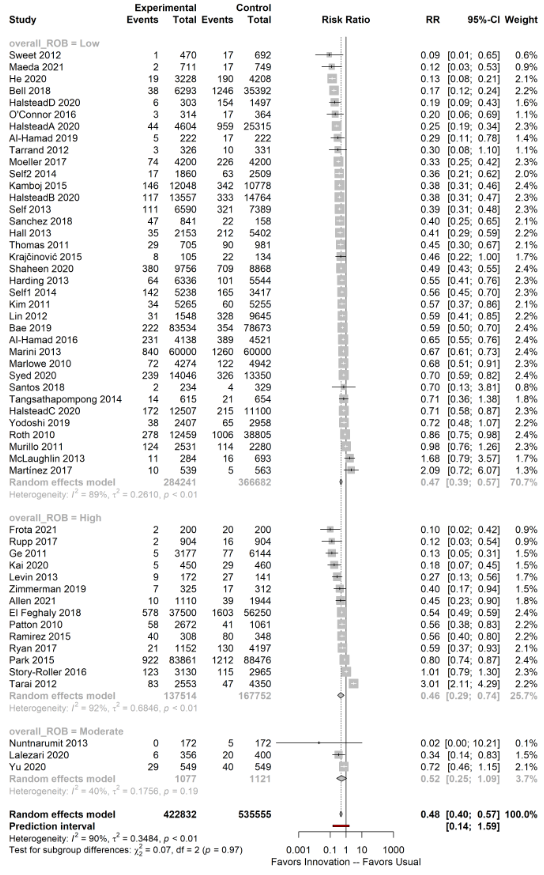


Our analysis finds that there are only slight differences in the reduction of BCC risk between ROB groups, with the low ROB group having the largest (53%) reduction in BCC. However, there was no significant difference between group differences (p= 0.96) and heterogeneity remained considerable except for the moderate risk studies.

# References

1. Al-Hamad A. 2019. Successful Reduction of Blood Culture Contamination in an Emergency Department by Monitoring and Feedback. Open Microbiol J 13:279-285.

2. Al-Hamad A, Al-Ibrahim M, Alhajhouj E, Al-Alshaikh Jaffer W, Altowaileb J, Alfaraj H. 2016. Nurses’ competency in drawing blood cultures and educational intervention to reduce the contamination rate. J Infect Public Health 9:66-74.

3. Allen E, Cavallaro A, Keir AK. 2021. A Quality Improvement Initiative to Reduce Blood Culture Contamination in the Neonatal Unit. Pediatr Qual Saf 6:e413.

4. Bae M, In Kim H, Park JH, Ryu B-H, Chang J, Sung H, Jung J, Kim MJ, Kim S-H, Lee S-O, Choi S-H, Kim YS, Woo JH, Kim M-N, Chong YP. 2019. Improvement of blood culture contamination rate, blood volume, and true positive rate after introducing a dedicated phlebotomy team. Eur J Clin Microbiol Infect Dis 38:325-330.

5. Bell M, Bogar C, Plante J, Rasmussen K, Winters S. 2018. Effectiveness of a Novel Specimen Collection System in Reducing Blood Culture Contamination Rates. J Emerg Nurs 44:570-575.

6. El Feghaly RE, Chatterjee J, Dowdy K, Stempak LM, Morgan S, Needham W, Prystupa K, Kennedy M. 2018. A Quality Improvement Initiative: Reducing Blood Culture Contamination in a Children’s Hospital. Pediatrics 142.

7. Frota OP, Silva RM, Ruiz JS, Ferreira-Júnior MA, Hermann PRdS. 2022. Impact of sterile gloves on blood-culture contamination rates: A randomized clinical trial. Am J Infect Control 50:49-53.

8. Ge Y, Liu X-q, Xu Y-c, Xu S, Yu M-h, Zhang W, Deng G-h. 2011. Blood collection procedures influence contamination rates in blood culture: a prospective study. Chin Med J (Engl) 124:4002-4006.

9. Hall RT, Domenico HJ, Self WH, Hain PD. 2013. Reducing the Blood Culture Contamination Rate in a Pediatric Emergency Department and Subsequent Cost Savings. Pediatrics 131:e292-e297.

10. Halstead DC, Sautter RL, Snyder JW, Crist AE, Nachamkin I. 2020. Reducing Blood Culture Contamination Rates: Experiences of Four Hospital Systems. Infect Dis Ther 9:389-401.

11. Harding AD, Bollinger S. 2013. Reducing Blood Culture Contamination Rates in the Emergency Department. J Emerg Nurs 39:e1-e6.

12. He M, Huang S, Xiong J, Xiao Q. 2020. Improving adherence to facility protocol and reducing blood culture contamination in an intensive care unit: A quality improvement project. Aust Crit Care 33:546-552.

13. Kai M, Miyamoto K, Akamatsu K, Tsujita A, Nishio M. 2020. Effect of a bundle-approach intervention against contamination of blood culture in the emergency department. J Infect Chemother 26:785-789.

14. Kamboj M, Blair R, Bell N, Son C, Huang YT, Dowling M, Lipitz-Snyderman A, Eagan J, Sepkowitz K. 2015. Use of Disinfection Cap to Reduce Central-Line-Associated Bloodstream Infection and Blood Culture Contamination Among Hematology-Oncology Patients. Infect Control Hosp Epidemiol 36:1401-8.

15. Kim N-H, Kim M, Lee S, Yun NR, Kim K-H, Park SW, Kim HB, Kim N-J, Kim E-C, Park WB, Oh M-d. 2011. Effect of Routine Sterile Gloving on Contamination Rates in Blood Culture. Ann Intern Med 154:145-151.

16. Krajčinović SS, Doronjski A, Barišić N, Stojanović V. 2015. Risk Factors for Neonatal Sepsis and Method for Reduction of Blood Culture Contamination. Malawi Med J 27:20-4.

17. Lalezari A, Cohen MJ, Svinik O, Tel-Zur O, Sinvani S, Al-Dayem YA, Block C, Moses AE, Oster Y, Salameh S, Strahilevitz J. 2020. A simplified blood culture sampling protocol for reducing contamination and costs: a randomized controlled trial. Clin Microbiol Infect 26:470-474.

18. Levin PD, Moss J, Stohl S, Fried E, Cohen MJ, Sprung CL, Benenson S. 2013. Use of the nonwire central line hub to reduce blood culture contamination. Chest 143:640-645.

19. Lin C-M, Lee W-S, Lin F-Y, Yu F-L, Ou T-Y, Teng S-O. 2012. Reducing Blood Culture Contamination Rates by Educational Intervention and one-on-one Feedback in the Emergency Department. J Exp Clin Med 4:154-156.

20. Maeda N, Mori N, Shinjoh M, Komiyama O, Takahashi T. 2021. Comparison of 0.5% chlorhexidine gluconate alcohol with 10% povidone-iodine for skin disinfection in children to prevent blood culture contamination. J Infect Chemother 27:1027-1032.

21. Marini MA, Truog AW. 2013. Reducing false-positive peripheral blood cultures in a pediatric emergency department. J Emerg Nurs 39:440-6.

22. Marlowe L, Mistry RD, Coffin S, Leckerman KH, McGowan KL, Dai D, Bell LM, Zaoutis T. 2010. Blood culture contamination rates after skin antisepsis with chlorhexidine gluconate versus povidone-iodine in a pediatric emergency department. Infect Control Hosp Epidemiol 31:171-6.

23. Martínez J, Macías JH, Arreguín V, Álvarez JA, Macías AE, Mosqueda-Gómez JL. 2017. Isopropyl alcohol is as efficient as chlorhexidine to prevent contamination of blood cultures. Am J Infect Control 45:350-353.

24. McLaughlin LM, Inglis GD, Hoellering AB, Davies MW. 2013. Relationship between blood culture collection method and proportion of contaminated cultures in neonates. J Paediatr Child Health 49:105-8.

25. Moeller D. 2017. Eliminating Blood Culture False Positives: Harnessing the Power of Nursing Shared Governance. J Emerg Nurs 43:126-132.

26. Murillo TA, Beavers-May TK, English D, Plummer V, Stovall SH. 2011. Reducing contamination of peripheral blood cultures in a pediatric emergency department. Pediatr Emerg Care 27:918-21.

27. Nuntnarumit P, Sangsuksawang N. 2013. A randomized controlled trial of 1% aqueous chlorhexidine gluconate compared with 10% povidone-iodine for topical antiseptic in neonates: effects on blood culture contamination rates. Infect Control Hosp Epidemiol 34:430-2.

28. O'Connor C, Philip RK, Powell J, Slevin B, Quinn C, Power L, O'Connell NH, Dunne CP. 2016. Combined education and skin antisepsis intervention for persistently high blood-culture contamination rates in neonatal intensive care. J Hosp Infect 93:105-7.

29. Park WB, Myung SJ, Oh MD, Lee J, Kim NJ, Kim EC, Park JS. 2015. Educational intervention as an effective step for reducing blood culture contamination: a prospective cohort study. J Hosp Infect 91:111-6.

30. Patton RG, Schmitt T. 2010. Innovation for reducing blood culture contamination: initial specimen diversion technique. J Clin Microbiol 48:4501-3.

31. Ramirez P, Gordón M, Cortes C, Villarreal E, Perez-Belles C, Robles C, de Hevia L, Marti JV, Botella J, Bonastre J. 2015. Blood culture contamination rate in an intensive care setting: Effectiveness of an education-based intervention. Am J Infect Control 43:844-847.

32. Roth A, Wiklund AE, Pålsson AS, Melander EZ, Wullt M, Cronqvist J, Walder M, Sturegård E. 2010. Reducing blood culture contamination by a simple informational intervention. J Clin Microbiol 48:4552-8.

33. Rupp ME, Cavalieri RJ, Marolf C, Lyden E. 2017. Reduction in Blood Culture Contamination Through Use of Initial Specimen Diversion Device. Clin Infect Dis 65:201-205.

34. Ryan C. 2017. Implementation of the Theory of Planned Behavior to Promote Compliance with a Chlorhexidine Gluconate Protocol. Journal of the Association for Vascular Access 22:64-70.

35. Sánchez-Sánchez MM, Arias-Rivera S, Fraile-Gamo P, Jareño-Collado R, López-Román S, Vadillo-Obesso P, García-González S, Pulido-Martos MT, Sánchez-Muñoz EI, Cacho-Calvo J, Martín-Pellicer A, Panadero-del Olmo L, Frutos-Vivar F. 2018. Efecto de una acción formativa en cuidados intensivos sobre la tasa de contaminación de hemocultivos. Enferm Intensiva (Engl Ed) 29:121-127.

36. Santos CAQ, Shimasaki T, Kishen E, Won S, Hanson A, Marinakos G, Tomich A, Hota B, Segreti J. 2018. Impact of Phlebotomist-Only Venipuncture and Central Line Avoidance for Blood Culture in a Large Tertiary Care University Hospital. Infect Dis Clin Pract (Baltim Md) 26:91-96.

37. Self WH, Mickanin J, Grijalva CG, Grant FH, Henderson MC, Corley G, Blaschke Ii DG, McNaughton CD, Barrett TW, Talbot TR, Paul BR. 2014. Reducing blood culture contamination in community hospital emergency departments: a multicenter evaluation of a quality improvement intervention. Acad Emerg Med 21:274-82.

38. Self WH, Speroff T, Grijalva CG, McNaughton CD, Ashburn J, Liu D, Arbogast PG, Russ S, Storrow AB, Talbot TR. 2013. Reducing blood culture contamination in the emergency department: an interrupted time series quality improvement study. Acad Emerg Med 20:89-97.

39. Shaheen N, Zeeshan M, Fasih N, Farooqi J, Jabeen K, Irfan S. 2020. Efforts to improve diagnosis of bacteraemia by reducing blood culture contamination in an emergency department: strategies and outcome. J Pak Med Assoc 70:835-839.

40. Story-Roller E, Weinstein MP. 2016. Chlorhexidine versus Tincture of Iodine for Reduction of Blood Culture Contamination Rates: a Prospective Randomized Crossover Study. Journal of clinical microbiology 54:3007‐3009.

41. Sweet MA, Cumpston A, Briggs F, Craig M, Hamadani M. 2012. Impact of alcohol-impregnated port protectors and needleless neutral pressure connectors on central line-associated bloodstream infections and contamination of blood cultures in an inpatient oncology unit. Am J Infect Control 40:931-934.

42. Syed S, Liss DT, Costas CO, Atkinson JM. 2020. Diversion Principle Reduces Skin Flora Contamination Rates in a Community Hospital. Arch Pathol Lab Med 144:215-220.

43. Tangsathapompong A, Banjongmanee P, Unrit K, Sritipsukho P, Mungkornkaew N, Sajak S. 2014. The efficacy of 2% chlorhexidine gluconate in 70% alcohol compared with 10% povidone iodine in reducing blood culture contamination in pediatric patients. J Med Assoc Thai 97 Suppl 8:S34-40.

44. Tarai B, Das P, Kumar D, Budhiraja S. 2012. Comparative evaluation of paired blood culture (aerobic/aerobic) and single blood culture, along with clinical importance in catheter versus peripheral line at a tertiary care hospital. Indian J Med Microbiol 30:187-92.

45. Tarrand JJ, LaSala PR, Han X-Y, Rolston KV, Kontoyiannis DP. 2012. Dimethyl Sulfoxide Enhances Effectiveness of Skin Antiseptics and Reduces Contamination Rates of Blood Cultures. J Clin Microbiol 50:1552-1557.

46. Thomas S, Cheesbrough J, Plumb S, Bolton L, Wilkinson P, Walmsley J, Diggle P. 2011. Impact of a blood culture collection kit on the quality of blood culture sampling: fear and the law of unintended consequences. J Hosp Infect 78:256-259.

47. Yodoshi T, Ueda S, Goldman RD. 2019. Skin preparation for prevention of peripheral blood culture contamination in children. Pediatr Int 61:647-651.

48. Yu D, Larsson A, Parke Å, Unge C, Henning C, Sundén-Cullberg J, Somell A, Strålin K, Özenci V. 2020. Single-Sampling Strategy vs. Multi-Sampling Strategy for Blood Cultures in Sepsis: A Prospective Non-inferiority Study. Front Microbiol 11.

49. Zimmerman FS, Karameh H, Ben-Chetrit E, Zalut T, Assous M, Levin PD. 2019. Modification of Blood Test Draw Order to Reduce Blood Culture Contamination: A Randomized Clinical Trial. Clin Infect Dis 71:1215-1220.

50. Story-Roller E, Weinstein MP. 2016. Chlorhexidine versus Tincture of Iodine for Reduction of Blood Culture Contamination Rates: a Prospective Randomized Crossover Study. J Clin Microbiol 54:3007-3009.
