# Supplementary material for: American Society for Microbiology evidence-based laboratory medicine practice guidelines to reduce blood culture contamination rates: a systematic review and meta-analysis
Source: Clin Microbiol Rev. 2024 Nov 4;37(4):e00087-24. doi: 10.1128/cmr.00087-24 (PMC11629619; doi:10.1128/cmr.00087-24)
Supplement: Supplemental Tables S1 to S4 — Table S1 (GRADE evidence summary table), Table S2 (change between baseline and follow-up blood culture contamination rates by unit), Table S3 (outlier sensitivity analysis by unit), and Table S4 (study detail on outlying over- versus underperforming interventions). [file cmr.00087-24-s0002.docx]

Table S1. GRADE Evidence Summary Table

| **Certainty assessment** | | | | | | | **№ of patients** | | **Effect** | | | **Certainty** | | **Importance** |
| --- | --- | --- | --- | --- | --- | --- | --- | --- | --- | --- | --- | --- | --- | --- |
| **№ of studies** | **Study design** | **Risk of bias** | **Inconsistency** | **Indirectness** | **Imprecision** | **Other considerations** | **comprehensive process improvement efforts** | **standard practice** | **Relative** **(95% CI)** | **Absolute** **(95% CI)** |  | |  |  |
| Blood Culture Contamination: Cohort Designs | | | | | | | | | | | | | | |
| 11 | observational studies | serious^a,b,c^ | serious^d^ | not serious | serious^e^ | dose response gradient | 1341/92279 (1.5%) | 1815/106726 (1.7%) | **RR 0.83** (0.49 to 1.40) | | **3 fewer per 1,000** (from 9 fewer to 7 more) | ⨁◯◯◯ Very low | | CRITICAL |
| Blood Culture Contamination: Before/After Designs | | | | | | | | | | | | | | |
| 35 | observational studies | not serious | not serious | not serious | not serious | strong association dose response gradient | 4157/316902 (1.3%) | 11200/421385 (2.7%) | **RR 0.45** (0.39 to 0.53) | | **15 fewer per 1,000** (from 16 fewer to 12 fewer) | ⨁⨁⨁⨁ High | | CRITICAL |
| Blood Culture Contamination: Controlled Trials | | | | | | | | | | | | | | |
| 15 | randomised trials | serious^c,f^ | not serious | not serious | serious^e^ | strong association dose response gradient | 509/24746 (2.1%) | 633/22504 (2.8%) | **RR 0.49** (0.33 to 0.73) | | **14 fewer per 1,000** (from 19 fewer to 8 fewer) | ⨁⨁⨁⨁ High | | CRITICAL |

Using the GRADE criteria to evaluate the strength of evidence (Schunemann 2013) the team concluded that there was high confidence in the findings across two of the study design types (before/after and controlled trial designs) holding all else equal

Table S2. Change between Baseline and Follow-Up Blood Culture Contamination Rates by Unit

| Author | year | Baseline BCC Rate | Follow-up BCC Rate | Change | Unweighted Unit Average Change |
| --- | --- | --- | --- | --- | --- |
| ED |  |  |  |  | -1.6% |
| Al-Hamad | 2019 | 7.7% | 2.3% | -5.4% |  |
| Kai | 2020 | 6.3% | 1.1% | -5.2% |  |
| Shaheen | 2020 | 8.0% | 3.9% | -4.1% |  |
| Moeller | 2017 | 5.4% | 1.8% | -3.6% |  |
| Zimmerman | 2019 | 5.4% | 2.2% | -3.3% |  |
| Self | 2012 | 6.5% | 3.6% | -3.0% |  |
| Bell | 2018 | 3.5% | 0.6% | -2.9% |  |
| Self | 2013 | 4.3% | 1.7% | -2.7% |  |
| Self1 | 2014 | 4.8% | 2.7% | -2.1% |  |
| Yu | 2020 | 7.3% | 5.3% | -2.0% |  |
| Self2 | 2014 | 2.5% | 0.9% | -1.6% |  |
| Rupp | 2017 | 1.8% | 0.2% | -1.5% |  |
| Lin | 2012 | 3.4% | 2.0% | -1.4% |  |
| Ryan | 2017 | 3.1% | 1.8% | -1.3% |  |
| Syed | 2020 | 2.9% | 2.0% | -0.9% |  |
| Harding | 2013 | 1.8% | 1.0% | -0.8% |  |
| Kelly | 2013 | 3.6% | 3.2% | -0.3% |  |
| Murillo | 2011 | 5.0% | 4.9% | -0.1% |  |
| Martínez | 2017 | 0.5% | 2.0% | 1.5% |  |
| Al-Hamad | 2016 | 7.6% | 16.3% | 8.6% |  |
| General Units | |  |  |  | -0.01 |
| Thomas | 2011 | 9.2% | 4.1% | -5.1% |  |
| Lalezari | 2020 | 5.0% | 1.7% | -3.3% |  |
| Al-Hamad | 2016 | 8.2% | 5.2% | -3.0% |  |
| Ge | 2011 | 1.3% | 0.2% | -1.1% |  |
| HalsteadC | 2020 | 1.9% | 1.4% | -0.6% |  |
| Syed | 2020 | 1.8% | 1.3% | -0.5% |  |
| Kim | 2011 | 1.2% | 0.8% | -0.4% |  |
| Bae | 2019 | 0.3% | 0.2% | -0.1% |  |
| Martínez | 2017 | 1.4% | 2.4% | 1.0% |  |
| ICU/Heme/Onc | |  |  |  | -4.00% |
| Levin | 2013 | 19.1% | 5.2% | -13.9% |  |
| Ramirez | 2015 | 23.0% | 13.0% | -10.0% |  |
| Frota | 2021 | 10.0% | 1.0% | -9.0% |  |
| Sanchez | 2018 | 13.9% | 5.6% | -8.3% |  |
| Al-Hamad | 2016 | 13.4% | 7.4% | -6.0% |  |
| He | 2020 | 4.5% | 0.6% | -3.9% |  |
| Sweet | 2012 | 2.5% | 0.2% | -2.2% |  |
| Kamboj | 2015 | 3.2% | 1.2% | -2.0% |  |
| Kim | 2011 | 1.0% | 0.4% | -0.6% |  |
| Bae | 2019 | 0.6% | 0.3% | -0.3% |  |
| Martínez | 2017 | 0.0% | 0.0% | 0.0% |  |
| Scheinemann | 2010 | 14.2% | 14.2% | 0.0% |  |
| Stohl | 2011 | 3.7% | 8.2% | 4.6% |  |
| Pedes Units | |  |  |  | -2.1% |
| Krajčinović | 2015 | 16.4% | 7.6% | -8.8% |  |
| O'Connor | 2016 | 4.7% | 1.0% | -3.7% |  |
| Nuntnarumit | 2013 | 2.9% | 0.0% | -2.9% |  |
| Al-Hamad | 2016 | 7.1% | 4.5% | -2.6% |  |
| Berger | 2018 | 4.0% | 1.4% | -2.5% |  |
| Hall | 2013 | 3.9% | 1.6% | -2.3% |  |
| Maeda | 2021 | 2.3% | 0.3% | -2.0% |  |
| El Feghaly | 2018 | 2.8% | 1.5% | -1.3% |  |
| Allen | 2021 | 2.0% | 0.9% | -1.1% |  |
| Marlowe | 2010 | 2.5% | 1.7% | -0.8% |  |
| Marini | 2013 | 2.1% | 1.4% | -0.7% |  |
| Yodoshi | 2019 | 2.2% | 1.6% | -0.6% |  |
| McLaughlin | 2013 | 2.3% | 3.9% | 1.6% |  |
| Combined Units | |  |  |  | -1.2% |
| HalsteadD | 2020 | 10.3% | 2.0% | -8.3% |  |
| Al-Hamad | 2016 | 8.6% | 5.6% | -3.0% |  |
| HalsteadA | 2020 | 3.8% | 1.0% | -2.8% |  |
| Tarrand | 2012 | 3.0% | 0.9% | -2.1% |  |
| HalsteadB | 2020 | 2.3% | 0.9% | -1.4% |  |
| Tangsathapompong | 2014 | 3.2% | 2.3% | -0.9% |  |
| Syed | 2020 | 2.4% | 1.7% | -0.7% |  |
| Kim | 2011 | 1.1% | 0.6% | -0.5% |  |
| Roth | 2010 | 2.6% | 2.2% | -0.4% |  |
| Santos | 2018 | 1.2% | 0.9% | -0.4% |  |
| Park | 2015 | 1.4% | 1.1% | -0.3% |  |
| Bae | 2019 | 0.4% | 0.3% | -0.2% |  |
| Story-Roller | 2016 | 3.9% | 3.9% | 0.1% |  |
| Martínez | 2017 | 0.9% | 1.9% | 1.0% |  |
| Tarai | 2012 | 1.1% | 3.3% | 2.2% |  |

To put the risk ratios in context, Supplemental Table S2 provides the baseline and follow-up BCC rates as well as the change from baseline to follow-up by unit.

Table S3. Outlier Sensitivity Analysis by Unit

The table below presents the risk ratios of the effect of the intervention when high and low outlying studies are dropped from the analysis. These values may be interpreted as a “typical effect” when the effects of very low performing or very high performing interventions are excluded from the analysis.

|  | N Studies | N samples | RR_a_ | RR LCI | RR UCI | I^2^ | I^2^ LCI | I^2^ UCI | p-value of RR | Studies Removed | P-value of I^2^ |
| --- | --- | --- | --- | --- | --- | --- | --- | --- | --- | --- | --- |
| ED | 15 | 150857 | 0.47 | 0.38 | 0.59 | 68.4 | 46.0 | 81.5 | < 0.0001 | "Al-Hamad 2016", "Bell 2018", "Murillo 2011" | 0.0001 |
| General Units | 8 | 157363 | 0.65 | 0.55 | 0.78 | 29.3 | 0 | 68.4 | < 0.0001 | "Ge 2011" | 0.194 |
| ICU/Heme/Onc | 9 | 104240 | 0.41 | 0.32 | 0.54 | 44.9 | 0 | 74.5 | < 0.0001 | "He 2020" | <0.069 |
| Peds Units | 11 | 243066 | 0.54 | 0.44 | 0.67 | 61.3 | 25.4 | 80.0 | < 0.0001 | "McLaughlin 2013" | 0.0039 |
| Combined Units | 12 | 512745 | 0.72 | 0.63 | 0.84 | 67 | 39.5 | 82 | < 0.0001 | "HalsteadA 2020", "HalsteadB 2020", "HalsteadD 2020", "Tarai 2012" | 0.0005 |

A sensitivity analysis was performed with outlying studies omitted from the analyses. Interventions could be either “overperforming” (i.e., where the lower 95% confidence interval of the study effect was above the upper 95% confidence interval of the pooled effect) or “underperforming” (i.e., the upper 95% confidence interval of the study effect was below the lower 95% confidence interval of the pooled effect).

Table S4. Study Detail on Outlying Over- Versus Underperforming Interventions

| **Unit and Status** | **Studies** | **Comments** |
| --- | --- | --- |
| **ED** |  |  |
| Overperforming Interventions | Bell 2018 | **Process Improvement:** Initial specimen diversion device (ISDD) versus Standard (?).  **QMQI**: Yes  **Behavioral**: Education, Training (specific to device)  **Focus**: Device focused  **Blood Collection Method:** Single venipuncture  **Skin Disinfection**: alcohol pads and a ChloraPrep scrub was used on the skin surface for 30-60 seconds  **Collectors**: Nurses  **Location**: USA  **Who draws labs**: |
| Underperforming Interventions | Al-Hamad 2016 | **Process Improvement:** Centralized educational workshops versus on unit instruction.  **QMQI**: Yes  **Behavioral**: Education, Training and Feedback (across the entire collection process)  **Focus**: Education focused  **Blood Collection Method:** Single venipuncture  **Skin Disinfection**: 70% iso-propyl alcohol, in concentric circles beginning at the center of the site, followed by 10% povidone-iodine, again in concentric circles; then, it allowed to air dry for 1.5—2 min before venesection  **Collectors**: Nurses  **Location**: Saudi Arabia |
|  | Murillo 2011 |  |
| **General Units** |  |  |
| Overperforming Interventions | Ge 2011 |  |
| **ICU/Heme/Onc** |  |  |
| Overperforming Interventions | He 2020 |  |
| Underperforming Interventions | Stohl 2011 |  |
| **Pedes Units** |  |  |
| Underperforming Interventions | McLaughlin 2013 |  |
| **Combined Units** |  |  |
| Overperforming Interventions | HalsteadA 2020 |  |
|  | HalsteadB 2020 |  |
|  | HalsteadD 2020 |  |
| Underperforming Interventions | Tarai 2012 |  |

Heterogeneity across analyses remained high--indicating that differences in local efforts can have a large effect on the success of an implementation effort. Additionally, as is evident from Supplemental Table S4, context matters—similar efforts in different types of units may not perform equally well—some efforts “overperformed” the average effects while some “underperformed.” The key to understanding the analyses above is not to expect a precise level of improvement, but to identify key features that are likely to lead to greater success reducing BCC rates.

# References

1. Al-Hamad A. 2019. Successful Reduction of Blood Culture Contamination in an Emergency Department by Monitoring and Feedback. Open Microbiol J 13:279-285.

2. Al-Hamad A, Al-Ibrahim M, Alhajhouj E, Al-Alshaikh Jaffer W, Altowaileb J, Alfaraj H. 2016. Nurses’ competency in drawing blood cultures and educational intervention to reduce the contamination rate. J Infect Public Health 9:66-74.

3. Allen E, Cavallaro A, Keir AK. 2021. A Quality Improvement Initiative to Reduce Blood Culture Contamination in the Neonatal Unit. Pediatr Qual Saf 6:e413.

4. Bae M, In Kim H, Park JH, Ryu B-H, Chang J, Sung H, Jung J, Kim MJ, Kim S-H, Lee S-O, Choi S-H, Kim YS, Woo JH, Kim M-N, Chong YP. 2019. Improvement of blood culture contamination rate, blood volume, and true positive rate after introducing a dedicated phlebotomy team. Eur J Clin Microbiol Infect Dis 38:325-330.

5. Bell M, Bogar C, Plante J, Rasmussen K, Winters S. 2018. Effectiveness of a Novel Specimen Collection System in Reducing Blood Culture Contamination Rates. J Emerg Nurs 44:570-575.

6. Berger I, Gil Margolis M, Nahum E, Dagan O, Levy I, Kaplan E, Shostak E, Shmuelov E, Schiller O, Kadmon G. 2018. Blood Cultures Drawn From Arterial Catheters Are Reliable for the Detection of Bloodstream Infection in Critically Ill Children. Pediatric Critical Care Medicine 19:e213-e218.

7. El Feghaly RE, Chatterjee J, Dowdy K, Stempak LM, Morgan S, Needham W, Prystupa K, Kennedy M. 2018. A Quality Improvement Initiative: Reducing Blood Culture Contamination in a Children’s Hospital. Pediatrics 142.

8. Frota OP, Silva RM, Ruiz JS, Ferreira-Júnior MA, Hermann PRdS. 2022. Impact of sterile gloves on blood-culture contamination rates: A randomized clinical trial. Am J Infect Control 50:49-53.

9. Ge Y, Liu X-q, Xu Y-c, Xu S, Yu M-h, Zhang W, Deng G-h. 2011. Blood collection procedures influence contamination rates in blood culture: a prospective study. Chin Med J (Engl) 124:4002-4006.

10. Hall RT, Domenico HJ, Self WH, Hain PD. 2013. Reducing the Blood Culture Contamination Rate in a Pediatric Emergency Department and Subsequent Cost Savings. Pediatrics 131:e292-e297.

11. Halstead DC, Sautter RL, Snyder JW, Crist AE, Nachamkin I. 2020. Reducing Blood Culture Contamination Rates: Experiences of Four Hospital Systems. Infect Dis Ther 9:389-401.

12. Harding AD, Bollinger S. 2013. Reducing Blood Culture Contamination Rates in the Emergency Department. J Emerg Nurs 39:e1-e6.

13. He M, Huang S, Xiong J, Xiao Q. 2020. Improving adherence to facility protocol and reducing blood culture contamination in an intensive care unit: A quality improvement project. Aust Crit Care 33:546-552.

14. Kai M, Miyamoto K, Akamatsu K, Tsujita A, Nishio M. 2020. Effect of a bundle-approach intervention against contamination of blood culture in the emergency department. J Infect Chemother 26:785-789.

15. Kamboj M, Blair R, Bell N, Son C, Huang YT, Dowling M, Lipitz-Snyderman A, Eagan J, Sepkowitz K. 2015. Use of Disinfection Cap to Reduce Central-Line-Associated Bloodstream Infection and Blood Culture Contamination Among Hematology-Oncology Patients. Infect Control Hosp Epidemiol 36:1401-8.

16. Kelly A-M, Klim S. 2013. Taking blood cultures from a newly established intravenous catheter in the emergency department does not increase the rate of contaminated blood cultures. Emergency Medicine Australasia 25:435-438.

17. Kim N-H, Kim M, Lee S, Yun NR, Kim K-H, Park SW, Kim HB, Kim N-J, Kim E-C, Park WB, Oh M-d. 2011. Effect of Routine Sterile Gloving on Contamination Rates in Blood Culture. Ann Intern Med 154:145-151.

18. Krajčinović SS, Doronjski A, Barišić N, Stojanović V. 2015. Risk Factors for Neonatal Sepsis and Method for Reduction of Blood Culture Contamination. Malawi Med J 27:20-4.

19. Lalezari A, Cohen MJ, Svinik O, Tel-Zur O, Sinvani S, Al-Dayem YA, Block C, Moses AE, Oster Y, Salameh S, Strahilevitz J. 2020. A simplified blood culture sampling protocol for reducing contamination and costs: a randomized controlled trial. Clin Microbiol Infect 26:470-474.

20. Levin PD, Moss J, Stohl S, Fried E, Cohen MJ, Sprung CL, Benenson S. 2013. Use of the nonwire central line hub to reduce blood culture contamination. Chest 143:640-645.

21. Lin C-M, Lee W-S, Lin F-Y, Yu F-L, Ou T-Y, Teng S-O. 2012. Reducing Blood Culture Contamination Rates by Educational Intervention and one-on-one Feedback in the Emergency Department. J Exp Clin Med 4:154-156.

22. Maeda N, Mori N, Shinjoh M, Komiyama O, Takahashi T. 2021. Comparison of 0.5% chlorhexidine gluconate alcohol with 10% povidone-iodine for skin disinfection in children to prevent blood culture contamination. J Infect Chemother 27:1027-1032.

23. Marini MA, Truog AW. 2013. Reducing false-positive peripheral blood cultures in a pediatric emergency department. J Emerg Nurs 39:440-6.

24. Marlowe L, Mistry RD, Coffin S, Leckerman KH, McGowan KL, Dai D, Bell LM, Zaoutis T. 2010. Blood culture contamination rates after skin antisepsis with chlorhexidine gluconate versus povidone-iodine in a pediatric emergency department. Infect Control Hosp Epidemiol 31:171-6.

25. Martínez J, Macías JH, Arreguín V, Álvarez JA, Macías AE, Mosqueda-Gómez JL. 2017. Isopropyl alcohol is as efficient as chlorhexidine to prevent contamination of blood cultures. Am J Infect Control 45:350-353.

26. McLaughlin LM, Inglis GD, Hoellering AB, Davies MW. 2013. Relationship between blood culture collection method and proportion of contaminated cultures in neonates. J Paediatr Child Health 49:105-8.

27. Moeller D. 2017. Eliminating Blood Culture False Positives: Harnessing the Power of Nursing Shared Governance. J Emerg Nurs 43:126-132.

28. Murillo TA, Beavers-May TK, English D, Plummer V, Stovall SH. 2011. Reducing contamination of peripheral blood cultures in a pediatric emergency department. Pediatr Emerg Care 27:918-21.

29. Nuntnarumit P, Sangsuksawang N. 2013. A randomized controlled trial of 1% aqueous chlorhexidine gluconate compared with 10% povidone-iodine for topical antiseptic in neonates: effects on blood culture contamination rates. Infect Control Hosp Epidemiol 34:430-2.

30. O'Connor C, Philip RK, Powell J, Slevin B, Quinn C, Power L, O'Connell NH, Dunne CP. 2016. Combined education and skin antisepsis intervention for persistently high blood-culture contamination rates in neonatal intensive care. J Hosp Infect 93:105-7.

31. Park WB, Myung SJ, Oh MD, Lee J, Kim NJ, Kim EC, Park JS. 2015. Educational intervention as an effective step for reducing blood culture contamination: a prospective cohort study. J Hosp Infect 91:111-6.

32. Ramirez P, Gordón M, Cortes C, Villarreal E, Perez-Belles C, Robles C, de Hevia L, Marti JV, Botella J, Bonastre J. 2015. Blood culture contamination rate in an intensive care setting: Effectiveness of an education-based intervention. Am J Infect Control 43:844-847.

33. Roth A, Wiklund AE, Pålsson AS, Melander EZ, Wullt M, Cronqvist J, Walder M, Sturegård E. 2010. Reducing blood culture contamination by a simple informational intervention. J Clin Microbiol 48:4552-8.

34. Rupp ME, Cavalieri RJ, Marolf C, Lyden E. 2017. Reduction in Blood Culture Contamination Through Use of Initial Specimen Diversion Device. Clin Infect Dis 65:201-205.

35. Ryan C. 2017. Implementation of the Theory of Planned Behavior to Promote Compliance with a Chlorhexidine Gluconate Protocol. Journal of the Association for Vascular Access 22:64-70.

36. Sánchez-Sánchez MM, Arias-Rivera S, Fraile-Gamo P, Jareño-Collado R, López-Román S, Vadillo-Obesso P, García-González S, Pulido-Martos MT, Sánchez-Muñoz EI, Cacho-Calvo J, Martín-Pellicer A, Panadero-del Olmo L, Frutos-Vivar F. 2018. Efecto de una acción formativa en cuidados intensivos sobre la tasa de contaminación de hemocultivos. Enferm Intensiva (Engl Ed) 29:121-127.

37. Santos CAQ, Shimasaki T, Kishen E, Won S, Hanson A, Marinakos G, Tomich A, Hota B, Segreti J. 2018. Impact of Phlebotomist-Only Venipuncture and Central Line Avoidance for Blood Culture in a Large Tertiary Care University Hospital. Infect Dis Clin Pract (Baltim Md) 26:91-96.

38. Scheinemann K, Ethier MC, Dupuis LL, Richardson SE, Doyle J, Allen U, Sung L. 2010. Utility of peripheral blood cultures in bacteremic pediatric cancer patients with a central line. Support Care Cancer 18:913-9.

39. Self WH, Mickanin J, Grijalva CG, Grant FH, Henderson MC, Corley G, Blaschke Ii DG, McNaughton CD, Barrett TW, Talbot TR, Paul BR. 2014. Reducing blood culture contamination in community hospital emergency departments: a multicenter evaluation of a quality improvement intervention. Acad Emerg Med 21:274-82.

40. Self WH, Speroff T, Grijalva CG, McNaughton CD, Ashburn J, Liu D, Arbogast PG, Russ S, Storrow AB, Talbot TR. 2013. Reducing blood culture contamination in the emergency department: an interrupted time series quality improvement study. Acad Emerg Med 20:89-97.

41. Self WH, Speroff T, McNaughton CD, Wright PW, Miller G, Johnson JG, Daniels TL, Talbot TR. 2012. Blood culture collection through peripheral intravenous catheters increases the risk of specimen contamination among adult emergency department patients. Infect Control Hosp Epidemiol 33:524-6.

42. Shaheen N, Zeeshan M, Fasih N, Farooqi J, Jabeen K, Irfan S. 2020. Efforts to improve diagnosis of bacteraemia by reducing blood culture contamination in an emergency department: strategies and outcome. J Pak Med Assoc 70:835-839.

43. Stohl S, Benenson S, Sviri S, Avidan A, Block C, Sprung CL, Levin PD. 2011. Blood Cultures at Central Line Insertion in the Intensive Care Unit: Comparison with Peripheral Venipuncture. J Clin Microbiol 49:2398-2403.

44. Story-Roller E, Weinstein MP. 2016. Chlorhexidine versus Tincture of Iodine for Reduction of Blood Culture Contamination Rates: a Prospective Randomized Crossover Study. J Clin Microbiol 54:3007-3009.

45. Sweet MA, Cumpston A, Briggs F, Craig M, Hamadani M. 2012. Impact of alcohol-impregnated port protectors and needleless neutral pressure connectors on central line-associated bloodstream infections and contamination of blood cultures in an inpatient oncology unit. Am J Infect Control 40:931-934.

46. Syed S, Liss DT, Costas CO, Atkinson JM. 2020. Diversion Principle Reduces Skin Flora Contamination Rates in a Community Hospital. Arch Pathol Lab Med 144:215-220.

47. Tangsathapompong A, Banjongmanee P, Unrit K, Sritipsukho P, Mungkornkaew N, Sajak S. 2014. The efficacy of 2% chlorhexidine gluconate in 70% alcohol compared with 10% povidone iodine in reducing blood culture contamination in pediatric patients. J Med Assoc Thai 97 Suppl 8:S34-40.

48. Tarai B, Das P, Kumar D, Budhiraja S. 2012. Comparative evaluation of paired blood culture (aerobic/aerobic) and single blood culture, along with clinical importance in catheter versus peripheral line at a tertiary care hospital. Indian J Med Microbiol 30:187-92.

49. Tarrand JJ, LaSala PR, Han X-Y, Rolston KV, Kontoyiannis DP. 2012. Dimethyl Sulfoxide Enhances Effectiveness of Skin Antiseptics and Reduces Contamination Rates of Blood Cultures. J Clin Microbiol 50:1552-1557.

50. Thomas S, Cheesbrough J, Plumb S, Bolton L, Wilkinson P, Walmsley J, Diggle P. 2011. Impact of a blood culture collection kit on the quality of blood culture sampling: fear and the law of unintended consequences. J Hosp Infect 78:256-259.

51. Yodoshi T, Ueda S, Goldman RD. 2019. Skin preparation for prevention of peripheral blood culture contamination in children. Pediatr Int 61:647-651.

52. Yu D, Larsson A, Parke Å, Unge C, Henning C, Sundén-Cullberg J, Somell A, Strålin K, Özenci V. 2020. Single-Sampling Strategy vs. Multi-Sampling Strategy for Blood Cultures in Sepsis: A Prospective Non-inferiority Study. Front Microbiol 11.

53. Zimmerman FS, Karameh H, Ben-Chetrit E, Zalut T, Assous M, Levin PD. 2019. Modification of Blood Test Draw Order to Reduce Blood Culture Contamination: A Randomized Clinical Trial. Clin Infect Dis 71:1215-1220.
