# Supplementary material for: American Society for Microbiology evidence-based laboratory medicine practice guidelines to reduce blood culture contamination rates: a systematic review and meta-analysis
Source: Clin Microbiol Rev. 2024 Nov 4;37(4):e00087-24. doi: 10.1128/cmr.00087-24 (PMC11629619; doi:10.1128/cmr.00087-24)

Blood Culture Contamination Guidelines Documentation

ASM/RU Evidence-Based Laboratory Guidelines Committee

Systematic Review & Meta-Analysis Update 2022

This document contains details the search history, search strategies, records identification, records screening, methodology, and Prisma figure.

# Search History

## Initial Searches

Date Ran: 10/2/2017

Date Range: 1995 – 10/2/2017

Run By: NIH Librarian

Limits: English

## Updated Searches

### Update #1

Date Ran: 2/20/2018

Date Range: 10/2/2017 – 2/20/2018

Run By: NIH Librarian

Limits: English

### Update #2

Date Ran: 12/6/2018

Date Range: 2/20/2018 – 12/6/2018

Run By: NIH Librarian

Limits: English

### Update #3

Date Ran: 9/29/2021

Date Range: 12/1/2018 – 9/29/2021

Run By: Lindsay Boyce (Research Informationist, MSKCC)

Limits: English

# Search Strategies

## Medline (Ovid)

((Blood ADJ2 sampl*) OR (Blood ADJ2 culture*) OR (Blood ADJ2 specimen*) OR (Blood ADJ2 collect*) OR (Blood ADJ2 draw*) OR (Blood ADJ2 test*))

**AND**

(Phlebotomy/mt OR Venipuncture OR venapuncture OR venepuncture OR venupuncture OR vein puncture* OR venesection OR (venous ADJ2 catheter*) OR (venous ADJ2 sampl*) OR (venous ADJ2 puncture*) OR (intravenous ADJ2 catheter*) OR (indwelling ADJ2 catheter*) OR (vein* ADJ2 catheter*) OR (catheter* ADJ2 draw*) OR (catheter* ADJ2 culture*) OR (catheter* ADJ2 collect*) OR (peripheral ADJ2 culture*) OR (peripheral ADJ2 catheter*) OR phlebotomy team* OR phlebotomist* OR (phlebotom* ADJ2 method*) OR (phlebotom* ADJ2 practice*) OR (prep* ADJ2 kit*) OR (collection ADJ2 kit*) OR antisepsis OR antiseptic OR antiinfective* OR anti-infective* OR disinfect* OR decontamina* OR chloraprep OR (skin ADJ2 prep*) OR (patient* ADJ2 prep*))

**AND**

(contamina* OR false positive* OR false negative* OR clinical utility OR clinical use* OR predictive value* OR specificity OR sensitivity OR error* OR analytic* OR pre-analytic* OR preanalytic* OR quality OR cost* OR harm* OR recommend* OR guideline*)

## Embase (Ovid/Elsevier)

((Blood NEAR/2 sampl*) OR (Blood NEAR/2 culture*) OR (Blood NEAR/2 specimen*) OR (Blood NEAR/2 collect*) OR (Blood NEAR/2 draw*) OR (Blood NEAR/2 test*))

**AND**

(Phlebotomy/de OR Venipuncture OR venapuncture OR venepuncture OR venupuncture OR "vein puncture*" OR venesection OR (venous NEAR/2 catheter*) OR (venous NEAR/2 sampl*) OR (venous NEAR/2 puncture*) OR (intravenous NEAR/2 catheter*) OR (indwelling NEAR/2 catheter*) OR (vein* NEAR/2 catheter*) OR (catheter* NEAR/2 draw*) OR (catheter* NEAR/2 culture*) OR (catheter* NEAR/2 collect*) OR (peripheral NEAR/2 culture*) OR (peripheral NEAR/2 catheter*) OR "phlebotomy team*" OR phlebotomist* OR (phlebotom* NEAR/2 method*) OR (phlebotom* NEAR/2 practice*) OR (prep* NEAR/2 kit*) OR (collection NEAR/2 kit*) OR antisepsis OR antiseptic OR antiinfective* OR anti-infective* OR disinfect* OR decontamina* OR chloraprep OR (skin NEAR/2 prep*) OR (patient* NEAR/2 prep*))

**AND**

(contamina* OR "false positive*" OR "false negative*" OR "clinical utility" OR "clinical use*" OR "predictive value*" OR Specificity OR Sensitivity OR error* OR analytic* OR pre-analytic* OR preanalytic* OR Quality OR cost* OR harm* OR recommend* OR guideline*)

## CINAHL (EBSCO)

((Blood N2 sampl*) OR (Blood N2 culture*) OR (Blood N2 specimen*) OR (Blood N2 collect*) OR (Blood N2 draw*) OR (Blood N2 test*))

**AND**

((MH Phlebotomy/MT) OR Venipuncture OR venapuncture OR venepuncture OR venupuncture OR venesection OR (venous N2 catheter*) OR (venous N2 sampl*) OR (venous N2 puncture*) OR (intravenous N2 catheter*) OR (indwelling N2 catheter*) OR (vein* N2 catheter*) OR (catheter* N2 draw*) OR (catheter* N2 culture*) OR (catheter* N2 collect*) OR (peripheral N2 culture*) OR (peripheral N2 catheter*) OR phlebotomy team* OR phlebotomist* OR (phlebotom* N2 method*) OR (phlebotom* N2 practice*) OR (prep* N2 kit*) OR (collection N2 kit*) OR antisepsis OR antiseptic OR antiinfective* OR anti-infective* OR disinfect* OR decontamina* OR chloraprep OR (skin N2 prep*) OR (patient* N2 prep*))

**AND**

(contamina* OR “false positive*” OR “false negative*” OR “clinical utility” OR “clinical use*” OR “predictive value*” OR specificity OR sensitivity OR error* OR analytic* OR pre-analytic* OR preanalytic* OR quality OR cost* OR harm* OR recommend* OR guideline*)

## Cochrane CENTRAL (Wiley)

((blood NEAR/2 sampl*) OR (blood NEAR/2 culture*) OR (blood NEAR/2 specimen*) OR (blood NEAR/2 collect*) OR (blood NEAR/2 draw*) OR (blood NEAR/2 test*)):ti,ab

**AND**

((Venipuncture OR venapuncture OR venepuncture OR venupuncture OR venesection OR (venous NEAR/2 catheter*) OR (venous NEAR/2 sampl*) OR (venous NEAR/2 puncture*) OR (intravenous NEAR/2 catheter*) OR (indwelling NEAR/2 catheter*) OR (vein* NEAR/2 catheter*) OR (catheter* NEAR/2 draw*) OR (catheter* NEAR/2 culture*) OR (catheter* NEAR/2 collect*) OR (peripheral NEAR/2 culture*) OR (peripheral NEAR/2 catheter*) OR phlebotomy team* OR phlebotomist* OR (phlebotom* NEAR/2 method*) OR (phlebotom* NEAR/2 practice*) OR (prep* NEAR/2 kit*) OR (collection NEAR/2 kit*) OR antisepsis OR antiseptic OR antiinfective* OR anti-infective* OR disinfect* OR decontamina* OR chloraprep OR (skin NEAR/2 prep*) OR (patient* NEAR/2 prep*))):ti,ab

**AND**

(contamina* OR “false positive*” OR “false negative*” OR “clinical utility” OR “clinical use*” OR “predictive value*” OR specificity OR sensitivity OR error* OR analytic* OR pre-analytic* OR preanalytic* OR quality OR cost* OR harm* OR recommend* OR guideline*):ti,ab

## Scopus (Elsevier)

TITLE-ABS-KEY ( ( blood W/2 sampl* ) OR ( blood W/2 culture* ) OR ( blood W/2 specimen* ) OR ( blood W/2 collect* ) OR ( blood W/2 draw* ) OR ( blood W/2 test* ) )

**AND**

TITLE-ABS-KEY ( venipuncture OR venapuncture OR venepuncture OR venupuncture OR venesection OR ( venous W/2 catheter* ) OR ( venous W/2 sampl* ) OR ( venous W/2 puncture* ) OR ( intravenous W/2 catheter* ) OR ( indwelling W/2 catheter* ) OR ( vein* W/2 catheter* ) OR ( catheter* W/2 draw* ) OR ( catheter* W/2 culture* ) OR ( catheter* W/2 collect* ) OR ( peripheral W/2 culture* ) OR ( peripheral W/2 catheter* ) OR "phlebotomy team*" OR phlebotomist* OR ( phlebotom* W/2 method* ) OR ( phlebotom* W/2 practice* ) OR ( prep* W/2 kit* ) OR ( collection W/2 kit* ) OR antisepsis OR antiseptic OR antiinfective* OR anti-infective* OR disinfect* OR decontamina* OR chloraprep OR ( skin W/2 prep* ) OR ( patient* W/2 prep* ) )

**AND**

TITLE-ABS-KEY ( contamina* OR "false positive*" OR "false negative*" OR "clinical utility" OR "clinical use*" OR "predictive value*" OR specificity OR sensitivity OR error* OR analytic* OR pre-analytic* OR preanalytic* OR quality OR cost* OR harm* OR recommend* OR guideline* ) AND NOT INDEX ( medline ) AND NOT INDEX ( embase )

## ClinicalTrials.gov

("blood specimen collection" OR "blood culture")

**AND**

(contamination OR decontaminat* OR disinfect* OR "prep kit*" OR "phlebotomy team" OR antiinfective* OR antiseptic* OR antisepsis)

## Record Identification

### Medline (Ovid)

|  | **Initial Search** | **Update #1** | **Update #2** | **Update #3** | **Final Total** |
| --- | --- | --- | --- | --- | --- |
| Citations Retrieved | 3,113 | 61 | 207 | 983 | 4,364 |
| Duplicates | - | - | - | 207 | 207 |
| Unique Citations | 3,113 | 61 | 207 | 776 | 4,157 |

### Embase (Ovid/Elsevier)

|  | **Initial Search** | **Update #1** | **Update #2** | **Update #3** | **Final Total** |
| --- | --- | --- | --- | --- | --- |
| Citations Retrieved | 4,087 | 198 | 480 | 2,704 | 7,469 |
| Duplicates | 2,032 | 32 | 144 | 375 | 2,583 |
| Unique Citations | 2,055 | 166 | 336 | 2,329 | 4,886 |

### CINAHL (EBSCO)

|  | **Initial Search** | **Update #1** | **Update #2** | **Update #3** | **Final Total** |
| --- | --- | --- | --- | --- | --- |
| Citations Retrieved | 125 | 18 | 34 | 297 | 474 |
| Duplicates | 44 | 7 | 15 | 130 | 196 |
| Unique Citations | 81 | 11 | 19 | 167 | 278 |

### Cochrane CENTRAL (Wiley)

|  | **Initial Search** | **Update #1** | **Update #2** | **Update #3** | **Final Total** |
| --- | --- | --- | --- | --- | --- |
| Citations Retrieved | 242 | 0 | 167 | 478 | 887 |
| Duplicates | 201 | - | 13 | 56 | 270 |
| Unique Citations | 41 | 0 | 154 | 422 | 617 |

### Scopus (Elsevier)

|  | **Initial Search** | **Update #1** | **Update #2** | **Update #3** | **Final Total** |
| --- | --- | --- | --- | --- | --- |
| Citations Retrieved | 8 | 26 | 28 | 106 | 168 |
| Duplicates | 4 | 6 | 11 | 18 | 39 |
| Unique Citations | 4 | 20 | 17 | 88 | 129 |

### ClinicalTrials.gov

|  | **Initial Search** | **Update #1** | **Update #2** | **Update #3** | **Final Total** |
| --- | --- | --- | --- | --- | --- |
| Citations Retrieved | 1,054 | N/A | 0 | 21 | 1,075 |
| Duplicates |  | - | - | 0 | 0 |
| Unique Citations | 1,054 | N/A | 0 | 21 | 1,075 |

### NTIS + EconLit

|  | **Initial Search** | **Update #1** | **Update #2** | **Update #3** | **Final Total** |
| --- | --- | --- | --- | --- | --- |
| Citations Retrieved | 8 | 0 | 0 | 0 | 8 |
| Duplicates | 2 | 0 | 0 | 0 | 2 |
| Unique Citations | 6 | 0 | 0 | 0 | 6 |

### Hand Searching

|  | **Initial Search** | **Update #1** | **Update #2** | **Update #3** | **Final Total** |
| --- | --- | --- | --- | --- | --- |
| Citations Retrieved | 0 | 0 | 0 | 171 | 171 |

### Total Records Identified

|  | **Initial Search** | **Update #1** | **Update #2** | **Update #3** | **Final Total** |
| --- | --- | --- | --- | --- | --- |
| Citations Retrieved | 8,637 | 303 | 916 | 4,760 | 14,616 |
| Duplicates | 2,283 | 45 | 183 | 786 | 3,297 |
| Unique Citations | 6,354 | 258 | 733 | 3,974 | 11,319 |

## Record Screening

### Title Stage

|  | **Initial/Update #1** | **Update #2** | **Update #3** | **Total** |
| --- | --- | --- | --- | --- |
| Titles Reviewed | 6,612 | 733 | 3,974 | 11,319 |
| Excluded | 5,756 | 710 | 3,807 | 10,273 |
| Included | 856 | 23 | 167 | 1,046 |

### Abstract Stage

|  | **Initial/Update #1** | **Update #2** | **Update #3** | **Total** |
| --- | --- | --- | --- | --- |
| Abstracts Reviewed | 856 | 23 | 167 | 1,046 |
| Excluded | 606 | 7 | 102 | 724 |
| Included | 250 | 16 | 65 | 332 |

### Full-Text Stage

|  | **SRDR #1** | **SRDR #2** | **Total** |
| --- | --- | --- | --- |
| Full-Text Sought | 266 | 65 | 331 |
| No Full-Text | 139 | 15 | 154 |
| *Full-Text Reviewed* | 127 | 50 | 177 |
| Published >2010 | 42 | 0 | 42 |
| Conference Abstract | 35 | 24 | 59 |
| Insufficient Data | 0 | 4 | 4 |
| Non-English | 0 | 2 | 2 |
| Duplicate | 1 | 2 | 2 |
| Clinical Trial | 0 | 4 | 4 |
| Off Topic | 8 | 0 | 8 |
| No Intervention | 6 | 0 | 6 |
| Excluded Records | 92 | 36 | 128 |
| Extracted Records | 35 | 14 | 49 |

## Methodology

### Guideline Updates

The 2023 update of the blood culture contamination guidelines, the ASM/RU Guidelines Committee reviewed and analyzed data published from January 1995 through September 2021. This update was conducted in several stages, with initial searches conducted in October 2017 and February 2018, followed by updates in December 2018 and September 2021.

### Literature Sources & Strategies

The initial search was created and conducted by a medical librarian (CD) in October 2017 in 8 databases: MEDLINE (*Ovid*), Embase (*Ovid*), Cochrane Central Register of Clinical Trials (*Wiley*), CINAHL (*EBSCO*), Scopus (*Elsevier*), EconLit (*EBSCO*), ClinicalTrials.gov, and the National Technical Information Service (*ntis.gov*). The updated searches were conducted by a medical librarian in February 2018 and December 2018 in a subset of the initial 8 databases.

The final update in September 2022 was conducted by a second medical librarian (LB) and included the following 6 databases: MEDLINE (*Ovid*), Embase (*Elsevier*), Cochrane Central Register of Clinical Trials (Wiley), CINHAL (*EBSCO*), Scopus (*Elsevier*), and ClinicalTrials.gov. After the September 2022 update, retrieved systematic reviews were then hand searched for additional records.

The search strategies consisted of keyword searches, and when applicable controlled vocabulary related to blood cultures, phlebotomy, infection control, and contamination in each database, using both Boolean operators and proximity searching to focus the search results (Appendix A).

### Data Collection & Analysis

Records were deduplicated, organized, and stored using the bibliographic management system EndNote and systematic review management system Covidence. Duplicates were identified using automated duplicate identification in EndNote and Covidence. Screening occurred at both the title and abstract stage, followed by the full-text stage concurrent with data extraction, using inclusion/exclusion criteria developed *a priori*.

Across all the searches conducted, a total of 14,616 total records were identified, and following the automatic removal of duplicates there were a total of 11,319 unique records. From the 11,319 unique records identified, 331 were included in the full-text screening, of which 282 were excluded based on pre-defined inclusion and exclusion criteria. Data was extracted from a total of 49 articles included in the final analysis (Figure).

Figure depicts the Preferred Reporting Items for Systematic Reviews and Meta-Analyses (PRISMA) flow diagram for the systematic review process.


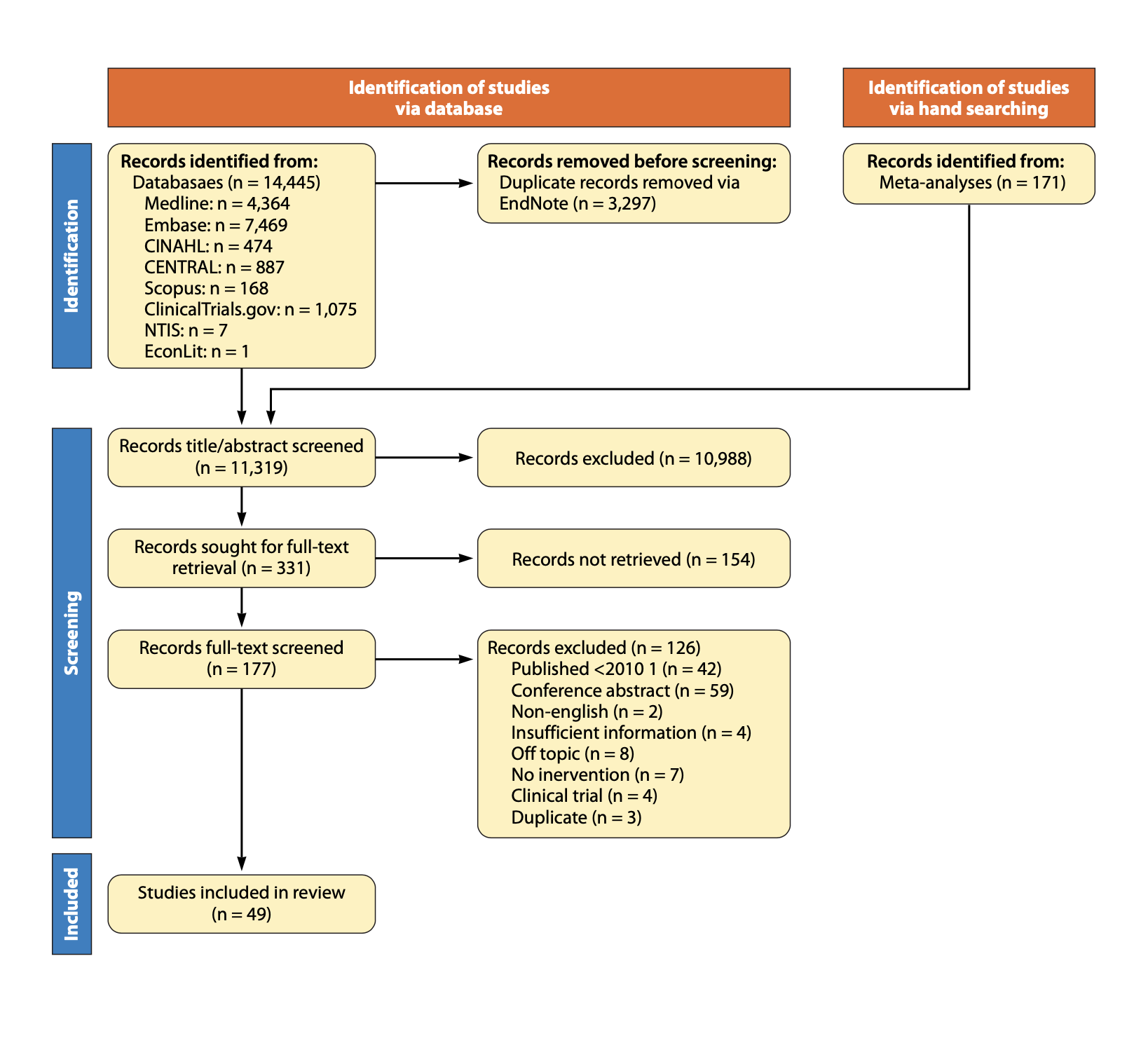

Supplement: ASM-BCC Guideline Documentation — Details of the search history, search strategy, records identification, screening, and methodology. [file cmr.00087-24-s0003.docx]
