# Supplementary material for: American Society for Microbiology evidence-based laboratory medicine practice guidelines to reduce blood culture contamination rates: a systematic review and meta-analysis
Source: Clin Microbiol Rev. 2024 Nov 4;37(4):e00087-24. doi: 10.1128/cmr.00087-24 (PMC11629619; doi:10.1128/cmr.00087-24)

ASM BCC Meta-Analysis
Main Analyses
Hypothesis Tests
General Sources of Heterogeneity
Analysis for GRADE
Discrete Interventions
ASM BCC Meta-Analysis
Main Analyses
Hypothesis Tests
General Sources of Heterogeneity
Analysis for GRADE
Discrete Interventions

BCC Meta-Analyses

J. Scott Parrott, Christen Diel, Ryan Tom

2024-07-19

ASM BCC Meta-Analysis

Preparation

Here we will set up by pulling in the most recent datafiles and then formatting the objects that we'll use for the meta-analyses.

Load All Packages

```
library(tidyverse)
```

```
## Warning: package 'ggplot2' was built under R version 4.3.2
```

```
## Warning: package 'tidyr' was built under R version 4.3.3
```

```
## Warning: package 'readr' was built under R version 4.3.3
```

```
## Warning: package 'purrr' was built under R version 4.3.3
```

```
## Warning: package 'dplyr' was built under R version 4.3.3
```

```
## Warning: package 'stringr' was built under R version 4.3.3
```

```
## ── Attaching core tidyverse packages ──────────────────────── tidyverse 2.0.0 ──
## ✔ dplyr     1.1.4     ✔ readr     2.1.5
## ✔ forcats   1.0.0     ✔ stringr   1.5.1
## ✔ ggplot2   3.4.4     ✔ tibble    3.2.1
## ✔ lubridate 1.9.2     ✔ tidyr     1.3.1
## ✔ purrr     1.0.2
## ── Conflicts ────────────────────────────────────────── tidyverse_conflicts() ──
## ✖ dplyr::filter() masks stats::filter()
## ✖ dplyr::lag()    masks stats::lag()
## ℹ Use the conflicted package (<http://conflicted.r-lib.org/>) to force all conflicts to become err
ors
```

```
library(dplyr)
library(meta)
```

```
## Loading 'meta' package (version 6.5-0).
## Type 'help(meta)' for a brief overview.
## Readers of 'Meta-Analysis with R (Use R!)' should install
## older version of 'meta' package: https://tinyurl.com/dt4y5drs
```

```
library(metafor)
```

```
## Loading required package: Matrix
```

```
## Warning: package 'Matrix' was built under R version 4.3.2
```

```
##
## Attaching package: 'Matrix'
##
## The following objects are masked from 'package:tidyr':
##
##     expand, pack, unpack
##
## Loading required package: metadat
## Loading required package: numDeriv
##
## Loading the 'metafor' package (version 4.2-0). For an
## introduction to the package please type: help(metafor)
```

```
library(dmetar)
```

```
## Extensive documentation for the dmetar package can be found at:
##  www.bookdown.org/MathiasHarrer/Doing_Meta_Analysis_in_R/
```

```
library(ggplot2)
library(gridExtra)
```

```
##
## Attaching package: 'gridExtra'
##
## The following object is masked from 'package:dplyr':
##
##     combine
```

```
library(knitr)
```

```
## Warning: package 'knitr' was built under R version 4.3.3
```

Import and format the data

One dataset includes results by hospital unit, so multiple lines per study (BCC_Outcomes). The other dataset includes one row per study (units were combined for an overall facility level result: BCC_Outcomes1a).

```
library(readxl)
BCC_One_Arm_per_study_for_publication_and_rob_subanalyses_2_6_24 <- read_excel("C:/Users/Scott/OneDrive - Rutgers University/ASM/ASM BCC Project/3.0 Data/3.4 BCC Meta-Analyses/3.2.2 Data/BCC One Arm per study for publication and rob subanalyses 2-6-24.xlsx")

BCC_consolidated_arms_for_analysis_2_6_24 <- read_excel("C:/Users/Scott/OneDrive - Rutgers University/ASM/ASM BCC Project/3.0 Data/3.4 BCC Meta-Analyses/3.2.2 Data/BCC consolidated arms for analysis 2-6-24.xlsx")

# Give the file a new shorter name for convenience [need to modify to fit ]
BCC_Outcomes1a <- BCC_One_Arm_per_study_for_publication_and_rob_subanalyses_2_6_24

BCC_Outcomes <- BCC_consolidated_arms_for_analysis_2_6_24
```

Break up dataset into separate units in order to get more detailed results by hospital unit.This will give us all the data objects we need to work with.

```
Combined_units <- dplyr::filter(BCC_Outcomes,`Unit2` %in% c("Combined Units"))
Ed <- dplyr::filter(BCC_Outcomes,`Unit2` %in% c("ED"))
Gen_units <- dplyr::filter(BCC_Outcomes,`Unit2` %in% c("General Units"))
ICUHeme_onc <- dplyr::filter(BCC_Outcomes,`Unit2` %in% c("ICU/Heme/Onc"))
Pedes <- dplyr::filter(BCC_Outcomes,`Unit2` %in% c("Pedes Units"))
```

Main Analyses

First, we will run an overall meta-analysis on the dataset with one row per study.

```
BCC_All1.bin <- metabin(Ee,
                       Ne,
                       Ec,
                       Nc,
                       data = BCC_Outcomes1a, #this is my data set
                       studlab = paste(Author, year),
                       comb.fixed = FALSE,
                       comb.random = TRUE, #here I'm doing a random effects model
                       method.tau = "PM", #I'm going to use a PM first
                       hakn = FALSE,
                       prediction = TRUE,
                       incr = 0.1,
                       sm = "RR")

BCC_All1.bin
```

```
## Number of studies: k = 53
## Number of observations: o = 958387
## Number of events: e = 18545.67
##
##                          RR           95%-CI     z  p-value
## Random effects model 0.4784 [0.3997; 0.5727] -8.04 < 0.0001
## Prediction interval         [0.1442; 1.5872]
##
## Quantifying heterogeneity:
##  tau^2 = 0.3484 [0.2094; 0.6188]; tau = 0.5902 [0.4576; 0.7867]
##  I^2 = 89.8% [87.4%; 91.7%]; H = 3.13 [2.82; 3.47]
##
## Test of heterogeneity:
##       Q d.f.  p-value
##  508.34   52 < 0.0001
##
## Details on meta-analytical method:
## - Inverse variance method
## - Paule-Mandel estimator for tau^2
## - Q-Profile method for confidence interval of tau^2 and tau
## - Prediction interval based on t-distribution (df = 51)
## - Continuity correction of 0.1 in studies with zero cell frequencies
```

Now we'll generate a forest plot (maybe)

```
png(file = "BCC_ALL_updated 2-9-24.png", width = 2800, height = 4800, res = 300)

forest(BCC_All1.bin, sortvar = TE, xlab = "Favors Innovation -- Favors Usual")

dev.off()
```

```
## png
##   2
```

```
knitr::include_graphics("BCC_ALL_updated 2-9-24.png")
```

Study	Experimental Events	Experimental Total	Control Events	Control Total	RR	95%-CI	Weight
Nuntnarumit 2013	0	172	5	172	0.02	[0.00; 10.21]	0.1%
Sweet 2012	1	470	17	692	0.09	[0.01; 0.65]	0.6%
Frota 2021	2	200	20	200	0.10	[0.02; 0.42]	0.9%
Maeda 2021	2	711	17	749	0.12	[0.03; 0.53]	0.9%
Rupp 2017	2	904	16	904	0.12	[0.03; 0.54]	0.9%
Ge 2011	5	3177	77	6144	0.13	[0.05; 0.31]	1.5%
He 2020	19	3228	190	4208	0.13	[0.08; 0.21]	2.1%
Bell 2018	38	6293	1246	35392	0.17	[0.12; 0.24]	2.2%
Kai 2020	5	450	29	460	0.18	[0.07; 0.45]	1.5%
HalsteadD 2020	6	303	154	1497	0.19	[0.09; 0.43]	1.6%
O'Connor 2016	3	314	17	364	0.20	[0.06; 0.69]	1.1%
HalsteadA 2020	44	4604	959	25315	0.25	[0.19; 0.34]	2.3%
Levin 2013	9	172	27	141	0.27	[0.13; 0.56]	1.7%
Al-Hamad 2019	5	222	17	222	0.29	[0.11; 0.78]	1.4%
Tarrand 2012	3	326	10	331	0.30	[0.08; 1.10]	1.1%
Moeller 2017	74	4200	226	4200	0.33	[0.25; 0.42]	2.3%
Lalezari 2020	6	356	20	400	0.34	[0.14; 0.83]	1.5%
Self2 2014	17	1860	63	2509	0.36	[0.21; 0.62]	2.0%
Kamboj 2015	146	12048	342	10778	0.38	[0.31; 0.46]	2.4%
HalsteadB 2020	117	13557	333	14764	0.38	[0.31; 0.47]	2.3%
Self 2013	111	6590	321	7389	0.39	[0.31; 0.48]	2.3%
Zimmerman 2019	7	325	17	312	0.40	[0.17; 0.94]	1.5%
Sanchez 2018	47	841	22	158	0.40	[0.25; 0.65]	2.1%
Hall 2013	35	2153	212	5402	0.41	[0.29; 0.59]	2.2%
Thomas 2011	29	705	90	981	0.45	[0.30; 0.67]	2.1%
Allen 2021	10	1110	39	1944	0.45	[0.23; 0.90]	1.8%
Krajčinović 2015	8	105	22	134	0.46	[0.22; 1.00]	1.7%
Shaheen 2020	380	9756	709	8868	0.49	[0.43; 0.55]	2.4%
El Feghaly 2018	578	37500	1603	56250	0.54	[0.49; 0.59]	2.4%
Harding 2013	64	6336	101	5544	0.55	[0.41; 0.76]	2.3%
Self1 2014	142	5238	165	3417	0.56	[0.45; 0.70]	2.3%
Patton 2010	58	2672	41	1061	0.56	[0.38; 0.83]	2.2%
Ramirez 2015	40	308	80	348	0.56	[0.40; 0.80]	2.2%
Kim 2011	34	5265	60	5255	0.57	[0.37; 0.86]	2.1%
Ryan 2017	21	1152	130	4197	0.59	[0.37; 0.93]	2.1%
Lin 2012	31	1548	328	9645	0.59	[0.41; 0.85]	2.2%
Bae 2019	222	83534	354	78673	0.59	[0.50; 0.70]	2.4%
Al-Hamad 2016	231	4138	389	4521	0.65	[0.55; 0.76]	2.4%
Marini 2013	840	60000	1260	60000	0.67	[0.61; 0.73]	2.4%
Marlowe 2010	72	4274	122	4942	0.68	[0.51; 0.91]	2.3%
Syed 2020	239	14046	326	13350	0.70	[0.59; 0.82]	2.4%
Santos 2018	2	234	4	329	0.70	[0.13; 3.81]	0.8%
Tangsathapompong 2014	14	615	21	654	0.71	[0.36; 1.38]	1.8%
HalsteadC 2020	172	12507	215	11100	0.71	[0.58; 0.87]	2.3%
Yodoshi 2019	38	2407	65	2958	0.72	[0.48; 1.07]	2.2%
Yu 2020	29	549	40	549	0.72	[0.46; 1.15]	2.1%
Park 2015	922	83861	1212	88476	0.80	[0.74; 0.87]	2.4%
Roth 2010	278	12459	1006	38805	0.86	[0.75; 0.98]	2.4%
Murillo 2011	124	2531	114	2280	0.98	[0.76; 1.26]	2.3%
Story-Roller 2016	123	3130	115	2965	1.01	[0.79; 1.30]	2.3%
McLaughlin 2013	11	284	16	693	1.68	[0.79; 3.57]	1.7%
Martínez 2017	10	539	5	563	2.09	[0.72; 6.07]	1.3%
Tarai 2012	83	2553	47	4350	3.01	[2.11; 4.29]	2.2%

Risk Ratio

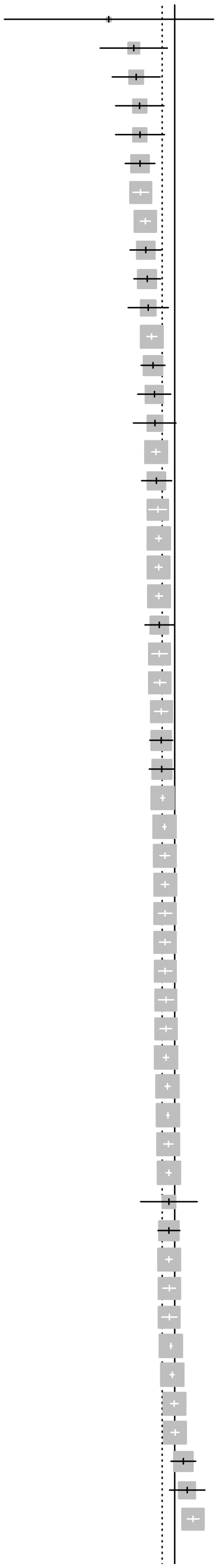

**Random effects model**	**422832**	**535555**		**0.48 [0.40; 0.57]**	**100.0%**
**Prediction interval**				**[0.14; 1.59]**	

Heterogeneity: $I^2 = 90\%$, $\tau^2 = 0.3484$, $p < 0.01$

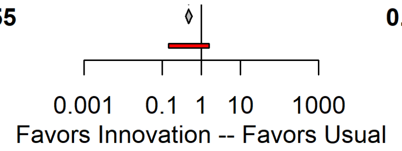

Subgroup Analyses by Unit

We will use the other dataset for this.

```
BCC_All.bin <- metabin(Ee,
                        Ne,
                        Ec,
                        Nc,
                        data = BCC_Outcomes, #this is my data set
                        studlab = paste(Author, year),
                        comb.fixed = FALSE,
                        comb.random = TRUE, #here I'm doing a random effects model
                        method.tau = "PM", #I'm going to use a PM first
                        hakn = FALSE,
                        prediction = TRUE,
                        incr = 0.1,
                        sm = "RR")

BCC_All.bin
```

```
## Number of studies: k = 65
## Number of observations: o = 1168271
## Number of events: e = 20415.67
##
##                           RR           95%-CI     z  p-value
## Random effects model 0.5179 [0.4413; 0.6078] -8.06 < 0.0001
## Prediction interval         [0.1608; 1.6679]
##
## Quantifying heterogeneity:
##  tau^2 = 0.3358 [0.2052; 0.5782]; tau = 0.5795 [0.4530; 0.7604]
##  I^2 = 88.0% [85.4%; 90.1%]; H = 2.89 [2.62; 3.18]
##
## Test of heterogeneity:
##       Q d.f.  p-value
##  533.40   64 < 0.0001
##
## Details on meta-analytical method:
## - Inverse variance method
## - Paule-Mandel estimator for tau^2
## - Q-Profile method for confidence interval of tau^2 and tau
## - Prediction interval based on t-distribution (df = 63)
## - Continuity correction of 0.1 in studies with zero cell frequencies
```

```
## Subgroup analysis by Unit ####

### Results by Unit ####
unit.subgroup<-update.meta(BCC_All.bin,
                                          byvar=Unit2,
                                          comb.random = TRUE,
                                          comb.fixed = FALSE,
                                          bysort=TRUE)
unit.subgroup
```

```
## Number of studies: k = 65
## Number of observations: o = 1168271
## Number of events: e = 20415.67
##
##                          RR           95%-CI     z  p-value
## Random effects model 0.5179 [0.4413; 0.6078] -8.06 < 0.0001
## Prediction interval         [0.1608; 1.6679]
##
## Quantifying heterogeneity:
##  tau^2 = 0.3358 [0.2052; 0.5782]; tau = 0.5795 [0.4530; 0.7604]
##  I^2 = 88.0% [85.4%; 90.1%]; H = 2.89 [2.62; 3.18]
##
## Test of heterogeneity:
##       Q d.f.  p-value
##  533.40   64 < 0.0001
##
## Results for subgroups (random effects model):
##                          k     RR           95%-CI  tau^2    tau      Q   I^2
## Unit2 = ED              18 0.4873 [0.3577; 0.6640] 0.3557 0.5964 128.50 86.8%
## Unit2 = General Units    9 0.5681 [0.3858; 0.8366] 0.2742 0.5236  22.84 65.0%
## Unit2 = ICU/Heme/Onc    10 0.3390 [0.2347; 0.4896] 0.2440 0.4940  38.08 76.4%
## Unit2 = Pedes Units     12 0.5558 [0.4086; 0.7561] 0.1804 0.4247  32.95 66.6%
## Unit2 = Combined Units  16 0.6591 [0.4734; 0.9175] 0.3805 0.6168 190.77 92.1%
##
## Test for subgroup differences (random effects model):
##                    Q d.f. p-value
## Between groups 7.71    4  0.1028
##
## Details on meta-analytical method:
## - Inverse variance method
## - Paule-Mandel estimator for tau^2
## - Q-Profile method for confidence interval of tau^2 and tau
## - Prediction interval based on t-distribution (df = 63)
## - Continuity correction of 0.1 in studies with zero cell frequencies
```

Now, in order to get more detail (e.g., the CI for the heterogeneity), we will need to carry out the meta-analyses on each unit separately.

Combined Units

```
BCC_combined.bin <- metabin(Ee,
                       Ne,
                       Ec,
                       Nc,
                       data = Combined_units, #this is my data set
                       studlab = paste(Author, year),
                       comb.fixed = FALSE,
                       comb.random = TRUE, #here I'm doing a random effects model
                       method.tau = "PM", #I'm going to use a PM first
                       hakn = FALSE,
                       prediction = TRUE,
                       incr = 0.1,
                       sm = "RR")

BCC_combined.bin
```

```
## Number of studies: k = 16
## Number of observations: o = 512745
## Number of events: e = 7422
##
##                          RR           95%-CI     z p-value
## Random effects model 0.6591 [0.4734; 0.9175] -2.47  0.0135
## Prediction interval         [0.1672; 2.5978]
##
## Quantifying heterogeneity:
##  tau^2 = 0.3805 [0.1747; 1.0469]; tau = 0.6168 [0.4180; 1.0232]
##  I^2 = 92.1% [88.8%; 94.5%]; H = 3.57 [2.99; 4.25]
##
## Test of heterogeneity:
##       Q d.f.  p-value
##  190.77   15 < 0.0001
##
## Details on meta-analytical method:
## - Inverse variance method
## - Paule-Mandel estimator for tau^2
## - Q-Profile method for confidence interval of tau^2 and tau
## - Prediction interval based on t-distribution (df = 14)
```

Ed Units

```
BCC_ED.bin <- metabin(Ee,
                            Ne,
                            Ec,
                            Nc,
                            data = Ed, #this is my data set
                            studlab = paste(Author, year),
                            comb.fixed = FALSE,
                            comb.random = TRUE, #here I'm doing a random effects model
                            method.tau = "PM", #I'm going to use a PM first
                            hakn = FALSE,
                            prediction = TRUE,
                            incr = 0.1,
                            sm = "RR")

BCC_ED.bin
```

```
## Number of studies: k = 18
## Number of observations: o = 150857
## Number of events: e = 4993.918
##
##                          RR           95%-CI     z  p-value
## Random effects model 0.4873 [0.3577; 0.6640] -4.55 < 0.0001
## Prediction interval         [0.1318; 1.8022]
##
## Quantifying heterogeneity:
##  tau^2 = 0.3557 [0.1390; 1.1130]; tau = 0.5964 [0.3728; 1.0550]
##  I^2 = 86.8% [80.5%; 91.0%]; H = 2.75 [2.27; 3.33]
##
## Test of heterogeneity:
##       Q d.f.  p-value
##  128.50   17 < 0.0001
##
## Details on meta-analytical method:
## - Inverse variance method
## - Paule-Mandel estimator for tau^2
## - Q-Profile method for confidence interval of tau^2 and tau
## - Prediction interval based on t-distribution (df = 16)
```

General Units

```
BCC_General.bin <- metabin(Ee,
                      Ne,
                      Ec,
                      Nc,
                      data = Gen_units, #this is my data set
                      studlab = paste(Author, year),
                      comb.fixed = FALSE,
                      comb.random = TRUE, #here I'm doing a random effects model
                      method.tau = "PM", #I'm going to use a PM first
                      hakn = FALSE,
                      prediction = TRUE,
                      incr = 0.1,
                      sm = "RR")

BCC_General.bin
```

```
## Number of studies: k = 9
## Number of observations: o = 157363
## Number of events: e = 1620
##
##                          RR           95%-CI     z p-value
## Random effects model 0.5681 [0.3858; 0.8366] -2.86  0.0042
## Prediction interval         [0.1513; 2.1338]
##
## Quantifying heterogeneity:
##  tau^2 = 0.2742 [0.0309; 1.6110]; tau = 0.5236 [0.1757; 1.2693]
##  I^2 = 65.0% [28.6%; 82.8%]; H = 1.69 [1.18; 2.41]
##
## Test of heterogeneity:
##      Q d.f. p-value
##  22.84    8  0.0036
##
## Details on meta-analytical method:
## - Inverse variance method
## - Paule-Mandel estimator for tau^2
## - Q-Profile method for confidence interval of tau^2 and tau
## - Prediction interval based on t-distribution (df = 7)
```

ICU/Heme/onc Units

```
BCC_ICUHeme_onc <- metabin(Ee,
                           Ne,
                           Ec,
                           Nc,
                           data = ICUHeme_onc, #this is my data set
                           studlab = paste(Author, year),
                           comb.fixed = FALSE,
                           comb.random = TRUE, #here I'm doing a random effects model
                           method.tau = "PM", #I'm going to use a PM first
                           hakn = FALSE,
                           prediction = TRUE,
                           incr = 0.1,
                           sm = "RR")

BCC_ICUHeme_onc
```

```
## Number of studies: k = 10
## Number of observations: o = 104240
## Number of events: e = 1379.752
##
##                           RR           95%-CI     z  p-value
## Random effects model 0.3390 [0.2347; 0.4896] -5.77 < 0.0001
## Prediction interval         [0.1002; 1.1465]
##
## Quantifying heterogeneity:
##  tau^2 = 0.2440 [0.0609; 1.3298]; tau = 0.4940 [0.2468; 1.1532]
##  I^2 = 76.4% [56.3%; 87.2%]; H = 2.06 [1.51; 2.80]
##
## Test of heterogeneity:
##      Q d.f.  p-value
##  38.08    9 < 0.0001
##
## Details on meta-analytical method:
## - Inverse variance method
## - Paule-Mandel estimator for tau^2
## - Q-Profile method for confidence interval of tau^2 and tau
## - Prediction interval based on t-distribution (df = 8)
```

Pedes Units

```
BCC_Pedes <- metabin(Ee,
                           Ne,
                           Ec,
                           Nc,
                           data = Pedes, #this is my data set
                           studlab = paste(Author, year),
                           comb.fixed = FALSE,
                           comb.random = TRUE, #here I'm doing a random effects model
                           method.tau = "PM", #I'm going to use a PM first
                           hakn = FALSE,
                           prediction = TRUE,
                           incr = 0.1,
                           sm = "RR")

BCC_Pedes
```

```
## Number of studies: k = 12
## Number of observations: o = 243066
## Number of events: e = 5000
##
##                           RR           95%-CI     z p-value
## Random effects model 0.5558 [0.4086; 0.7561] -3.74  0.0002
## Prediction interval         [0.2026; 1.5243]
##
## Quantifying heterogeneity:
##  tau^2 = 0.1804 [0.0113; 1.2709]; tau = 0.4247 [0.1064; 1.1274]
##  I^2 = 66.6% [38.7%; 81.8%]; H = 1.73 [1.28; 2.35]
##
## Test of heterogeneity:
##      Q d.f. p-value
##  32.95   11  0.0005
##
## Details on meta-analytical method:
## - Inverse variance method
## - Paule-Mandel estimator for tau^2
## - Q-Profile method for confidence interval of tau^2 and tau
## - Prediction interval based on t-distribution (df = 10)
## - Continuity correction of 0.1 in studies with zero cell frequencies
```

Outlier and Influence Analysis by Unit

While the above analyses provide a pooled estimate of the effects of the intervention by unit, some interventions “under perform” while others “over perform”. By detecting and dropping these studies, we may be able to get a sense of what a more typical performance outcome may be.

So, for each of the above units, we provide an outlier and influence estimate to determine how sensitive the initial estimates are to these under- and over-performers.

Combined Units

```
find.outliers(BCC_combined.bin)
```

```
## Identified outliers (random-effects model)
## ------------------------------------------
## "HalsteadA 2020", "HalsteadB 2020", "HalsteadD 2020", "Tarai 2012"
##
## Results with outliers removed
## -----------------------------
## Number of studies: k = 12
## Number of observations: o = 512745
## Number of events: e = 7422
##
##                           RR           95%-CI     z  p-value
## Random effects model 0.7242 [0.6250; 0.8391] -4.29 < 0.0001
## Prediction interval         [0.4586; 1.1435]
##
## Quantifying heterogeneity:
##  tau^2 = 0.0364 [0.0055; 0.3179]; tau = 0.1907 [0.0742; 0.5638]
##  I^2 = 67.0% [39.5%; 82.0%]; H = 1.74 [1.29; 2.36]
##
## Test of heterogeneity:
##      Q d.f. p-value
##  33.35   11  0.0005
##
## Details on meta-analytical method:
## - Inverse variance method
## - Paule-Mandel estimator for tau^2
## - Q-Profile method for confidence interval of tau^2 and tau
## - Prediction interval based on t-distribution (df = 10)
```

```
combined.inf <- InfluenceAnalysis(BCC_combined.bin, random = TRUE)
```

```
## [===========================================================================] DONE
```

```
plot(combined.inf, "baujat")
```

```
## Warning: ggrepel: 6 unlabeled data points (too many overlaps). Consider
## increasing max.overlaps
```

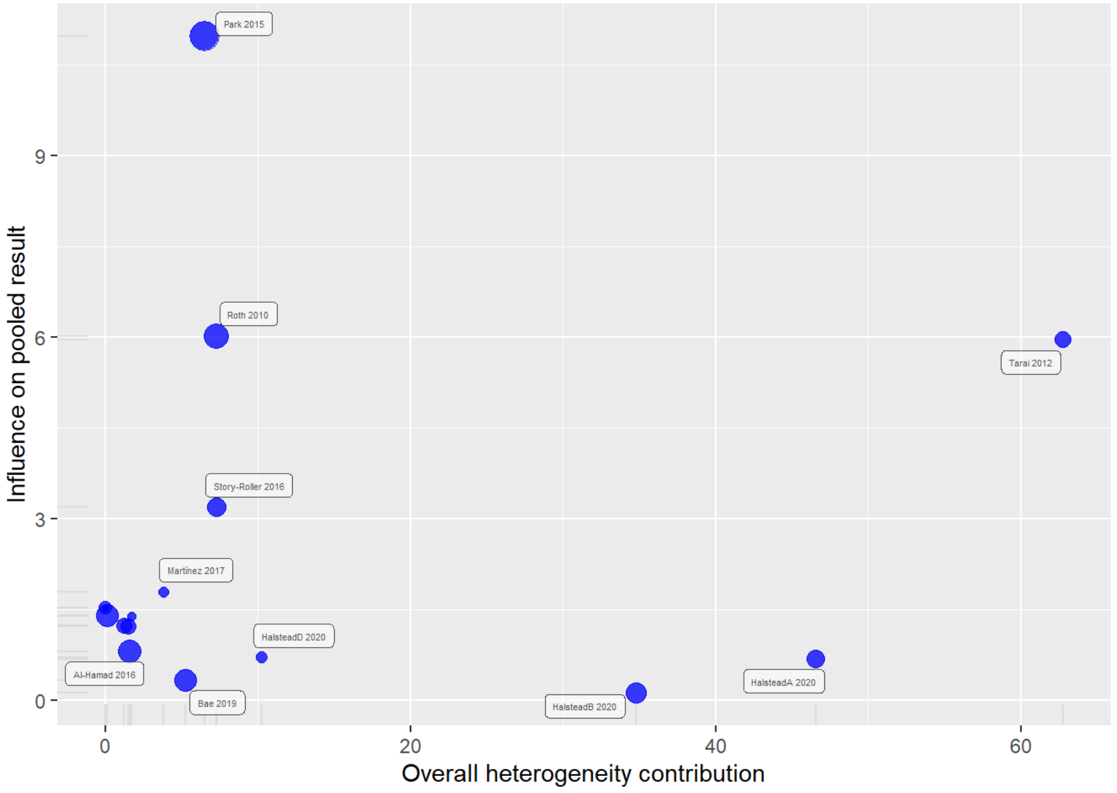

ED Units

```
find.outliers(BCC_ED.bin)
```

```
## Identified outliers (random-effects model)
## ------------------------------------------
## "Al-Hamad 2016", "Bell 2018", "Murillo 2011"
##
## Results with outliers removed
## -----------------------------
## Number of studies: k = 15
## Number of observations: o = 150857
## Number of events: e = 4993.918
##
##                           RR           95%-CI     z  p-value
## Random effects model 0.4721 [0.3795; 0.5873] -6.74 < 0.0001
## Prediction interval         [0.2171; 1.0264]
##
## Quantifying heterogeneity:
##  tau^2 = 0.1168 [0.0204; 0.7509]; tau = 0.3418 [0.1428; 0.8665]
##  I^2 = 68.4% [46.0%; 81.5%]; H = 1.78 [1.36; 2.32]
##
## Test of heterogeneity:
##      Q d.f.  p-value
##  44.25   14 < 0.0001
##
## Details on meta-analytical method:
## - Inverse variance method
## - Paule-Mandel estimator for tau^2
## - Q-Profile method for confidence interval of tau^2 and tau
## - Prediction interval based on t-distribution (df = 13)
```

```
ED.inf <- InfluenceAnalysis(BCC_ED.bin, random = TRUE)
```

```
## [===========================================================================] DONE
```

```
plot(ED.inf, "baujat")
```

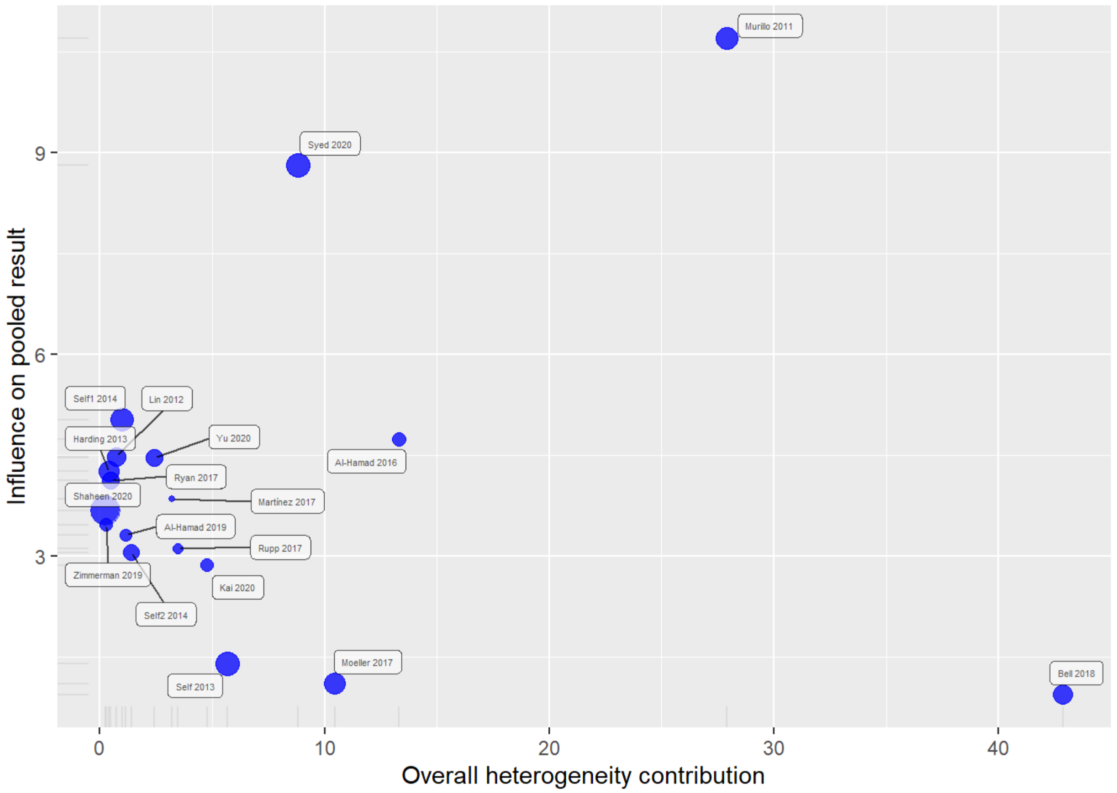

General Units

```
find.outliers(BCC_General.bin)
```

```
## Identified outliers (random-effects model)
## ------------------------------------------
## "Ge 2011"
##
## Results with outliers removed
## -----------------------------
## Number of studies: k = 8
## Number of observations: o = 157363
## Number of events: e = 1620
##
##                          RR           95%-CI     z  p-value
## Random effects model 0.6548 [0.5528; 0.7757] -4.90 < 0.0001
## Prediction interval         [0.4184; 1.0248]
##
## Quantifying heterogeneity:
##  tau^2 = 0.0260 [0.0000; 0.6482]; tau = 0.1613 [0.0000; 0.8051]
##  I^2 = 29.3% [0.0%; 68.4%]; H = 1.19 [1.00; 1.78]
##
## Test of heterogeneity:
##     Q d.f. p-value
##  9.90    7  0.1942
##
## Details on meta-analytical method:
## - Inverse variance method
## - Paule-Mandel estimator for tau^2
## - Q-Profile method for confidence interval of tau^2 and tau
## - Prediction interval based on t-distribution (df = 6)
```

```
Gen.inf <- InfluenceAnalysis(BCC_General.bin, random = TRUE)
```

```
## [===========================================================================] DONE
```

```
plot(Gen.inf, "baujat")
```

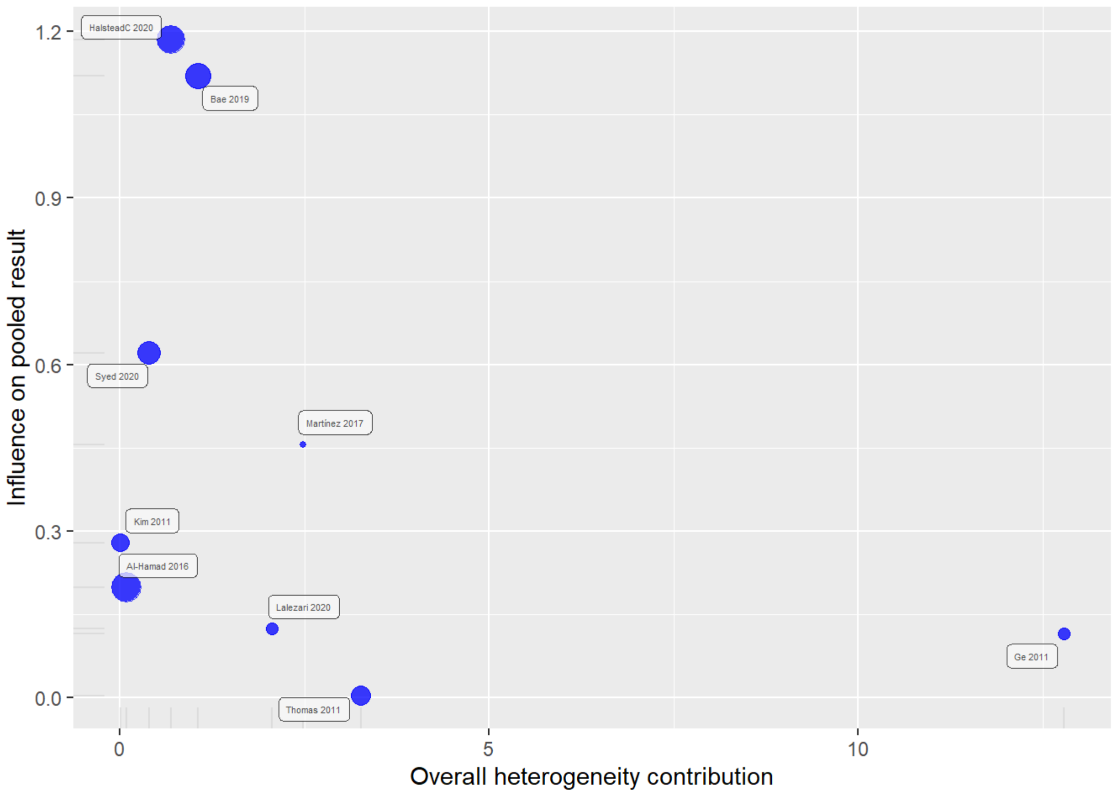

ICU/Heme/onc Units

```
find.outliers(BCC_ICUHeme_onc)
```

```
## Warning in find.outliers(BCC_ICUHeme_onc): Studies with NAs not considered in
## outlier analysis.
```

```
## Identified outliers (random-effects model)
## ------------------------------------------
## "He 2020"
##
## Results with outliers removed
## -----------------------------
## Number of studies: k = 9
## Number of observations: o = 104240
## Number of events: e = 1379.752
##
##                          RR           95%-CI     z  p-value
## Random effects model 0.4127 [0.3160; 0.5388] -6.50 < 0.0001
## Prediction interval         [0.1924; 0.8851]
##
## Quantifying heterogeneity:
##  tau^2 = 0.0856 [0.0000; 1.2831]; tau = 0.2926 [0.0000; 1.1328]
##  I^2 = 44.9% [0.0%; 74.5%]; H = 1.35 [1.00; 1.98]
##
## Test of heterogeneity:
##      Q d.f. p-value
##  14.53    8  0.0690
##
## Details on meta-analytical method:
## - Inverse variance method
## - Paule-Mandel estimator for tau^2
## - Q-Profile method for confidence interval of tau^2 and tau
## - Prediction interval based on t-distribution (df = 7)
```

```
Heme_onc.inf <- InfluenceAnalysis(BCC_ICUHeme_onc, random = TRUE)
```

```
## [===========================================================================] DONE
```

```
plot(Heme_onc.inf, "baujat")
```

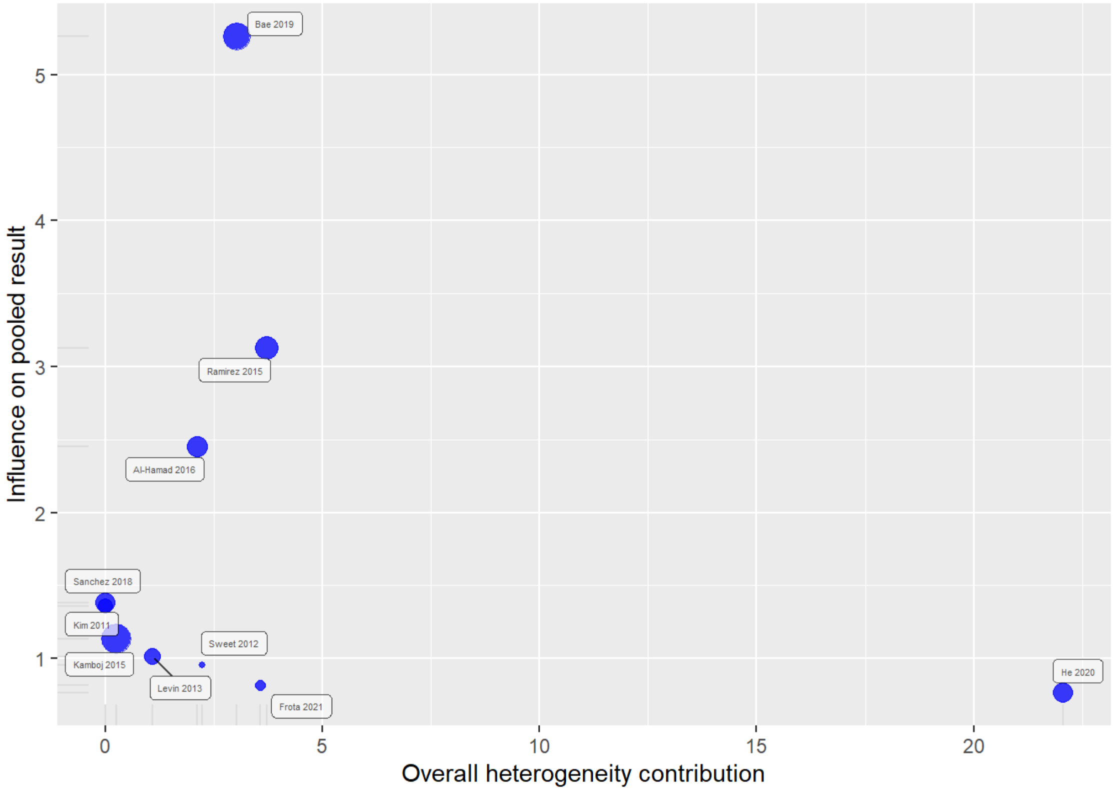

Pedes Units

```
find.outliers(BCC_Pedes)
```

```
## Identified outliers (random-effects model)
## ------------------------------------------
## "McLaughlin 2013"
##
## Results with outliers removed
## -----------------------------
## Number of studies: k = 11
## Number of observations: o = 243066
## Number of events: e = 5000
##
##                           RR           95%-CI      z  p-value
## Random effects model 0.5418 [0.4367; 0.6721] -5.57 < 0.0001
## Prediction interval         [0.2938; 0.9989]
##
## Quantifying heterogeneity:
##  tau^2 = 0.0611 [0.0019; 0.9034]; tau = 0.2471 [0.0436; 0.9505]
##  I^2 = 61.3% [25.4%; 80.0%]; H = 1.61 [1.16; 2.23]
##
## Test of heterogeneity:
##      Q d.f. p-value
##  25.85   10  0.0039
##
## Details on meta-analytical method:
## - Inverse variance method
## - Paule-Mandel estimator for tau^2
## - Q-Profile method for confidence interval of tau^2 and tau
## - Prediction interval based on t-distribution (df = 9)
## - Continuity correction of 0.1 in studies with zero cell frequencies
```

```
Pedes.inf <- InfluenceAnalysis(BCC_Pedes, random = TRUE)
```

```
## [==========================================================================] DONE
```

```
plot(Pedes.inf, "baujat")
```

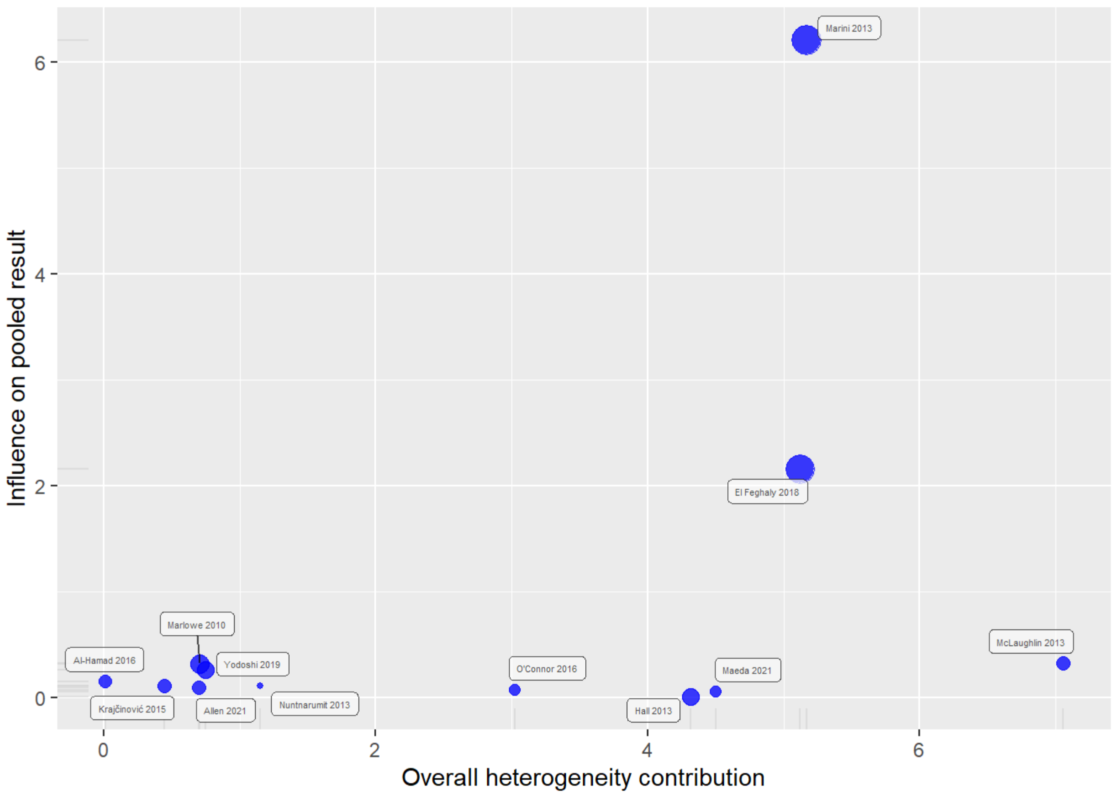

Compute pre and post BCC rates

We want to see the distribution of studies that had //<3%, //<2% and //<1% BCC rates at baseline, and then we want to compare this to post BCC rates. This is important for two reasons: first because there are target rates and we would like to know how many facilities were able to achieve target BCC rates. Second, we will want to know whether pressure for change (operationalized in terms of the baseline BCC rate) is associated with level of improvement in the rate following the intervention.

We will use the combined units data for a facility-wide estimate of baseline and post-intervention BCC rates.

```
BCC_Outcomes1a <- BCC_Outcomes1a %>%
  mutate(Baseline_BCC = Ec/Nc)

BCC_Outcomes1a <- BCC_Outcomes1a %>%
  mutate(Followup_BCC = Ee/Ne)
```

Now, we want to count how many facilities fell into each of the categories.

```
count3B <- sum(BCC_Outcomes1a$Baseline_BCC < 0.03)
count3B
```

```
## [1] 23
```

```
count2B <- sum(BCC_Outcomes1a$Baseline_BCC < 0.02)
count2B
```

```
## [1] 10
```

```
count1B <- sum(BCC_Outcomes1a$Baseline_BCC < 0.01)
count1B
```

```
## [1] 2
```

```
count3F <- sum(BCC_Outcomes1a$Followup_BCC < 0.03)
count3F
```

```
## [1] 41
```

```
count2F <- sum(BCC_Outcomes1a$Followup_BCC < 0.02)
count2F
```

```
## [1] 34
```

```
count1F <- sum(BCC_Outcomes1a$Followup_BCC < 0.01)
count1F
```

```
## [1] 16
```

```
# additional categories for narrative
count3_6B <- sum(BCC_Outcomes1a$Baseline_BCC > 0.03 & BCC_Outcomes1a$Baseline_BCC <= 0.06)
count3_6B
```

```
## [1] 18
```

```
count6B <- sum(BCC_Outcomes1a$Baseline_BCC > 0.06)
count6B
```

```
## [1] 12
```

```
count10B <- sum(BCC_Outcomes1a$Baseline_BCC > 0.1)
count10B
```

```
## [1] 5
```

Next, we’ll compute the percent of sample for each of the rate categories.

```
count3B/56
```

```
## [1] 0.4107143
```

```
count2B/56
```

```
## [1] 0.1785714
```

```
count1B/56
```

```
## [1] 0.03571429
```

```
count3F/56
```

```
## [1] 0.7321429
```

```
count2F/56
```

```
## [1] 0.6071429
```

```
count1F/56
```

```
## [1] 0.2857143
```

```
count3_6B/56
```

```
## [1] 0.3214286
```

```
count6B/56
```

```
## [1] 0.2142857
```

Publication Bias

We will carry out a test for small study (publication) bias as well as generate a funnel plot.

```
metabias(BCC_All1.bin, method.bias = "peters")
```

```
## Linear regression test of funnel plot asymmetry
##
## Test result: t = -0.98, df = 51, p-value = 0.3333
##
## Sample estimates:
##       bias  se.bias intercept se.intercept
##  -229.9839 235.4579   -0.6403       0.0764
##
## Details:
## - multiplicative residual heterogeneity variance (tau^2 = 95.1808)
## - predictor: inverse of total sample size
## - weight:    inverse variance of average event probability
## - reference: Peters et al. (2006), JAMA
```

The test indicates no significant publication bias.

But, we'd like to examine a funnel plot to be sure.

```
# Define fill colors for contour
col.contour = c("gray75", "gray85", "gray95")

# Generate funnel plot again to see the p-values
funnel.meta(BCC_All1.bin, xlim = c(.010, 20.0),
            contour = c(0.9, 0.95, 0.99),
            col.contour = col.contour)

# Add a legend
legend(x = 5, y = 0.01,
       legend = c("p < 0.1", "p < 0.05", "p < 0.01"),
       fill = col.contour)

# Add a title
title("Contour-Enhanced Funnel Plot (Combined Units)")
```

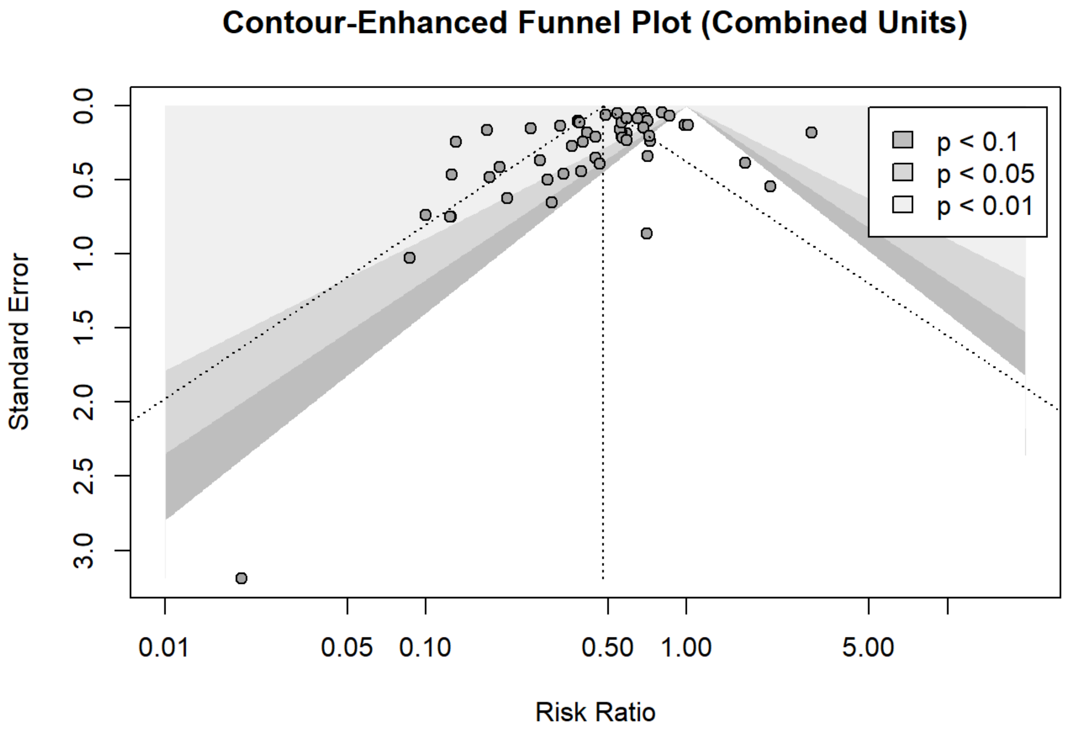

There is some evident asymmetry in the funnel with one small outlier study having a large effecct (Nuntaramumit 2013).

Hypothesis Tests

Hypothesis 1: Baseline BCC rate and outcome

We would expect that a very high BCC rate would result in tension for change. Thus, we should see a positive relationship between the baseline BCC rate and the improvement.

```
BCC_All1a.bin <- metabin(Ee,
                         Ne,
                         Ec,
                         Nc,
                         data = BCC_Outcomes1a, #this is my data set
                         studlab = paste(Author, year),
                         comb.fixed = FALSE,
                         comb.random = TRUE, #here I'm doing a random effects model
                         method.tau = "PM", #I'm going to use a PM first
                         hakn = FALSE,
                         prediction = TRUE,
                         incr = 0.1,
                         sm = "RR")

BCC_All1a.bin
```

```
## Number of studies: k = 53
## Number of observations: o = 958387
## Number of events: e = 18545.67
##
##                          RR           95%-CI     z  p-value
## Random effects model 0.4784 [0.3997; 0.5727] -8.04 < 0.0001
## Prediction interval         [0.1442; 1.5872]
##
## Quantifying heterogeneity:
##  tau^2 = 0.3484 [0.2094; 0.6188]; tau = 0.5902 [0.4576; 0.7867]
##  I^2 = 89.8% [87.4%; 91.7%]; H = 3.13 [2.82; 3.47]
##
## Test of heterogeneity:
##       Q d.f.  p-value
##  508.34   52 < 0.0001
##
## Details on meta-analytical method:
## - Inverse variance method
## - Paule-Mandel estimator for tau^2
## - Q-Profile method for confidence interval of tau^2 and tau
## - Prediction interval based on t-distribution (df = 51)
## - Continuity correction of 0.1 in studies with zero cell frequencies
```

```
m.base.reg <- metareg(BCC_All1a.bin, ~Baseline_BCC)
m.base.reg
```

```
##
## Mixed-Effects Model (k = 53; tau^2 estimator: PM)
##
## tau^2 (estimated amount of residual heterogeneity):     0.3405 (SE = 0.0866)
## tau (square root of estimated tau^2 value):             0.5835
## I^2 (residual heterogeneity / unaccounted variability): 95.48%
## H^2 (unaccounted variability / sampling variability):   22.12
## R^2 (amount of heterogeneity accounted for):            2.27%
##
## Test for Residual Heterogeneity:
## QE(df = 51) = 469.7300, p-val < .0001
##
## Test of Moderators (coefficient 2):
## QM(df = 1) = 1.8564, p-val = 0.1730
##
## Model Results:
##
##               estimate      se     zval    pval    ci.lb    ci.ub
## intrcpt        -0.6038  0.1329  -4.5419  <.0001  -0.8644  -0.3433  ***
## Baseline_BCC   -2.7044  1.9849  -1.3625  0.1730  -6.5947   1.1859
##
## ---
## Signif. codes:  0 '***' 0.001 '**' 0.01 '*' 0.05 '.' 0.1 ' ' 1
```

```
baseline.plot <- regplot(m.base.reg,
        mod="Baseline_BCC",
        pi=TRUE,
        xlab="Baseline BCC",
        xlim=c(0,0.3),
        ylim=c(-.1,3.5),
        predlim=c(0,1),
        transf=exp,
        refline=1,
        legend=TRUE,
        label="piout",
        labsize=0.8)
```

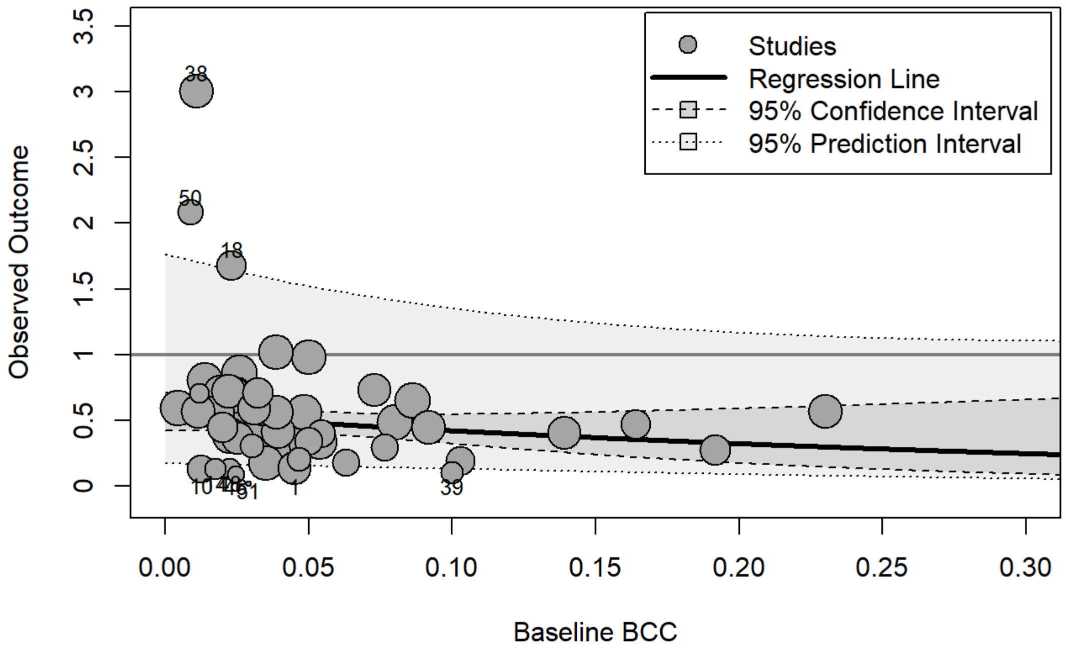

Now the problem with the above is that units within a facility can have very diffrent baseline BCC rates. So, the effect of this tension to change may be felt more in some units than others.

So, we will carry out the same analysis, but this time on a unit level.

This means that we need to create baseline and follow-up variables for the data by unit.

```
BCC_Outcomes <- BCC_Outcomes %>%
  mutate(Baseline_BCC = Ec/Nc)

BCC_Outcomes <- BCC_Outcomes %>%
  mutate(Followup_BCC = Ee/Ne)
```

Now, we will carry out the same meta-analysis. The results will not be particularly meaningful because we have multiple arms per study. However, the per unit analysis should still hold.

```
BCC_All.bin <- metabin(Ee,
                         Ne,
                         Ec,
                         Nc,
                         data = BCC_Outcomes, #this is my data set
                         studlab = paste(Author, year),
                         comb.fixed = FALSE,
                         comb.random = TRUE, #here I'm doing a random effects model
                         method.tau = "PM", #I'm going to use a PM first
                         hakn = FALSE,
                         prediction = TRUE,
                         incr = 0.1,
                         sm = "RR")

BCC_All.bin
```

```
## Number of studies: k = 65
## Number of observations: o = 1168271
## Number of events: e = 20415.67
##
##                           RR           95%-CI     z  p-value
## Random effects model 0.5179 [0.4413; 0.6078] -8.06 < 0.0001
## Prediction interval         [0.1608; 1.6679]
##
## Quantifying heterogeneity:
##  tau^2 = 0.3358 [0.2052; 0.5782]; tau = 0.5795 [0.4530; 0.7604]
##  I^2 = 88.0% [85.4%; 90.1%]; H = 2.89 [2.62; 3.18]
##
## Test of heterogeneity:
##       Q d.f.  p-value
##  533.40   64 < 0.0001
##
## Details on meta-analytical method:
## - Inverse variance method
## - Paule-Mandel estimator for tau^2
## - Q-Profile method for confidence interval of tau^2 and tau
## - Prediction interval based on t-distribution (df = 63)
## - Continuity correction of 0.1 in studies with zero cell frequencies
```

```
m.base.reg.unit <- metareg(BCC_All.bin, ~Baseline_BCC)
```

```
## Warning: 1 study with NAs omitted from model fitting.
```

```
m.base.reg.unit
```

```
##
## Mixed-Effects Model (k = 65; tau^2 estimator: PM)
##
## tau^2 (estimated amount of residual heterogeneity):     0.3283 (SE = 0.0758)
## tau (square root of estimated tau^2 value):             0.5730
## I^2 (residual heterogeneity / unaccounted variability): 95.00%
## H^2 (unaccounted variability / sampling variability):   20.02
## R^2 (amount of heterogeneity accounted for):            2.23%
##
## Test for Residual Heterogeneity:
## QE(df = 63) = 501.3673, p-val < .0001
##
## Test of Moderators (coefficient 2):
## QM(df = 1) = 1.9761, p-val = 0.1598
##
## Model Results:
##
##               estimate      se     zval    pval    ci.lb    ci.ub
## intrcpt        -0.5377  0.1174  -4.5806  <.0001  -0.7678  -0.3076  ***
## Baseline_BCC   -2.5209  1.7933  -1.4057  0.1598  -6.0358   0.9939
##
## ---
## Signif. codes:  0 '***' 0.001 '**' 0.01 '*' 0.05 '.' 0.1 ' ' 1
```

```
baseline.plot <- regplot(m.base.reg.unit,
        mod="Baseline_BCC",
        pi=TRUE,
        xlab="Baseline BCC",
        xlim=c(0,0.3),
        ylim=c(-.1,3.5),
        predlim=c(0,1),
        transf=exp,
        refline=1,
        legend=TRUE,
        label="piout",
        labsize=0.8)
```

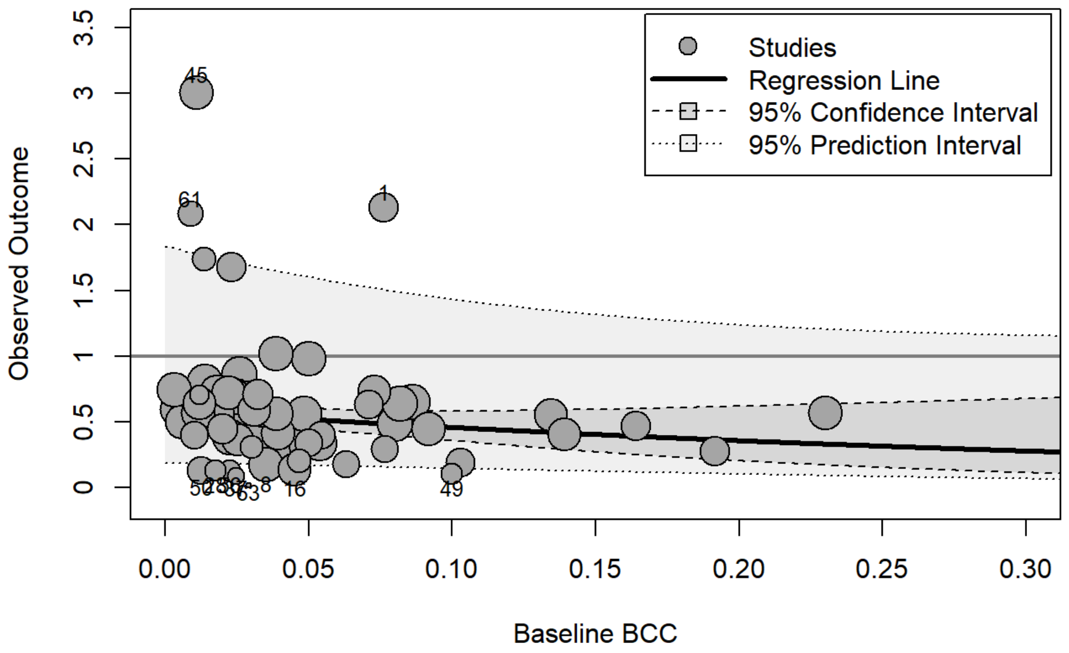

Hypothesis 2: BCC reduction rate will be higher when the implementation is part of a larger QMQI effort

```
Hyp.2.bin <- update.meta(BCC_All1a.bin,
                         subgroup = QMQA,
                         tau.common = TRUE)

Hyp.2.bin
```

```
## Number of studies: k = 53
## Number of observations: o = 958387
## Number of events: e = 18545.67
##
##                          RR           95%-CI     z  p-value
## Random effects model 0.4784 [0.3997; 0.5727] -8.04 < 0.0001
## Prediction interval         [0.1442; 1.5872]
##
## Quantifying heterogeneity:
##  tau^2 = 0.3484 [0.2094; 0.6188]; tau = 0.5902 [0.4576; 0.7867]
##  I^2 = 89.8% [87.4%; 91.7%]; H = 3.13 [2.82; 3.47]
##
## Quantifying residual heterogeneity:
##  tau^2 = 0.3435; tau = 0.5861; I^2 = 89.8% [87.4%; 91.7%]; H = 3.13 [2.82; 3.47]
##
## Test of heterogeneity:
##       Q d.f.  p-value
##  508.34   52 < 0.0001
##
## Results for subgroups (random effects model):
##              k     RR           95%-CI  tau^2    tau      Q   I^2
## QMQA = Yes  35 0.4449 [0.3594; 0.5506] 0.3435 0.5861 374.12 90.9%
## QMQA = No   18 0.5693 [0.4104; 0.7899] 0.3435 0.5861 124.20 86.3%
##
## Test for subgroup differences (random effects model):
##                    Q d.f.  p-value
## Between groups   1.53    1   0.2160
## Within groups  498.33   51 < 0.0001
##
## Details on meta-analytical method:
## - Inverse variance method
## - Paule-Mandel estimator for tau^2
##   (assuming common tau^2 in subgroups)
## - Q-Profile method for confidence interval of tau^2 and tau
## - Prediction interval based on t-distribution (df = 51)
## - Continuity correction of 0.1 in studies with zero cell frequencies
```

```
png(file = "BCC_ALL_QMQA 2-9-24.png", width = 2800, height = 4800, res = 300)
forest(Hyp.2.bin, sortvar = TE, xlab = "Favors Innovation -- Favors Usual")
dev.off()
```

```
## png
##   2
```

```
knitr::include_graphics("BCC_ALL_QMQA 2-9-24.png")
```

Study	Experimental Events	Experimental Total	Control Events	Control Total	Risk Ratio	RR	95%-CI	Weight
**QMQA = Yes**								
Sweet 2012	1	470	17	692		0.09	[0.01; 0.65]	0.6%
Rupp 2017	2	904	16	904		0.12	[0.03; 0.54]	0.9%
He 2020	19	3228	190	4208		0.13	[0.08; 0.21]	2.1%
Bell 2018	38	6293	1246	35392		0.17	[0.12; 0.24]	2.2%
HalsteadD 2020	6	303	154	1497		0.19	[0.09; 0.43]	1.6%
O'Connor 2016	3	314	17	364		0.20	[0.06; 0.69]	1.1%
HalsteadA 2020	44	4604	959	25315		0.25	[0.19; 0.34]	2.3%
Al-Hamad 2019	5	222	17	222		0.29	[0.11; 0.78]	1.4%
Moeller 2017	74	4200	226	4200		0.33	[0.25; 0.42]	2.3%
Lalezari 2020	6	356	20	400		0.34	[0.14; 0.83]	1.5%
Self2 2014	17	1860	63	2509		0.36	[0.21; 0.62]	2.0%
Kamboj 2015	146	12048	342	10778		0.38	[0.31; 0.46]	2.4%
HalsteadB 2020	117	13557	333	14764		0.38	[0.31; 0.47]	2.3%
Self 2013	111	6590	321	7389		0.39	[0.31; 0.48]	2.3%
Zimmerman 2019	7	325	17	312		0.40	[0.17; 0.94]	1.5%
Sanchez 2018	47	841	22	158		0.40	[0.25; 0.65]	2.1%
Hall 2013	35	2153	212	5402		0.41	[0.29; 0.59]	2.2%
Thomas 2011	29	705	90	981		0.45	[0.30; 0.67]	2.1%
Allen 2021	10	1110	39	1944		0.45	[0.23; 0.90]	1.8%
Shaheen 2020	380	9756	709	8868		0.49	[0.43; 0.55]	2.4%
El Feghaly 2018	578	37500	1603	56250		0.54	[0.49; 0.59]	2.4%
Harding 2013	64	6336	101	5544		0.55	[0.41; 0.76]	2.3%
Self1 2014	142	5238	165	3417		0.56	[0.45; 0.70]	2.3%
Patton 2010	58	2672	41	1061		0.56	[0.38; 0.83]	2.2%
Ramirez 2015	40	308	80	348		0.56	[0.40; 0.80]	2.2%
Ryan 2017	21	1152	130	4197		0.59	[0.37; 0.93]	2.1%
Lin 2012	31	1548	328	9645		0.59	[0.41; 0.85]	2.2%
Al-Hamad 2016	231	4138	389	4521		0.65	[0.55; 0.76]	2.4%
Marini 2013	840	60000	1260	60000		0.67	[0.61; 0.73]	2.4%
Santos 2018	2	234	4	329		0.70	[0.13; 3.81]	0.8%
HalsteadC 2020	172	12507	215	11100		0.71	[0.58; 0.87]	2.3%
Park 2015	922	83861	1212	88476		0.80	[0.74; 0.87]	2.4%
Roth 2010	278	12459	1006	38805		0.86	[0.75; 0.98]	2.4%
Murillo 2011	124	2531	114	2280		0.98	[0.76; 1.26]	2.3%
Story-Roller 2016	123	3130	115	2965		1.01	[0.79; 1.30]	2.3%
**Random effects model**		303453		415237		0.44	[0.36; 0.55]	70.2%
Heterogeneity: $I^2 = 91\%$, $\tau^2 = 0.3435$, $p < 0.01$								
**QMQA = No**								
Nuntnarumit 2013	0	172	5	172		0.02	[0.00; 10.21]	0.1%
Frota 2021	2	200	20	200		0.10	[0.02; 0.42]	0.9%
Maeda 2021	2	711	17	749		0.12	[0.03; 0.53]	0.9%
Ge 2011	5	3177	77	6144		0.13	[0.05; 0.31]	1.5%
Kai 2020	5	450	29	460		0.18	[0.07; 0.45]	1.5%
Levin 2013	9	172	27	141		0.27	[0.13; 0.56]	1.7%
Tarrand 2012	3	326	10	331		0.30	[0.08; 1.10]	1.1%
Krajčinović 2015	8	105	22	134		0.46	[0.22; 1.00]	1.7%
Kim 2011	34	5265	60	5255		0.57	[0.37; 0.86]	2.1%
Bae 2019	222	83534	354	78673		0.59	[0.50; 0.70]	2.4%
Marlowe 2010	72	4274	122	4942		0.68	[0.51; 0.91]	2.3%
Syed 2020	239	14046	326	13350		0.70	[0.59; 0.82]	2.4%
Tangsathapompong 2014	14	615	21	654		0.71	[0.36; 1.38]	1.8%
Yodoshi 2019	38	2407	65	2958		0.72	[0.48; 1.07]	2.2%
Yu 2020	29	549	40	549		0.72	[0.46; 1.15]	2.1%
McLaughlin 2013	11	284	16	693		1.68	[0.79; 3.57]	1.7%
Martínez 2017	10	539	5	563		2.09	[0.72; 6.07]	1.3%
Tarai 2012	83	2553	47	4350		3.01	[2.11; 4.29]	2.2%
**Random effects model**		119379		120318		0.57	[0.41; 0.79]	29.8%

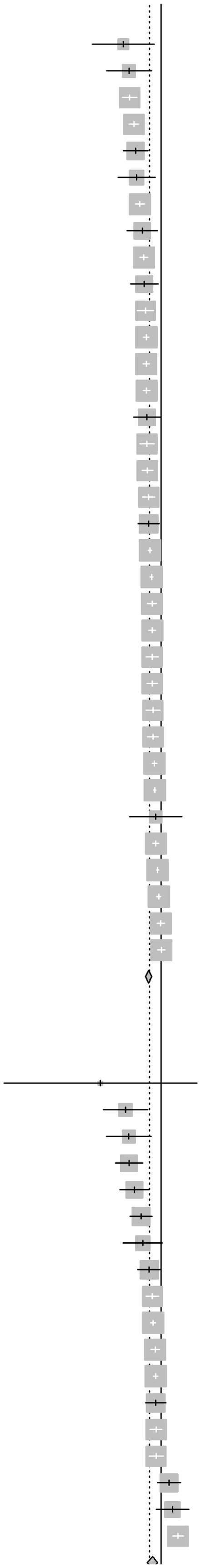

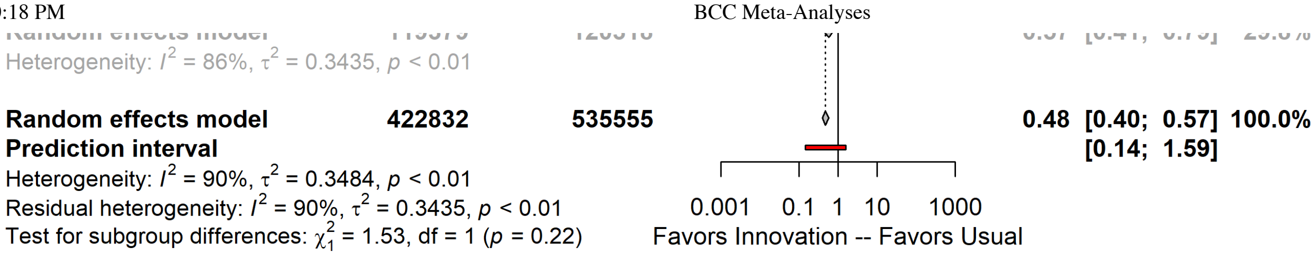


```
m.qmqi.reg <- metareg(BCC_All1a.bin, ~QMQA)
m.qmqi.reg
```

```
##
## Mixed-Effects Model (k = 53; tau^2 estimator: PM)
##
## tau^2 (estimated amount of residual heterogeneity):     0.3435 (SE = 0.0872)
## tau (square root of estimated tau^2 value):             0.5861
## I^2 (residual heterogeneity / unaccounted variability): 95.54%
## H^2 (unaccounted variability / sampling variability):   22.43
## R^2 (amount of heterogeneity accounted for):            1.42%
##
## Test for Residual Heterogeneity:
## QE(df = 51) = 498.3253, p-val < .0001
##
## Test of Moderators (coefficient 2):
## QM(df = 1) = 1.5308, p-val = 0.2160
##
## Model Results:
##
##          estimate      se     zval    pval    ci.lb    ci.ub
## intrcpt   -0.5633  0.1671  -3.3718  0.0007  -0.8907  -0.2359  ***
## QMQAYes   -0.2467  0.1994  -1.2373  0.2160  -0.6374   0.1441
##
## ---
## Signif. codes:  0 '***' 0.001 '**' 0.01 '*' 0.05 '.' 0.1 ' ' 1
```

Count the number of studies that used QMQI efforts.

```
count_QMQA <- sum(BCC_Outcomes1a$QMQA == "Yes")
count_QMQA
```

```
## [1] 35
```

Hypothesis 3: Reduction of BCC rate is improved with greater training intensity

```
Hyp.3d.bin <- update.meta(BCC_All1a.bin,
                         subgroup = Training_dich,
                         tau.common = TRUE)

Hyp.3d.bin
```

```
## Number of studies: k = 53
## Number of observations: o = 958387
## Number of events: e = 18545.67
##
##                           RR           95%-CI     z  p-value
## Random effects model 0.4784 [0.3997; 0.5727] -8.04 < 0.0001
## Prediction interval         [0.1442; 1.5872]
##
## Quantifying heterogeneity:
##  tau^2 = 0.3484 [0.2094; 0.6188]; tau = 0.5902 [0.4576; 0.7867]
##  I^2 = 89.8% [87.4%; 91.7%]; H = 3.13 [2.82; 3.47]
##
## Quantifying residual heterogeneity:
##  tau^2 = 0.3320; tau = 0.5762; I^2 = 89.9% [87.6%; 91.8%]; H = 3.14 [2.83; 3.49]
##
## Test of heterogeneity:
##       Q d.f.  p-value
##  508.34   52 < 0.0001
##
## Results for subgroups (random effects model):
##                         k     RR           95%-CI  tau^2    tau      Q   I^2
## Training_dich = High  33 0.4318 [0.3484; 0.5351] 0.3320 0.5762 356.98 91.0%
## Training_dich = Low   20 0.5957 [0.4376; 0.8110] 0.3320 0.5762 147.00 87.1%
##
## Test for subgroup differences (random effects model):
##                     Q d.f.  p-value
## Between groups   2.82    1   0.0932
## Within groups  503.99   51 < 0.0001
##
## Details on meta-analytical method:
## - Inverse variance method
## - Paule-Mandel estimator for tau^2
##   (assuming common tau^2 in subgroups)
## - Q-Profile method for confidence interval of tau^2 and tau
## - Prediction interval based on t-distribution (df = 51)
## - Continuity correction of 0.1 in studies with zero cell frequencies
```

```
png(file = "BCC_ALL_train 2-9-24.png", width = 2800, height = 4800, res = 300)
forest(Hyp.3d.bin, sortvar = TE, xlab = "Favors Innovation -- Favors Usual")
dev.off()
```

```
## png
##   2
```

```
knitr::include_graphics("BCC_ALL_train 2-9-24.png")
```

Study	Experimental Events	Experimental Total	Control Events	Control Total	RR	95%-CI	Weight
**Training_dich = High**							
Frota 2021	2	200	20	200	0.10	[0.02; 0.42]	0.9%
Maeda 2021	2	711	17	749	0.12	[0.03; 0.53]	0.9%
Ge 2011	5	3177	77	6144	0.13	[0.05; 0.31]	1.5%
He 2020	19	3228	190	4208	0.13	[0.08; 0.21]	2.1%
Bell 2018	38	6293	1246	35392	0.17	[0.12; 0.24]	2.2%
Kai 2020	5	450	29	460	0.18	[0.07; 0.45]	1.5%
HalsteadD 2020	6	303	154	1497	0.19	[0.09; 0.43]	1.6%
O'Connor 2016	3	314	17	364	0.20	[0.06; 0.69]	1.1%
HalsteadA 2020	44	4604	959	25315	0.25	[0.19; 0.34]	2.3%
Al-Hamad 2019	5	222	17	222	0.29	[0.11; 0.78]	1.4%
Self2 2014	17	1860	63	2509	0.36	[0.21; 0.62]	2.0%
Kamboj 2015	146	12048	342	10778	0.38	[0.31; 0.46]	2.4%
HalsteadB 2020	117	13557	333	14764	0.38	[0.31; 0.47]	2.3%
Self 2013	111	6590	321	7389	0.39	[0.31; 0.48]	2.3%
Sanchez 2018	47	841	22	158	0.40	[0.25; 0.65]	2.1%
Hall 2013	35	2153	212	5402	0.41	[0.29; 0.59]	2.2%
Allen 2021	10	1110	39	1944	0.45	[0.23; 0.90]	1.8%
Shaheen 2020	380	9756	709	8868	0.49	[0.43; 0.55]	2.4%
El Feghaly 2018	578	37500	1603	56250	0.54	[0.49; 0.59]	2.4%
Harding 2013	64	6336	101	5544	0.55	[0.41; 0.76]	2.3%
Self1 2014	142	5238	165	3417	0.56	[0.45; 0.70]	2.3%
Kim 2011	34	5265	60	5255	0.57	[0.37; 0.86]	2.1%
Lin 2012	31	1548	328	9645	0.59	[0.41; 0.85]	2.2%
Bae 2019	222	83534	354	78673	0.59	[0.50; 0.70]	2.4%
Al-Hamad 2016	231	4138	389	4521	0.65	[0.55; 0.76]	2.4%
Marini 2013	840	60000	1260	60000	0.67	[0.61; 0.73]	2.4%
Syed 2020	239	14046	326	13350	0.70	[0.59; 0.82]	2.4%
Tangsathapompong 2014	14	615	21	654	0.71	[0.36; 1.38]	1.8%
HalsteadC 2020	172	12507	215	11100	0.71	[0.58; 0.87]	2.3%
Yodoshi 2019	38	2407	65	2958	0.72	[0.48; 1.07]	2.2%
Park 2015	922	83861	1212	88476	0.80	[0.74; 0.87]	2.4%
Roth 2010	278	12459	1006	38805	0.86	[0.75; 0.98]	2.4%
Murillo 2011	124	2531	114	2280	0.98	[0.76; 1.26]	2.3%
**Random effects model**		**399402**		**507291**	**0.43**	**[0.35; 0.54]**	**67.3%**
Heterogeneity: $I^2 = 91\%$, $\tau^2 = 0.3320$, $p < 0.01$							
**Training_dich = Low**							
Nuntnarumit 2013	0	172	5	172	0.02	[0.00; 10.21]	0.1%
Sweet 2012	1	470	17	692	0.09	[0.01; 0.65]	0.6%
Rupp 2017	2	904	16	904	0.12	[0.03; 0.54]	0.9%
Levin 2013	9	172	27	141	0.27	[0.13; 0.56]	1.7%
Tarrand 2012	3	326	10	331	0.30	[0.08; 1.10]	1.1%
Moeller 2017	74	4200	226	4200	0.33	[0.25; 0.42]	2.3%
Lalezari 2020	6	356	20	400	0.34	[0.14; 0.83]	1.5%
Zimmerman 2019	7	325	17	312	0.40	[0.17; 0.94]	1.5%
Thomas 2011	29	705	90	981	0.45	[0.30; 0.67]	2.1%
Krajčinović 2015	8	105	22	134	0.46	[0.22; 1.00]	1.7%
Patton 2010	58	2672	41	1061	0.56	[0.38; 0.83]	2.2%
Ramirez 2015	40	308	80	348	0.56	[0.40; 0.80]	2.2%
Ryan 2017	21	1152	130	4197	0.59	[0.37; 0.93]	2.1%
Marlowe 2010	72	4274	122	4942	0.68	[0.51; 0.91]	2.3%
Santos 2018	2	234	4	329	0.70	[0.13; 3.81]	0.8%
Yu 2020	29	549	40	549	0.72	[0.46; 1.15]	2.1%
Story-Roller 2016	123	3130	115	2965	1.01	[0.79; 1.30]	2.3%
McLaughlin 2013	11	284	16	693	1.68	[0.79; 3.57]	1.7%
Martínez 2017	10	539	5	563	2.09	[0.72; 6.07]	1.3%
Tarai 2012	83	2553	47	4350	3.01	[2.11; 4.29]	2.2%
**Random effects model**		**23430**		**28264**	**0.60**	**[0.44; 0.81]**	**32.7%**

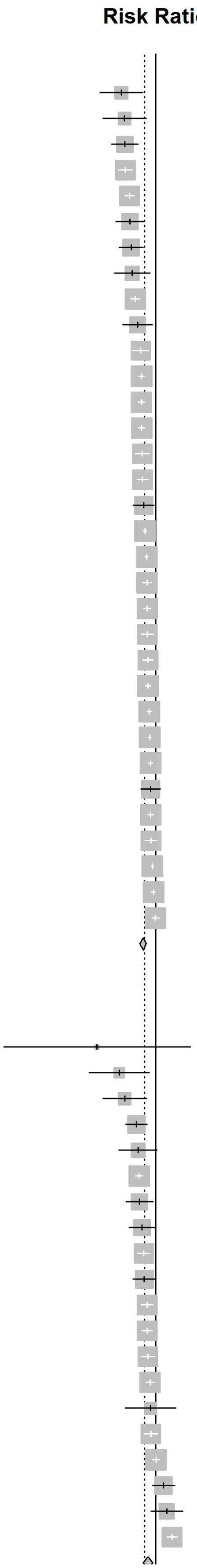

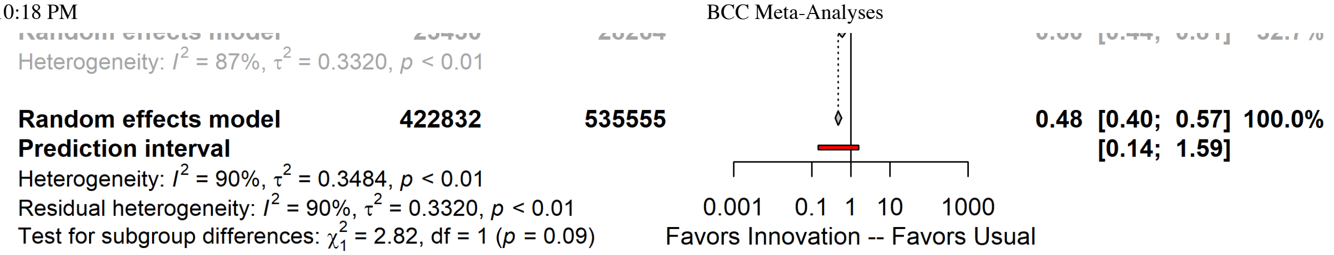


```
m.qmqi.reg <- metareg(BCC_All1a.bin, ~Training_dich)
m.qmqi.reg
```

```
##
## Mixed-Effects Model (k = 53; tau^2 estimator: PM)
##
## tau^2 (estimated amount of residual heterogeneity):     0.3320 (SE = 0.0848)
## tau (square root of estimated tau^2 value):             0.5762
## I^2 (residual heterogeneity / unaccounted variability): 95.47%
## H^2 (unaccounted variability / sampling variability):   22.08
## R^2 (amount of heterogeneity accounted for):            4.71%
##
## Test for Residual Heterogeneity:
## QE(df = 51) = 503.9857, p-val < .0001
##
## Test of Moderators (coefficient 2):
## QM(df = 1) = 2.8178, p-val = 0.0932
##
## Model Results:
##
##                    estimate      se     zval    pval    ci.lb    ci.ub
## intrcpt             -0.8398  0.1095  -7.6709  <.0001  -1.0543  -0.6252  ***
## Training_dichLow     0.3218  0.1917   1.6786  0.0932  -0.0539   0.6976    .
##
## ---
## Signif. codes:  0 '***' 0.001 '**' 0.01 '*' 0.05 '.' 0.1 ' ' 1
```

Combined QMQA and High Intensity Training

Including QMQA or high intensity training brings about greater reduction in BCC rates than interventions that do not, but is there a synergistic effect? Do organizations that combine both reduce rates more than organizations that use only one or the other or do neither?

We've created two new variables to get at this variation:

1. comb_QAQI_intense, which has three values: Both, One, or Neither.
2. Hyp2_3, which has four values: Both, QMQA Only, Intense Training Only, Neither

Because these are not dichotomous variables, I don't believe the standard update_meta command will accurately test for group differences. For this, we'd need a random effects model with dummy covariates (two or three depending on the variable). Possible, but given that the number of studies that utilize neither is small and so we are already underpowered for this test. So, I'm not sure it's worth doing.

First, let's just see if there are any meaningful differences between groups with the two variables.

```
Comb.Hyp.2.3_3cat <- update.meta(BCC_All1a.bin,
                         subgroup = comb_QAQI_intense,
                         tau.common = TRUE)

Comb.Hyp.2.3_3cat
```

```
## Number of studies: k = 53
## Number of observations: o = 958387
## Number of events: e = 18545.67
##
##                          RR           95%-CI     z  p-value
## Random effects model 0.4784 [0.3997; 0.5727] -8.04 < 0.0001
## Prediction interval         [0.1442; 1.5872]
##
## Quantifying heterogeneity:
##  tau^2 = 0.3484 [0.2094; 0.6188]; tau = 0.5902 [0.4576; 0.7867]
##  I^2 = 89.8% [87.4%; 91.7%]; H = 3.13 [2.82; 3.47]
##
## Quantifying residual heterogeneity:
##  tau^2 = 0.3067; tau = 0.5538; I^2 = 89.5% [87.1%; 91.5%]; H = 3.09 [2.78; 3.43]
##
## Test of heterogeneity:
##       Q d.f.  p-value
##  508.34   52 < 0.0001
##
## Results for subgroups (random effects model):
##                              k     RR           95%-CI  tau^2    tau      Q
## comb_QAQI_intense = Both     24 0.4426 [0.3495; 0.5605] 0.3067 0.5538 324.11
## comb_QAQI_intense = One      20 0.4338 [0.3239; 0.5811] 0.3067 0.5538  82.82
## comb_QAQI_intense = Neither   9 0.8482 [0.5378; 1.3379] 0.3067 0.5538  70.43
##                              I^2
## comb_QAQI_intense = Both    92.9%
## comb_QAQI_intense = One     77.1%
## comb_QAQI_intense = Neither 88.6%
##
## Test for subgroup differences (random effects model):
##                     Q d.f.  p-value
## Between groups   6.91    2   0.0316
## Within groups  477.37   50 < 0.0001
##
## Details on meta-analytical method:
## - Inverse variance method
## - Paule-Mandel estimator for tau^2
##   (assuming common tau^2 in subgroups)
## - Q-Profile method for confidence interval of tau^2 and tau
## - Prediction interval based on t-distribution (df = 51)
## - Continuity correction of 0.1 in studies with zero cell frequencies
```

```
png(file = "Hyp.2.3_3cat 6-21-24.png", width = 2800, height = 4800, res = 300)
forest(Comb.Hyp.2.3_3cat, sortvar = TE, xlab = "Favors Innovation -- Favors Usual")
dev.off()
```

```
## png
##   2
```

```
knitr::include_graphics("Hyp.2.3_3cat 6-21-24.png")
```

Study	Experimental Events	Experimental Total	Control Events	Control Total	RR	95%-CI	Weight
**comb_QAQI_intense = Both**							
He 2020	19	3228	190	4208	0.13	[0.08; 0.21]	2.1%
Bell 2018	38	6293	1246	35392	0.17	[0.12; 0.24]	2.2%
HalsteadD 2020	6	303	154	1497	0.19	[0.09; 0.43]	1.6%
O'Connor 2016	3	314	17	364	0.20	[0.06; 0.69]	1.1%
HalsteadA 2020	44	4604	959	25315	0.25	[0.19; 0.34]	2.3%
Al-Hamad 2019	5	222	17	222	0.29	[0.11; 0.78]	1.4%
Self2 2014	17	1860	63	2509	0.36	[0.21; 0.62]	2.0%
Kamboj 2015	146	12048	342	10778	0.38	[0.31; 0.46]	2.4%
HalsteadB 2020	117	13557	333	14764	0.38	[0.31; 0.47]	2.3%
Self 2013	111	6590	321	7389	0.39	[0.31; 0.48]	2.3%
Sanchez 2018	47	841	22	158	0.40	[0.25; 0.65]	2.1%
Hall 2013	35	2153	212	5402	0.41	[0.29; 0.59]	2.2%
Allen 2021	10	1110	39	1944	0.45	[0.23; 0.90]	1.8%
Shaheen 2020	380	9756	709	8868	0.49	[0.43; 0.55]	2.4%
El Feghaly 2018	578	37500	1603	56250	0.54	[0.49; 0.59]	2.4%
Harding 2013	64	6336	101	5544	0.55	[0.41; 0.76]	2.3%
Self1 2014	142	5238	165	3417	0.56	[0.45; 0.70]	2.3%
Lin 2012	31	1548	328	9645	0.59	[0.41; 0.85]	2.2%
Al-Hamad 2016	231	4138	389	4521	0.65	[0.55; 0.76]	2.4%
Marini 2013	840	60000	1260	60000	0.67	[0.61; 0.73]	2.4%
HalsteadC 2020	172	12507	215	11100	0.71	[0.58; 0.87]	2.3%
Park 2015	922	83861	1212	88476	0.80	[0.74; 0.87]	2.4%
Roth 2010	278	12459	1006	38805	0.86	[0.75; 0.98]	2.4%
Murillo 2011	124	2531	114	2280	0.98	[0.76; 1.26]	2.3%
**Random effects model**		**288997**		**398848**	**0.44**	**[0.35; 0.56]**	**51.6%**
Heterogeneity: $I^2 = 93\%$, $\tau^2 = 0.3067$, $p < 0.01$							
**comb_QAQI_intense = One**							
Sweet 2012	1	470	17	692	0.09	[0.01; 0.65]	0.6%
Frota 2021	2	200	20	200	0.10	[0.02; 0.42]	0.9%
Maeda 2021	2	711	17	749	0.12	[0.03; 0.53]	0.9%
Rupp 2017	2	904	16	904	0.12	[0.03; 0.54]	0.9%
Ge 2011	5	3177	77	6144	0.13	[0.05; 0.31]	1.5%
Kai 2020	5	450	29	460	0.18	[0.07; 0.45]	1.5%
Moeller 2017	74	4200	226	4200	0.33	[0.25; 0.42]	2.3%
Lalezari 2020	6	356	20	400	0.34	[0.14; 0.83]	1.5%
Zimmerman 2019	7	325	17	312	0.40	[0.17; 0.94]	1.5%
Thomas 2011	29	705	90	981	0.45	[0.30; 0.67]	2.1%
Patton 2010	58	2672	41	1061	0.56	[0.38; 0.83]	2.2%
Ramirez 2015	40	308	80	348	0.56	[0.40; 0.80]	2.2%
Kim 2011	34	5265	60	5255	0.57	[0.37; 0.86]	2.1%
Ryan 2017	21	1152	130	4197	0.59	[0.37; 0.93]	2.1%
Bae 2019	222	83534	354	78673	0.59	[0.50; 0.70]	2.4%
Syed 2020	239	14046	326	13350	0.70	[0.59; 0.82]	2.4%
Santos 2018	2	234	4	329	0.70	[0.13; 3.81]	0.8%
Tangsathapompong 2014	14	615	21	654	0.71	[0.36; 1.38]	1.8%
Yodoshi 2019	38	2407	65	2958	0.72	[0.48; 1.07]	2.2%
Story-Roller 2016	123	3130	115	2965	1.01	[0.79; 1.30]	2.3%
**Random effects model**		**124861**		**124832**	**0.43**	**[0.32; 0.58]**	**34.2%**
Heterogeneity: $I^2 = 77\%$, $\tau^2 = 0.3067$, $p < 0.01$							
**comb_QAQI_intense = Neither**							
Nuntnarumit 2013	0	172	5	172	0.02	[0.00; 10.21]	0.1%
Levin 2013	9	172	27	141	0.27	[0.13; 0.56]	1.7%
Tarrand 2012	3	326	10	331	0.30	[0.08; 1.10]	1.1%
Krajčinović 2015	8	105	22	134	0.46	[0.22; 1.00]	1.7%
Marlowe 2010	72	4274	122	4942	0.68	[0.51; 0.91]	2.3%
Yu 2020	29	549	40	549	0.72	[0.46; 1.15]	2.1%
McLaughlin 2013	11	284	16	693	1.68	[0.79; 3.57]	1.7%
Martínez 2017	10	539	5	563	2.09	[0.72; 6.07]	1.3%

Risk Ratio

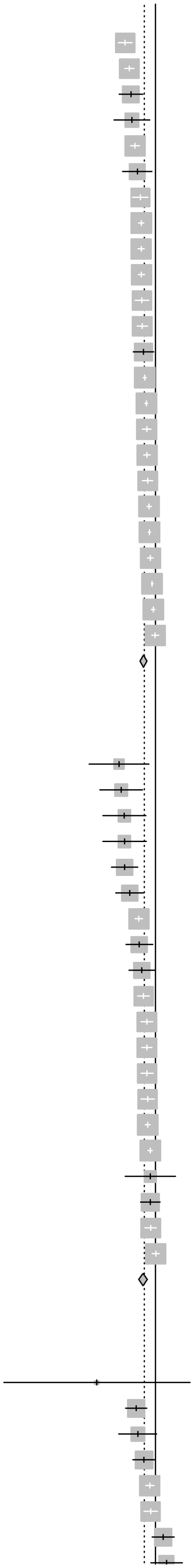

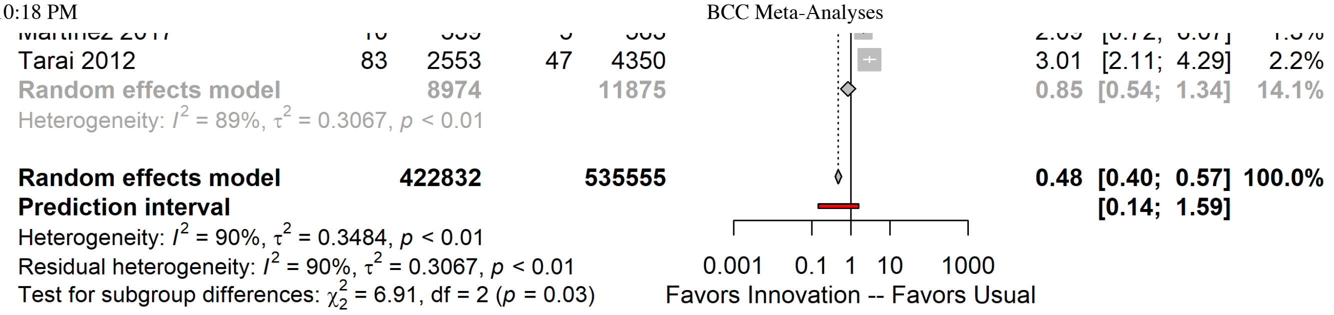

Interpretation: The effects of using one or both were nearly identical, but significantly different (p=0.0316) from interventions that used neither approach to enhancing the effectiveness of the BCC reduction intervention. This is a much cleaner picture of the effects of the QMQA and intensive training approaches than the previous hypothesis tests where we looked at each in isolation.

Now, we will test the four category variable.

```
Comb.Hyp.2.3_4cat <- update.meta(BCC_All1a.bin,
                         subgroup = Hyp2_3,
                         tau.common = TRUE)

Comb.Hyp.2.3_4cat
```

```
## Number of studies: k = 53
## Number of observations: o = 958387
## Number of events: e = 18545.67
##
##                           RR           95%-CI     z  p-value
## Random effects model 0.4784 [0.3997; 0.5727] -8.04 < 0.0001
## Prediction interval         [0.1442; 1.5872]
##
## Quantifying heterogeneity:
##  tau^2 = 0.3484 [0.2094; 0.6188]; tau = 0.5902 [0.4576; 0.7867]
##  I^2 = 89.8% [87.4%; 91.7%]; H = 3.13 [2.82; 3.47]
##
## Quantifying residual heterogeneity:
##  tau^2 = 0.3140; tau = 0.5604; I^2 = 89.7% [87.3%; 91.7%]; H = 3.12 [2.80; 3.46]
##
## Test of heterogeneity:
##       Q d.f.  p-value
##  508.34   52 < 0.0001
##
## Results for subgroups (random effects model):
##                             k     RR           95%-CI  tau^2    tau      Q   I^2
## Hyp2_3 = Both              24 0.4423 [0.3484; 0.5615] 0.3140 0.5604 324.11 92.9%
## Hyp2_3 = QMQA Only         11 0.4585 [0.3073; 0.6843] 0.3140 0.5604  48.48 79.4%
## Hyp2_3 = High Train Only    9 0.4041 [0.2613; 0.6250] 0.3140 0.5604  32.68 75.5%
## Hyp2_3 = Neither            9 0.8472 [0.5350; 1.3417] 0.3140 0.5604  70.43 88.6%
##
## Test for subgroup differences (random effects model):
##                     Q d.f.  p-value
## Between groups   6.97    3   0.0730
## Within groups  475.71   49 < 0.0001
##
## Details on meta-analytical method:
## - Inverse variance method
## - Paule-Mandel estimator for tau^2
##   (assuming common tau^2 in subgroups)
## - Q-Profile method for confidence interval of tau^2 and tau
## - Prediction interval based on t-distribution (df = 51)
## - Continuity correction of 0.1 in studies with zero cell frequencies
```

```
png(file = "Hyp.2.3_4cat 6-21-24.png", width = 2800, height = 4800, res = 300)
forest(Comb.Hyp.2.3_4cat, sortvar = TE, xlab = "Favors Innovation -- Favors Usual")
dev.off()
```

```
## png
##   2
```

```
knitr::include_graphics("Hyp.2.3_4cat 6-21-24.png")
```

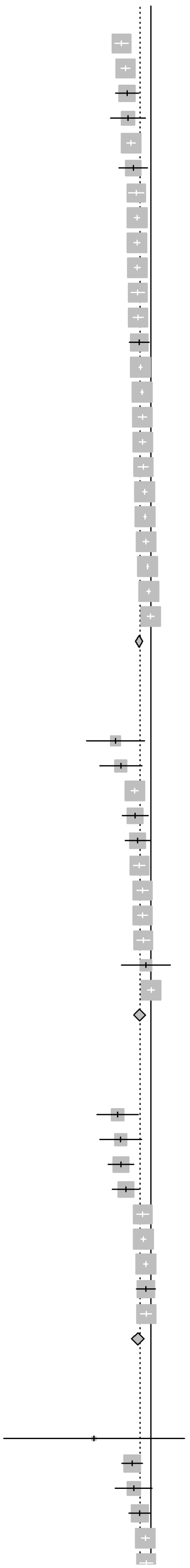

Study	Experimental Events	Experimental Total	Control Events	Control Total	RR	95%-CI	Weight
**Hyp2_3 = Both**							
He 2020	19	3228	190	4208	0.13	[0.08; 0.21]	2.1%
Bell 2018	38	6293	1246	35392	0.17	[0.12; 0.24]	2.2%
HalsteadD 2020	6	303	154	1497	0.19	[0.09; 0.43]	1.6%
O'Connor 2016	3	314	17	364	0.20	[0.06; 0.69]	1.1%
HalsteadA 2020	44	4604	959	25315	0.25	[0.19; 0.34]	2.3%
Al-Hamad 2019	5	222	17	222	0.29	[0.11; 0.78]	1.4%
Self2 2014	17	1860	63	2509	0.36	[0.21; 0.62]	2.0%
Kamboj 2015	146	12048	342	10778	0.38	[0.31; 0.46]	2.4%
HalsteadB 2020	117	13557	333	14764	0.38	[0.31; 0.47]	2.3%
Self 2013	111	6590	321	7389	0.39	[0.31; 0.48]	2.3%
Sanchez 2018	47	841	22	158	0.40	[0.25; 0.65]	2.1%
Hall 2013	35	2153	212	5402	0.41	[0.29; 0.59]	2.2%
Allen 2021	10	1110	39	1944	0.45	[0.23; 0.90]	1.8%
Shaheen 2020	380	9756	709	8868	0.49	[0.43; 0.55]	2.4%
El Feghaly 2018	578	37500	1603	56250	0.54	[0.49; 0.59]	2.4%
Harding 2013	64	6336	101	5544	0.55	[0.41; 0.76]	2.3%
Self1 2014	142	5238	165	3417	0.56	[0.45; 0.70]	2.3%
Lin 2012	31	1548	328	9645	0.59	[0.41; 0.85]	2.2%
Al-Hamad 2016	231	4138	389	4521	0.65	[0.55; 0.76]	2.4%
Marini 2013	840	60000	1260	60000	0.67	[0.61; 0.73]	2.4%
HalsteadC 2020	172	12507	215	11100	0.71	[0.58; 0.87]	2.3%
Park 2015	922	83861	1212	88476	0.80	[0.74; 0.87]	2.4%
Roth 2010	278	12459	1006	38805	0.86	[0.75; 0.98]	2.4%
Murillo 2011	124	2531	114	2280	0.98	[0.76; 1.26]	2.3%
**Random effects model**		**288997**		**398848**	**0.44**	**[0.35; 0.56]**	**51.6%**
Heterogeneity: $I^2 = 93\%$, $\tau^2 = 0.3140$, $p < 0.01$							
**Hyp2_3 = QMQA Only**							
Sweet 2012	1	470	17	692	0.09	[0.01; 0.65]	0.6%
Rupp 2017	2	904	16	904	0.12	[0.03; 0.54]	0.9%
Moeller 2017	74	4200	226	4200	0.33	[0.25; 0.42]	2.3%
Lalezari 2020	6	356	20	400	0.34	[0.14; 0.83]	1.5%
Zimmerman 2019	7	325	17	312	0.40	[0.17; 0.94]	1.5%
Thomas 2011	29	705	90	981	0.45	[0.30; 0.67]	2.1%
Patton 2010	58	2672	41	1061	0.56	[0.38; 0.83]	2.2%
Ramirez 2015	40	308	80	348	0.56	[0.40; 0.80]	2.2%
Ryan 2017	21	1152	130	4197	0.59	[0.37; 0.93]	2.1%
Santos 2018	2	234	4	329	0.70	[0.13; 3.81]	0.8%
Story-Roller 2016	123	3130	115	2965	1.01	[0.79; 1.30]	2.3%
**Random effects model**		**14456**		**16389**	**0.46**	**[0.31; 0.68]**	**18.6%**
Heterogeneity: $I^2 = 79\%$, $\tau^2 = 0.3140$, $p < 0.01$							
**Hyp2_3 = High Train Only**							
Frota 2021	2	200	20	200	0.10	[0.02; 0.42]	0.9%
Maeda 2021	2	711	17	749	0.12	[0.03; 0.53]	0.9%
Ge 2011	5	3177	77	6144	0.13	[0.05; 0.31]	1.5%
Kai 2020	5	450	29	460	0.18	[0.07; 0.45]	1.5%
Kim 2011	34	5265	60	5255	0.57	[0.37; 0.86]	2.1%
Bae 2019	222	83534	354	78673	0.59	[0.50; 0.70]	2.4%
Syed 2020	239	14046	326	13350	0.70	[0.59; 0.82]	2.4%
Tangsathapompong 2014	14	615	21	654	0.71	[0.36; 1.38]	1.8%
Yodoshi 2019	38	2407	65	2958	0.72	[0.48; 1.07]	2.2%
**Random effects model**		**110405**		**108443**	**0.40**	**[0.26; 0.63]**	**15.7%**
Heterogeneity: $I^2 = 76\%$, $\tau^2 = 0.3140$, $p < 0.01$							
**Hyp2_3 = Neither**							
Nuntnarumit 2013	0	172	5	172	0.02	[0.00; 10.21]	0.1%
Levin 2013	9	172	27	141	0.27	[0.13; 0.56]	1.7%
Tarrand 2012	3	326	10	331	0.30	[0.08; 1.10]	1.1%
Krajčinović 2015	8	105	22	134	0.46	[0.22; 1.00]	1.7%
Marlowe 2010	72	4274	122	4942	0.68	[0.51; 0.91]	2.3%
Yu 2020	29	549	40	549	0.72	[0.46; 1.15]	2.1%

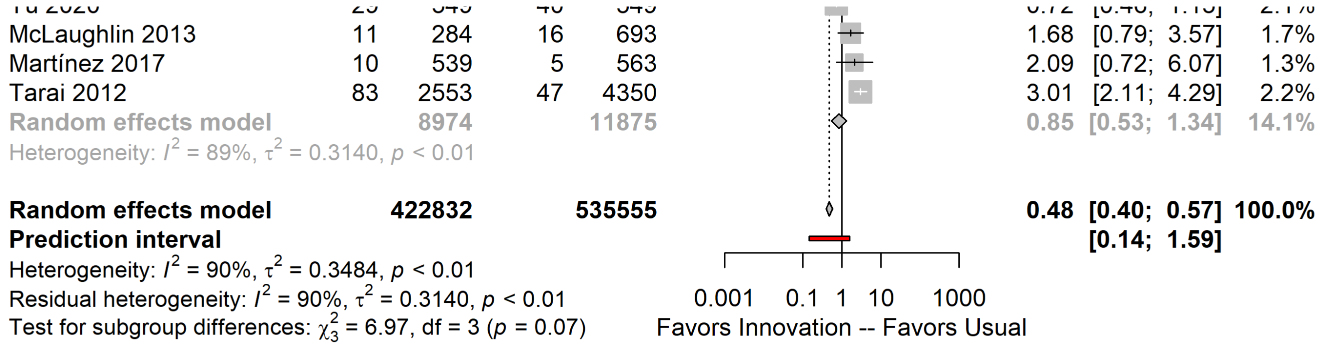

Interpretation: Results are quite similar with either or combined implementation strategies far out performing the organizations that used neither approach to enhancing the effectiveness of the BCC reduction intervention. However, because of the loss of power for this analysis (increased df from creating the fourth small n arm), the between group significance did not quite reach p<.05 (p=0.073).

General Sources of Heterogeneity

Risk of Bias Analysis

We want to determine whether the outcomes, in general, are associated with study ROB. This could influence our interpretation of the findidngs.

```
BCC_all_studies.bin1a <- metabin(Ee,
                                 Ne,
                                 Ec,
                                 Nc,
                                 data = BCC_Outcomes1a, #this is my data set
                                 studlab = paste(Author, year),
                                 comb.fixed = FALSE,
                                 comb.random = TRUE, #here I'm doing a random effects model
                                 method.tau = "PM", #I'm going to use a PM first
                                 hakn = FALSE,
                                 prediction = TRUE,
                                 incr = 0.1,
                                 sm = "RR")

BCC_all_studies.bin1a
```

```
## Number of studies: k = 53
## Number of observations: o = 958387
## Number of events: e = 18545.67
##
##                          RR           95%-CI     z  p-value
## Random effects model 0.4784 [0.3997; 0.5727] -8.04 < 0.0001
## Prediction interval         [0.1442; 1.5872]
##
## Quantifying heterogeneity:
##  tau^2 = 0.3484 [0.2094; 0.6188]; tau = 0.5902 [0.4576; 0.7867]
##  I^2 = 89.8% [87.4%; 91.7%]; H = 3.13 [2.82; 3.47]
##
## Test of heterogeneity:
##       Q d.f.  p-value
##  508.34   52 < 0.0001
##
## Details on meta-analytical method:
## - Inverse variance method
## - Paule-Mandel estimator for tau^2
## - Q-Profile method for confidence interval of tau^2 and tau
## - Prediction interval based on t-distribution (df = 51)
## - Continuity correction of 0.1 in studies with zero cell frequencies
```

```
BCC_all_rob<-update.meta(BCC_all_studies.bin1a,
                      byvar=overall_ROB,
                      comb.random = TRUE,
                      comb.fixed = FALSE,
                      bysort=TRUE)
BCC_all_rob
```

```
## Number of studies: k = 53
## Number of observations: o = 958387
## Number of events: e = 18545.67
##
##                            RR           95%-CI     z  p-value
## Random effects model 0.4784 [0.3997; 0.5727] -8.04 < 0.0001
## Prediction interval         [0.1442; 1.5872]
##
## Quantifying heterogeneity:
##  tau^2 = 0.3484 [0.2094; 0.6188]; tau = 0.5902 [0.4576; 0.7867]
##  I^2 = 89.8% [87.4%; 91.7%]; H = 3.13 [2.82; 3.47]
##
## Test of heterogeneity:
##       Q d.f.  p-value
##  508.34   52 < 0.0001
##
## Results for subgroups (random effects model):
##                            k     RR           95%-CI  tau^2    tau      Q   I^2
## overall_ROB = Low       36 0.4748 [0.3931; 0.5736] 0.2610 0.5109 314.97 88.9%
## overall_ROB = High      14 0.4616 [0.2881; 0.7397] 0.6846 0.8274 159.30 91.8%
## overall_ROB = Moderate   3 0.5199 [0.2479; 1.0906] 0.1756 0.4191   3.36 40.5%
##
## Test for subgroup differences (random effects model):
##                   Q d.f. p-value
## Between groups 0.07    2  0.9650
##
## Details on meta-analytical method:
## - Inverse variance method
## - Paule-Mandel estimator for tau^2
## - Q-Profile method for confidence interval of tau^2 and tau
## - Prediction interval based on t-distribution (df = 51)
## - Continuity correction of 0.1 in studies with zero cell frequencies
```

```
png(file = "BCC_ROB_ALL_3-1-24.png", width = 2800, height = 4800, res = 300)

forest(BCC_all_rob, sortvar = TE, xlab = "Favors Innovation -- Favors Usual")

dev.off()
```

```
## png
##   2
```

```
knitr::include_graphics("BCC_ROB_ALL_3-1-24.png")
```

Study	Experimental Events	Experimental Total	Control Events	Control Total	RR	95%-CI	Weight
**overall_ROB = Low**							
Sweet 2012	1	470	17	692	0.09	[0.01; 0.65]	0.6%
Maeda 2021	2	711	17	749	0.12	[0.03; 0.53]	0.9%
He 2020	19	3228	190	4208	0.13	[0.08; 0.21]	2.1%
Bell 2018	38	6293	1246	35392	0.17	[0.12; 0.24]	2.2%
HalsteadD 2020	6	303	154	1497	0.19	[0.09; 0.43]	1.6%
O'Connor 2016	3	314	17	364	0.20	[0.06; 0.69]	1.1%
HalsteadA 2020	44	4604	959	25315	0.25	[0.19; 0.34]	2.3%
Al-Hamad 2019	5	222	17	222	0.29	[0.11; 0.78]	1.4%
Tarrand 2012	3	326	10	331	0.30	[0.08; 1.10]	1.1%
Moeller 2017	74	4200	226	4200	0.33	[0.25; 0.42]	2.3%
Self2 2014	17	1860	63	2509	0.36	[0.21; 0.62]	2.0%
Kamboj 2015	146	12048	342	10778	0.38	[0.31; 0.46]	2.4%
HalsteadB 2020	117	13557	333	14764	0.38	[0.31; 0.47]	2.3%
Self 2013	111	6590	321	7389	0.39	[0.31; 0.48]	2.3%
Sanchez 2018	47	841	22	158	0.40	[0.25; 0.65]	2.1%
Hall 2013	35	2153	212	5402	0.41	[0.29; 0.59]	2.2%
Thomas 2011	29	705	90	981	0.45	[0.30; 0.67]	2.1%
Krajčinović 2015	8	105	22	134	0.46	[0.22; 1.00]	1.7%
Shaheen 2020	380	9756	709	8868	0.49	[0.43; 0.55]	2.4%
Harding 2013	64	6336	101	5544	0.55	[0.41; 0.76]	2.3%
Self1 2014	142	5238	165	3417	0.56	[0.45; 0.70]	2.3%
Kim 2011	34	5265	60	5255	0.57	[0.37; 0.86]	2.1%
Lin 2012	31	1548	328	9645	0.59	[0.41; 0.85]	2.2%
Bae 2019	222	83534	354	78673	0.59	[0.50; 0.70]	2.4%
Al-Hamad 2016	231	4138	389	4521	0.65	[0.55; 0.76]	2.4%
Marini 2013	840	60000	1260	60000	0.67	[0.61; 0.73]	2.4%
Marlowe 2010	72	4274	122	4942	0.68	[0.51; 0.91]	2.3%
Syed 2020	239	14046	326	13350	0.70	[0.59; 0.82]	2.4%
Santos 2018	2	234	4	329	0.70	[0.13; 3.81]	0.8%
Tangsathapompong 2014	14	615	21	654	0.71	[0.36; 1.38]	1.8%
HalsteadC 2020	172	12507	215	11100	0.71	[0.58; 0.87]	2.3%
Yodoshi 2019	38	2407	65	2958	0.72	[0.48; 1.07]	2.2%
Roth 2010	278	12459	1006	38805	0.86	[0.75; 0.98]	2.4%
Murillo 2011	124	2531	114	2280	0.98	[0.76; 1.26]	2.3%
McLaughlin 2013	11	284	16	693	1.68	[0.79; 3.57]	1.7%
Martínez 2017	10	539	5	563	2.09	[0.72; 6.07]	1.3%
**Random effects model**		284241		366682	0.47	[0.39; 0.57]	70.7%
Heterogeneity: $I^2 = 89\%$, $\tau^2 = 0.2610$, $p < 0.01$							
**overall_ROB = High**							
Frota 2021	2	200	20	200	0.10	[0.02; 0.42]	0.9%
Rupp 2017	2	904	16	904	0.12	[0.03; 0.54]	0.9%
Ge 2011	5	3177	77	6144	0.13	[0.05; 0.31]	1.5%
Kai 2020	5	450	29	460	0.18	[0.07; 0.45]	1.5%
Levin 2013	9	172	27	141	0.27	[0.13; 0.56]	1.7%
Zimmerman 2019	7	325	17	312	0.40	[0.17; 0.94]	1.5%
Allen 2021	10	1110	39	1944	0.45	[0.23; 0.90]	1.8%
El Feghaly 2018	578	37500	1603	56250	0.54	[0.49; 0.59]	2.4%
Patton 2010	58	2672	41	1061	0.56	[0.38; 0.83]	2.2%
Ramirez 2015	40	308	80	348	0.56	[0.40; 0.80]	2.2%
Ryan 2017	21	1152	130	4197	0.59	[0.37; 0.93]	2.1%
Park 2015	922	83861	1212	88476	0.80	[0.74; 0.87]	2.4%
Story-Roller 2016	123	3130	115	2965	1.01	[0.79; 1.30]	2.3%
Tarai 2012	83	2553	47	4350	3.01	[2.11; 4.29]	2.2%
**Random effects model**		137514		167752	0.46	[0.29; 0.74]	25.7%
Heterogeneity: $I^2 = 92\%$, $\tau^2 = 0.6846$, $p < 0.01$							
**overall_ROB = Moderate**							
Nuntnarumit 2013	0	172	5	172	0.02	[0.00; 10.21]	0.1%

Risk Ratio

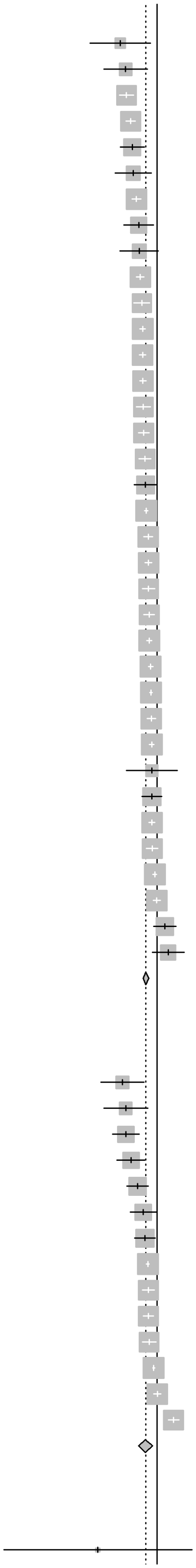

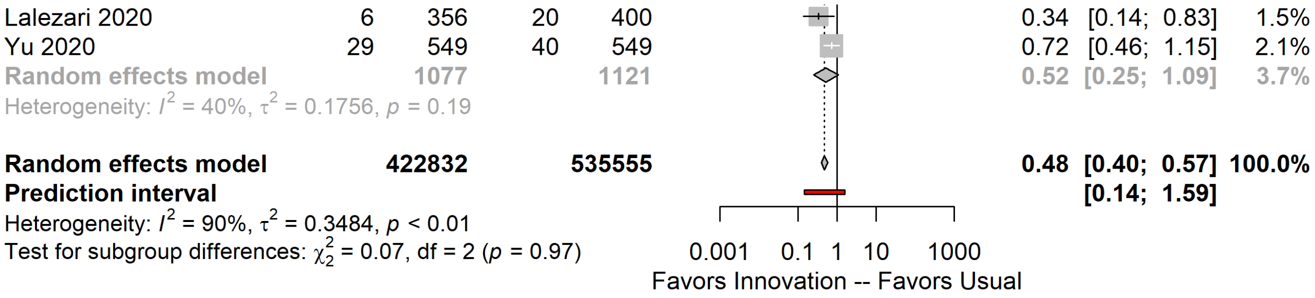

Improvement Focus and BCC Rate

While nearly all studies identified explicitly used multicomponent interventions, they differed in the primary focus of the intervention.

```
BCC_all_focus<-update.meta(BCC_all_studies.bin1a,
                           byvar= Focus,
                           comb.random = TRUE,
                           comb.fixed = FALSE,
                           bysort=TRUE)
BCC_all_focus
```

```
## Number of studies: k = 53
## Number of observations: o = 958387
## Number of events: e = 18545.67
##
##                          RR           95%-CI     z  p-value
## Random effects model 0.4784 [0.3997; 0.5727] -8.04 < 0.0001
## Prediction interval         [0.1442; 1.5872]
##
## Quantifying heterogeneity:
##  tau^2 = 0.3484 [0.2094; 0.6188]; tau = 0.5902 [0.4576; 0.7867]
##  I^2 = 89.8% [87.4%; 91.7%]; H = 3.13 [2.82; 3.47]
##
## Test of heterogeneity:
##       Q d.f.  p-value
##  508.34   52 < 0.0001
##
## Results for subgroups (random effects model):
##                              k     RR           95%-CI  tau^2    tau      Q
## Focus = Combined             9 0.4056 [0.2729; 0.6028] 0.3236 0.5688  79.46
## Focus = Profession focused   2 0.5916 [0.5008; 0.6989]      0      0   0.04
## Focus = Device focused       8 0.3757 [0.2237; 0.6310] 0.4288 0.6548  44.18
## Focus = Education focused   14 0.5251 [0.4084; 0.6752] 0.1939 0.4404 155.87
## Focus = Procedure focused    9 0.4849 [0.2710; 0.8674] 0.6920 0.8319 111.83
## Focus = Solution focused    11 0.5348 [0.3170; 0.9022] 0.5105 0.7145  28.52
##                              I^2
## Focus = Combined           89.9%
## Focus = Profession focused  0.0%
## Focus = Device focused     84.2%
## Focus = Education focused  91.7%
## Focus = Procedure focused  92.8%
## Focus = Solution focused   64.9%
##
## Test for subgroup differences (random effects model):
##                   Q d.f. p-value
## Between groups 5.19    5  0.3929
##
## Details on meta-analytical method:
## - Inverse variance method
## - Paule-Mandel estimator for tau^2
## - Q-Profile method for confidence interval of tau^2 and tau
## - Prediction interval based on t-distribution (df = 51)
## - Continuity correction of 0.1 in studies with zero cell frequencies
```

BCC Improvement by Year

```
m.year.reg <- metareg(BCC_All1a.bin, ~year)
m.year.reg
```

```
##
## Mixed-Effects Model (k = 53; tau^2 estimator: PM)
##
## tau^2 (estimated amount of residual heterogeneity):     0.3103 (SE = 0.0801)
## tau (square root of estimated tau^2 value):             0.5571
## I^2 (residual heterogeneity / unaccounted variability): 94.97%
## H^2 (unaccounted variability / sampling variability):   19.88
## R^2 (amount of heterogeneity accounted for):            10.92%
##
## Test for Residual Heterogeneity:
## QE(df = 51) = 431.6829, p-val < .0001
##
## Test of Moderators (coefficient 2):
## QM(df = 1) = 5.4340, p-val = 0.0197
##
## Model Results:
##
##          estimate       se     zval    pval    ci.lb     ci.ub
## intrcpt  117.2481  50.6115   2.3166  0.0205  18.0513  216.4449  *
## year      -0.0585   0.0251  -2.3311  0.0197  -0.1077   -0.0093  *
##
## ---
## Signif. codes:  0 '***' 0.001 '**' 0.01 '*' 0.05 '.' 0.1 ' ' 1
```

```
year.plot <- regplot(m.year.reg,
        mod="year",
        pred = TRUE,
        ci = TRUE,
        pi= TRUE,
        xlab="Publication Year",
        xlim=c(2008,2024),
        ylim=c(0,3.5),
        transf=exp,
        refline=1,
        legend=TRUE,
        label="piout",
        labsize=0.8,
        lcol = c("blue", "green", "red", "black"))
```

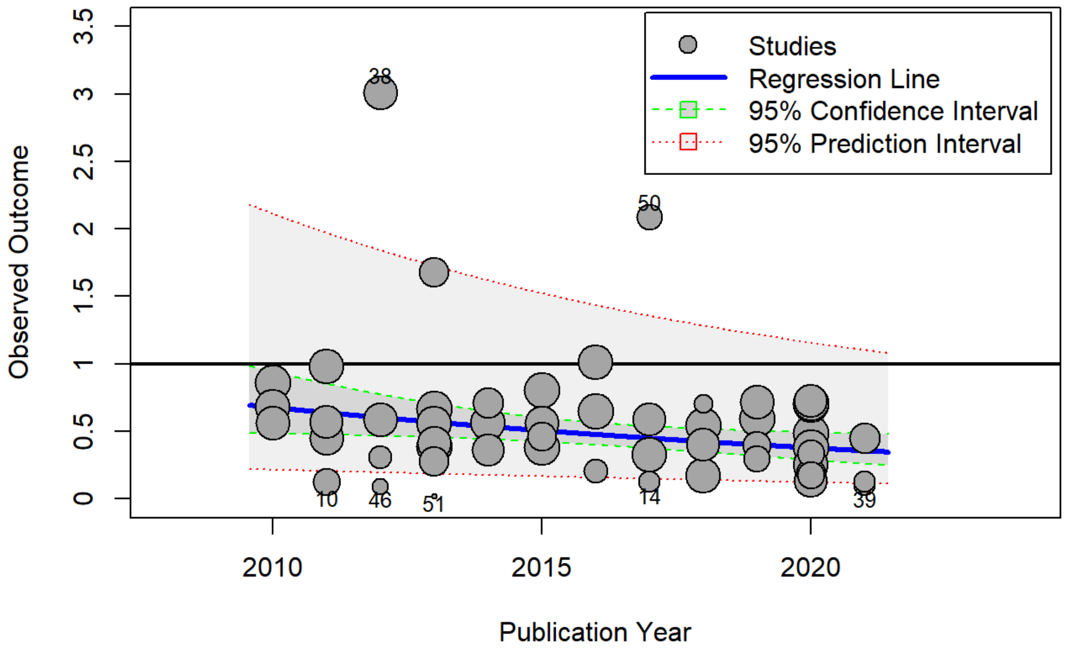

Effect by Study Design

```
BCC_all_studies.bin1a <- metabin(Ee,
                                 Ne,
                                 Ec,
                                 Nc,
                                 data = BCC_Outcomes1a,
                                 studlab = paste(Author, year),
                                 comb.fixed = FALSE,
                                 comb.random = TRUE,
                                 method.tau = "PM",
                                 hakn = FALSE,
                                 prediction = TRUE,
                                 incr = 0.1,
                                 sm = "RR")

BCC_all_studies.bin1a
```

```
## Number of studies: k = 53
## Number of observations: o = 958387
## Number of events: e = 18545.67
##
##                          RR           95%-CI     z  p-value
## Random effects model 0.4784 [0.3997; 0.5727] -8.04 < 0.0001
## Prediction interval         [0.1442; 1.5872]
##
## Quantifying heterogeneity:
##  tau^2 = 0.3484 [0.2094; 0.6188]; tau = 0.5902 [0.4576; 0.7867]
##  I^2 = 89.8% [87.4%; 91.7%]; H = 3.13 [2.82; 3.47]
##
## Test of heterogeneity:
##       Q d.f.  p-value
##  508.34   52 < 0.0001
##
## Details on meta-analytical method:
## - Inverse variance method
## - Paule-Mandel estimator for tau^2
## - Q-Profile method for confidence interval of tau^2 and tau
## - Prediction interval based on t-distribution (df = 51)
## - Continuity correction of 0.1 in studies with zero cell frequencies
```

```
BCC_all_design<-update.meta(BCC_all_studies.bin1a,
                         byvar=Design,
                         comb.random = TRUE,
                         comb.fixed = FALSE,
                         bysort=TRUE)
BCC_all_design
```

```
## Number of studies: k = 53
## Number of observations: o = 958387
## Number of events: e = 18545.67
##
##                           RR           95%-CI     z  p-value
## Random effects model 0.4784 [0.3997; 0.5727] -8.04 < 0.0001
## Prediction interval         [0.1442; 1.5872]
##
## Quantifying heterogeneity:
##  tau^2 = 0.3484 [0.2094; 0.6188]; tau = 0.5902 [0.4576; 0.7867]
##  I^2 = 89.8% [87.4%; 91.7%]; H = 3.13 [2.82; 3.47]
##
## Test of heterogeneity:
##       Q d.f.  p-value
##  508.34   52 < 0.0001
##
## Results for subgroups (random effects model):
##                            k     RR           95%-CI  tau^2    tau      Q
## Design = Before/After      34 0.4538 [0.3843; 0.5358] 0.1913 0.4373 302.37
## Design = Controlled Study  13 0.4457 [0.2793; 0.7113] 0.5111 0.7149  48.49
## Design = Cohort             6 0.7471 [0.3058; 1.8251] 1.1032 1.0504  70.20
##                            I^2
## Design = Before/After     89.1%
## Design = Controlled Study 75.3%
## Design = Cohort           92.9%
##
## Test for subgroup differences (random effects model):
##                   Q d.f. p-value
## Between groups 1.18    2  0.5556
##
## Details on meta-analytical method:
## - Inverse variance method
## - Paule-Mandel estimator for tau^2
## - Q-Profile method for confidence interval of tau^2 and tau
## - Prediction interval based on t-distribution (df = 51)
## - Continuity correction of 0.1 in studies with zero cell frequencies
```

Analysis for GRADE

Probably the most important hypothesis deals with the impact of including any discrete intervention as part of a larger process improvement effort (which would include such things as generating enthusiasm for the change, proper education and training, etc.). So, we can generate a GRADE based on Hypothesis 2 and focus on the findings of the different study design within those that implement the change as part of a larger QMQA effort.

To do this, we will first subset the dataset to utilize only those studies with QMQA.

```
QMQA <- dplyr::filter(BCC_Outcomes1a, `QMQA` %in% c("Yes"))
```

Next, we will further subset this QMQA dataset by design type: Before/After, Controlled Study, and Cohort.

```
QMQA_BA <- dplyr::filter(QMQA, `Design` %in% c("Before/After"))
QMQA_Cont <- dplyr::filter(QMQA, `Design` %in% c("Controlled Study"))
QMQA_Cohort <- dplyr::filter(QMQA, `Design` %in% c("Cohort"))
```

Finally, we will carry out a meta-analysis within each of these subsets.

```
QMQA_BA.bin <- metabin(Ee,
                        Ne,
                        Ec,
                        Nc,
                        data = QMQA_BA, #this is my data set
                        studlab = paste(Author, year),
                        comb.fixed = FALSE,
                        comb.random = TRUE, #here I'm doing a random effects model
                        method.tau = "PM", #I'm going to use a PM first
                        hakn = FALSE,
                        prediction = TRUE,
                        incr = 0.1,
                        sm = "RR")

QMQA_BA.bin
```

```
## Number of studies: k = 28
## Number of observations: o = 532668
## Number of events: e = 13837.67
##
##                           RR           95%-CI     z  p-value
## Random effects model 0.4331 [0.3598; 0.5214] -8.84 < 0.0001
## Prediction interval         [0.1702; 1.1020]
##
## Quantifying heterogeneity:
##  tau^2 = 0.1974 [0.1054; 0.4281]; tau = 0.4443 [0.3246; 0.6543]
##  I^2 = 90.5% [87.5%; 92.8%]; H = 3.25 [2.82; 3.73]
##
## Test of heterogeneity:
##       Q d.f.  p-value
##  284.41   27 < 0.0001
##
## Details on meta-analytical method:
## - Inverse variance method
## - Paule-Mandel estimator for tau^2
## - Q-Profile method for confidence interval of tau^2 and tau
## - Prediction interval based on t-distribution (df = 26)
```

```
QMQA_Cont.bin <- metabin(Ee,
                        Ne,
                        Ec,
                        Nc,
                        data = QMQA_Cont, #this is my data set
                        studlab = paste(Author, year),
                        comb.fixed = FALSE,
                        comb.random = TRUE, #here I'm doing a random effects model
                        method.tau = "PM", #I'm going to use a PM first
                        hakn = FALSE,
                        prediction = TRUE,
                        incr = 0.1,
                        sm = "RR")

QMQA_Cont.bin
```

```
## Number of studies: k = 6
## Number of observations: o = 13685
## Number of events: e = 525
##
##                           RR           95%-CI     z p-value
## Random effects model 0.5160 [0.3222; 0.8261] -2.76  0.0059
## Prediction interval         [0.1157; 2.3015]
##
## Quantifying heterogeneity:
##  tau^2 = 0.2324 [0.0261; 2.6395]; tau = 0.4820 [0.1616; 1.6246]
##  I^2 = 75.7% [45.4%; 89.2%]; H = 2.03 [1.35; 3.05]
##
## Test of heterogeneity:
##      Q d.f. p-value
##  20.61    5  0.0010
##
## Details on meta-analytical method:
## - Inverse variance method
## - Paule-Mandel estimator for tau^2
## - Q-Profile method for confidence interval of tau^2 and tau
## - Prediction interval based on t-distribution (df = 4)
```

```
QMQA_Cohort.bin <- metabin(Ee,
                        Ne,
                        Ec,
                        Nc,
                        data = QMQA_Cohort, #this is my data set
                        studlab = paste(Author, year),
                        comb.fixed = FALSE,
                        comb.random = TRUE, #here I'm doing a random effects model
                        method.tau = "PM", #I'm going to use a PM first
                        hakn = FALSE,
                        prediction = TRUE,
                        incr = 0.1,
                        sm = "RR")

QMQA_Cohort.bin
```

```
## Number of observations: o = 172337
## Number of events: e = 2134
##
##                RR           95%-CI     z  p-value
## Park 2015 0.8026 [0.7371; 0.8739] -5.06 < 0.0001
```

Discrete Interventions

In addition to the hypothesis driven analyses, we also want to break down the studies by focus: discrete interventions. The following interventions are examined:

- Use of Chlorhexidine
- Diversion Device
- Education/Training
- Peripheral v Catheter
- Sterile technique
- Phlebotomist

Chlorhexidine v Alternative

We will subsample out chlorhexidine focused studies and carry out a series of analyses.

```
# Focus on just the chlorhexidine group

chlorhexidine_BCR <- dplyr::filter(BCC_Outcomes1a,`Interv_cat` %in% c("chlorhexidine"))

# Perform meta-analysis on chlorhexidine for Risk Ratio

BCC_chlorhexidine.bin <- metabin(Ee,
                           Ne,
                           Ec,
                           Nc,
                           data = chlorhexidine_BCR,
                           studlab = paste(Author, year),
                           comb.fixed = FALSE,
                           comb.random = TRUE,
                           method.tau = "PM",
                           hakn = FALSE,
                           prediction = TRUE,
                           incr = 0.1,
                           sm = "RR")

BCC_chlorhexidine.bin
```

```
## Number of studies: k = 10
## Number of observations: o = 35744
## Number of events: e = 793
##
##                          RR           95%-CI     z p-value
## Random effects model 0.4355 [0.2328; 0.8144] -2.60  0.0092
## Prediction interval         [0.0521; 3.6376]
##
## Quantifying heterogeneity:
##  tau^2 = 0.7453 [0.2097; 3.7268]; tau = 0.8633 [0.4579; 1.9305]
##  I^2 = 80.3% [64.7%; 89.1%]; H = 2.26 [1.68; 3.02]
##
## Test of heterogeneity:
##      Q d.f.  p-value
##  45.78    9 < 0.0001
##
## Details on meta-analytical method:
## - Inverse variance method
## - Paule-Mandel estimator for tau^2
## - Q-Profile method for confidence interval of tau^2 and tau
## - Prediction interval based on t-distribution (df = 8)
## - Continuity correction of 0.1 in studies with zero cell frequencies
```

We need to make sure that the Yodoshi was dropped from this analysis, so we'll look at a summary of the results.

```
summary(BCC_chlorhexidine.bin)
```

```
##                           RR            95%-CI %W(random)
## Ge 2011               0.1256 [0.0509;  0.3100]       10.7
## Kai 2020              0.1762 [0.0688;  0.4512]       10.5
## O'Connor 2016         0.2046 [0.0605;  0.6916]        9.0
## Ryan 2017             0.5885 [0.3729;  0.9288]       12.8
## Story-Roller 2016     1.0132 [0.7897;  1.2999]       13.4
## Tangsathapompong 2014 0.7089 [0.3638;  1.3816]       11.8
## Maeda 2021            0.1239 [0.0287;  0.5345]        7.8
## Marlowe 2010          0.6824 [0.5114;  0.9105]       13.3
## Martínez 2017         2.0891 [0.7187;  6.0721]        9.8
## Nuntnarumit 2013      0.0196 [0.0000; 10.2065]        0.9
##
## Number of studies: k = 10
## Number of observations: o = 35744
## Number of events: e = 793
##
##                          RR           95%-CI     z p-value
## Random effects model 0.4355 [0.2328; 0.8144] -2.60  0.0092
## Prediction interval         [0.0521; 3.6376]
##
## Quantifying heterogeneity:
##  tau^2 = 0.7453 [0.2097; 3.7268]; tau = 0.8633 [0.4579; 1.9305]
##  I^2 = 80.3% [64.7%; 89.1%]; H = 2.26 [1.68; 3.02]
##
## Test of heterogeneity:
##      Q d.f.  p-value
##  45.78    9 < 0.0001
##
## Details on meta-analytical method:
## - Inverse variance method
## - Paule-Mandel estimator for tau^2
## - Q-Profile method for confidence interval of tau^2 and tau
## - Prediction interval based on t-distribution (df = 8)
## - Continuity correction of 0.1 in studies with zero cell frequencies
```

No Yodoshi. So, we'll create a forestplot.

```
png(file = "C:/Users/Scott/OneDrive - Rutgers University/ASM/ASM BCC Project/3.0 Data/3.4 BCC Meta-Analyses/3.3.3 Plots/chlorhex_forest.png", width = 2800, height = 1600, res = 300)

forest(BCC_chlorhexidine.bin, sortvar = TE, xlab = "Favors Innovation -- Favors Usual")
dev.off()
```

```
## png
##   2
```

```
knitr::include_graphics("C:/Users/Scott/OneDrive - Rutgers University/ASM/ASM BCC Project/3.0 Data/3.4 BCC Meta-Analyses/3.3.3 Plots/chlorhex_forest.png")
```

Study	Experimental Events	Experimental Total	Control Events	Control Total	RR	95%-CI	Weight
Nuntnarumit 2013	0	172	5	172	0.02	[0.00; 10.21]	0.9%
Maeda 2021	2	711	17	749	0.12	[0.03; 0.53]	7.8%
Ge 2011	5	3177	77	6144	0.13	[0.05; 0.31]	10.7%
Kai 2020	5	450	29	460	0.18	[0.07; 0.45]	10.5%
O'Connor 2016	3	314	17	364	0.20	[0.06; 0.69]	9.0%
Ryan 2017	21	1152	130	4197	0.59	[0.37; 0.93]	12.8%
Marlowe 2010	72	4274	122	4942	0.68	[0.51; 0.91]	13.3%
Tangsathapompong 2014	14	615	21	654	0.71	[0.36; 1.38]	11.8%
Story-Roller 2016	123	3130	115	2965	1.01	[0.79; 1.30]	13.4%
Martínez 2017	10	539	5	563	2.09	[0.72; 6.07]	9.8%
**Random effects model**		**14534**		**21210**	**0.44**	**[0.23; 0.81]**	**100.0%**
**Prediction interval**						**[0.05; 3.64]**	

Heterogeneity: $I^2 = 80\%$, $\tau^2 = 0.7453$, $p < 0.01$

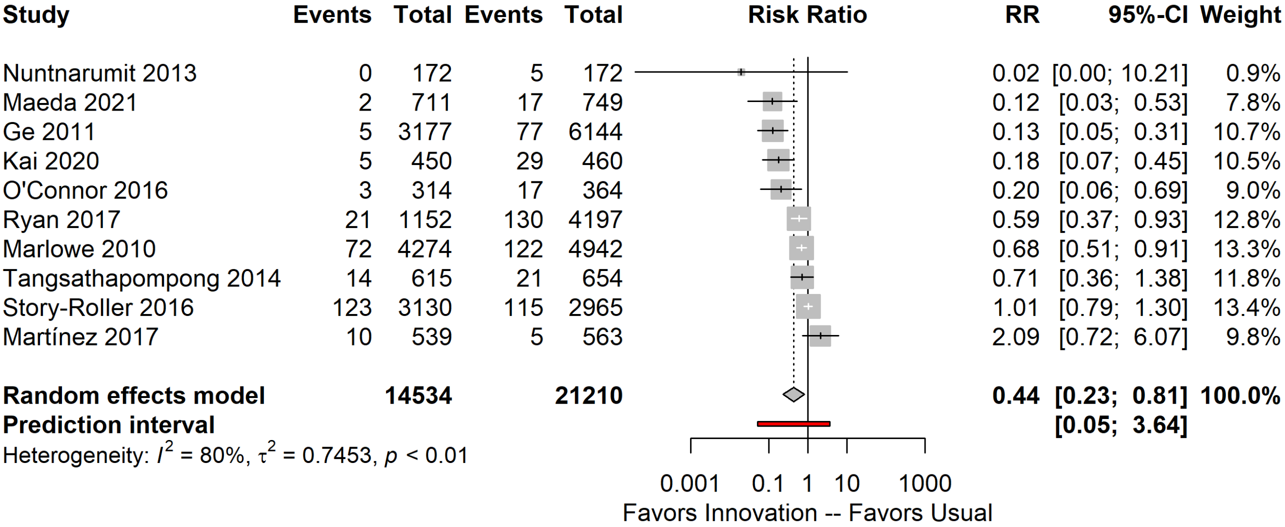

Chlorhexidine: Variation Mix

Now we will subgroup by chlorhexidine alone versus chlorhexidine with alcohol

```
chlor_mix<-update.meta(BCC_chlorhexidine.bin,
                                        byvar=variation_mix,
                                        comb.random = TRUE,
                                        comb.fixed = FALSE,
                                        bysort=TRUE)
chlor_mix
```

```
## Number of studies: k = 10
## Number of observations: o = 35744
## Number of events: e = 793
##
##                           RR           95%-CI     z p-value
## Random effects model 0.4355 [0.2328; 0.8144] -2.60  0.0092
## Prediction interval         [0.0521; 3.6376]
##
## Quantifying heterogeneity:
##  tau^2 = 0.7453 [0.2097; 3.7268]; tau = 0.8633 [0.4579; 1.9305]
##  I^2 = 80.3% [64.7%; 89.1%]; H = 2.26 [1.68; 3.02]
##
## Test of heterogeneity:
##      Q d.f.  p-value
##  45.78    9 < 0.0001
##
## Results for subgroups (random effects model):
##                                  k     RR           95%-CI  tau^2    tau     Q
## variation_mix = CHG Alone         5 0.3341 [0.1236; 0.9034] 0.8814 0.9388 28.79
## variation_mix = CHG and Alcohol   5 0.5313 [0.2265; 1.2458] 0.7533 0.8679 15.59
##                                  I^2
## variation_mix = CHG Alone       86.1%
## variation_mix = CHG and Alcohol 74.3%
##
## Test for subgroup differences (random effects model):
##                  Q d.f. p-value
## Between groups 0.48    1  0.4878
##
## Details on meta-analytical method:
## - Inverse variance method
## - Paule-Mandel estimator for tau^2
## - Q-Profile method for confidence interval of tau^2 and tau
## - Prediction interval based on t-distribution (df = 8)
## - Continuity correction of 0.1 in studies with zero cell frequencies
```

```
png(file = "C:/Users/Scott/OneDrive - Rutgers University/ASM/ASM BCC Project/3.0 Data/3.4 BCC Meta-An
alyses/3.3.3 Plots/chlorhex_mix_forest.png", width = 2800, height = 2000, res = 300)

forest(chlor_mix, sortvar = TE, xlab = "Favors Innovation -- Favors Usual")
dev.off()
```

```
## png
##   2
```

```
knitr::include_graphics("C:/Users/Scott/OneDrive - Rutgers University/ASM/ASM BCC Project/3.0 Data/3.
4 BCC Meta-Analyses/3.3.3 Plots/chlorhex_mix_forest.png")
```

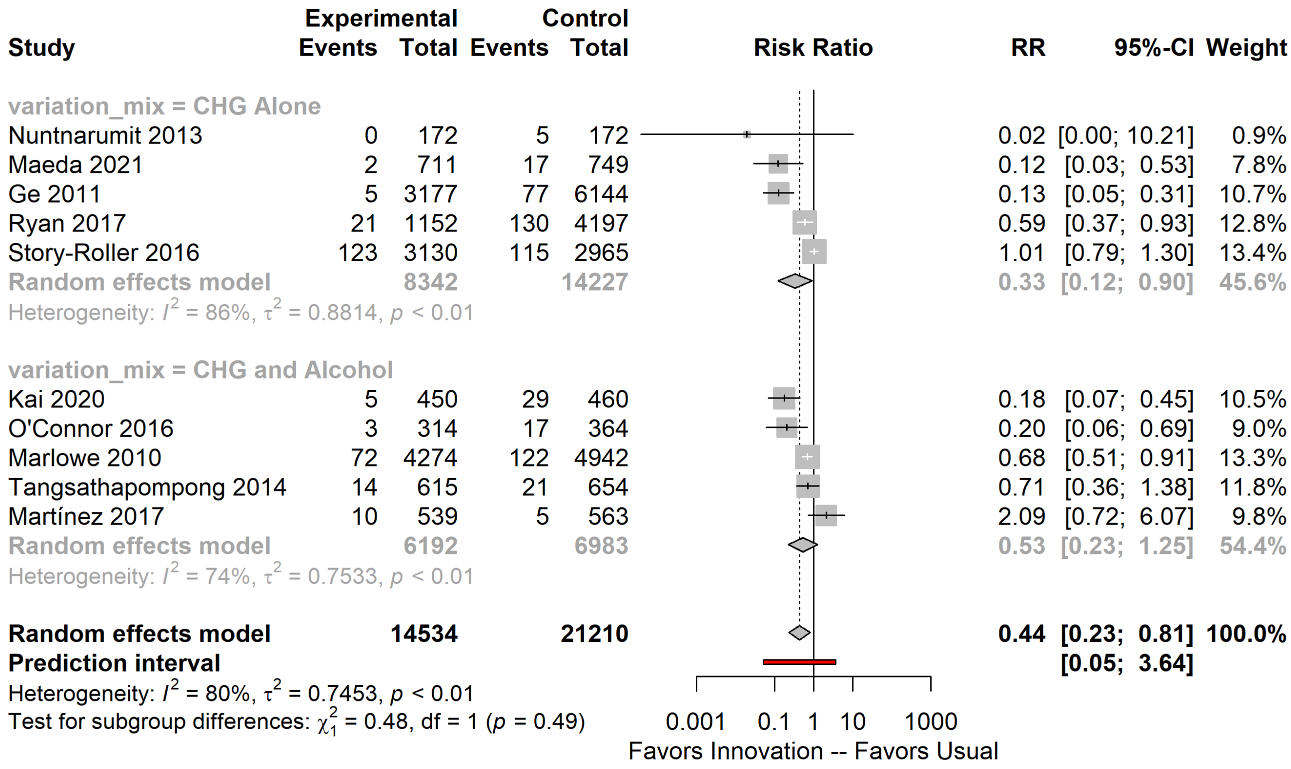

Chlorhexidine: Adults v Pedes

Now, subgroup by age group.

```
chlor_pop<-update.meta(BCC_chlorhexidine.bin,
                                         byvar=Population,
                                         comb.random = TRUE,
                                         comb.fixed = FALSE,
                                         bysort=TRUE)
chlor_pop
```

```
## Number of studies: k = 10
## Number of observations: o = 35744
## Number of events: e = 793
##
##                           RR           95%-CI     z p-value
## Random effects model 0.4355 [0.2328; 0.8144] -2.60  0.0092
## Prediction interval         [0.0521; 3.6376]
##
## Quantifying heterogeneity:
##  tau^2 = 0.7453 [0.2097; 3.7268]; tau = 0.8633 [0.4579; 1.9305]
##  I^2 = 80.3% [64.7%; 89.1%]; H = 2.26 [1.68; 3.02]
##
## Test of heterogeneity:
##      Q d.f.  p-value
##  45.78    9 < 0.0001
##
## Results for subgroups (random effects model):
##                       k     RR           95%-CI  tau^2    tau     Q   I^2
## Population = Adult   5 0.4947 [0.1807; 1.3546] 1.1678 1.0806 34.28 88.3%
## Population = Pedes   5 0.3951 [0.1811; 0.8622] 0.4366 0.6608  9.59 58.3%
##
## Test for subgroup differences (random effects model):
##                    Q d.f. p-value
## Between groups 0.12    1  0.7295
##
## Details on meta-analytical method:
## - Inverse variance method
## - Paule-Mandel estimator for tau^2
## - Q-Profile method for confidence interval of tau^2 and tau
## - Prediction interval based on t-distribution (df = 8)
## - Continuity correction of 0.1 in studies with zero cell frequencies
```

```
png(file = "C:/Users/Scott/OneDrive - Rutgers University/ASM/ASM BCC Project/3.0 Data/3.4 BCC Meta-Analyses/3.3.3 Plots/chlorhex_pop_forest.png", width = 2800, height = 2000, res = 300)

forest(chlor_pop, sortvar = TE, xlab = "Favors Innovation -- Favors Usual")
dev.off()
```

```
## png
##   2
```

```
knitr::include_graphics("C:/Users/Scott/OneDrive - Rutgers University/ASM/ASM BCC Project/3.0 Data/3.4 BCC Meta-Analyses/3.3.3 Plots/chlorhex_pop_forest.png")
```

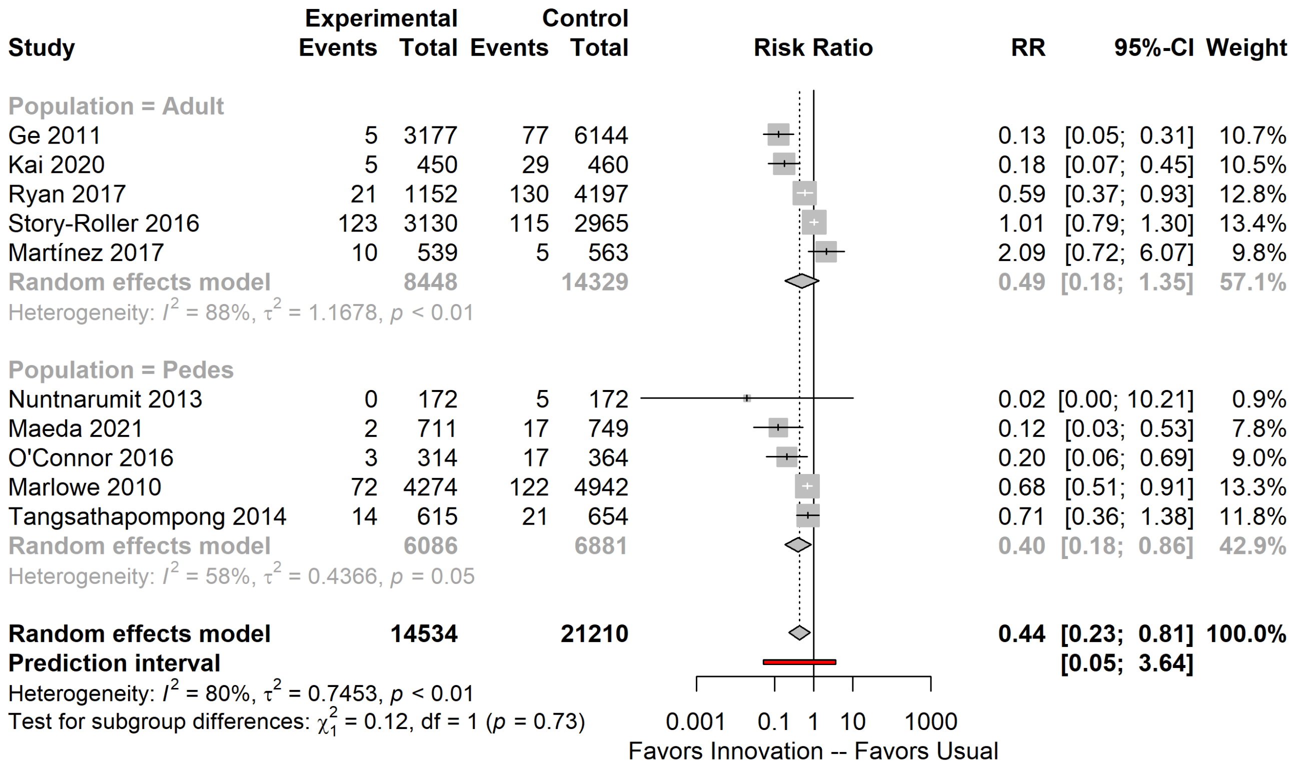

Diversion Devices

Use of diversion devices.

```
# Focus on just the Diversion Device group

Diversion_BCR <- dplyr::filter(BCC_Outcomes1a,`Interv_cat` %in% c("Diversion device"))

# Perform meta-analysis on Diversion Device for Risk Ratio

BCC_diversion.bin <- metabin(Ee,
                             Ne,
                             Ec,
                             Nc,
                             data = Diversion_BCR,
                             studlab = paste(Author, year),
                             comb.fixed = FALSE,
                             comb.random = TRUE,
                             method.tau = "PM",
                             hakn = FALSE,
                             prediction = TRUE,
                             incr = 0.1,
                             sm = "RR")

BCC_diversion.bin
```

```
## Number of studies: k = 6
## Number of observations: o = 76015
## Number of events: e = 2016
##
##                            RR           95%-CI     z p-value
## Random effects model 0.3605 [0.2149; 0.6046] -3.87  0.0001
## Prediction interval         [0.0665; 1.9530]
##
## Quantifying heterogeneity:
##  tau^2 = 0.3008 [0.0849; 2.4761]; tau = 0.5484 [0.2914; 1.5736]
##  I^2 = 92.0% [85.4%; 95.6%]; H = 3.54 [2.62; 4.79]
##
## Test of heterogeneity:
##      Q d.f.  p-value
##  62.70    5 < 0.0001
##
## Details on meta-analytical method:
## - Inverse variance method
## - Paule-Mandel estimator for tau^2
## - Q-Profile method for confidence interval of tau^2 and tau
## - Prediction interval based on t-distribution (df = 4)
```

```
png(file = "C:/Users/Scott/OneDrive - Rutgers University/ASM/ASM BCC Project/3.0 Data/3.4 BCC Meta-An
alyses/3.3.3 Plots/diversion_forest.png", width = 2800, height = 1200, res = 300)

forest(BCC_diversion.bin, sortvar = TE, xlab = "Favors Innovation -- Favors Usual")
dev.off()
```

```
## png
##   2
```

```
knitr::include_graphics("C:/Users/Scott/OneDrive - Rutgers University/ASM/ASM BCC Project/3.0 Data/3.
4 BCC Meta-Analyses/3.3.3 Plots/diversion_forest.png")
```

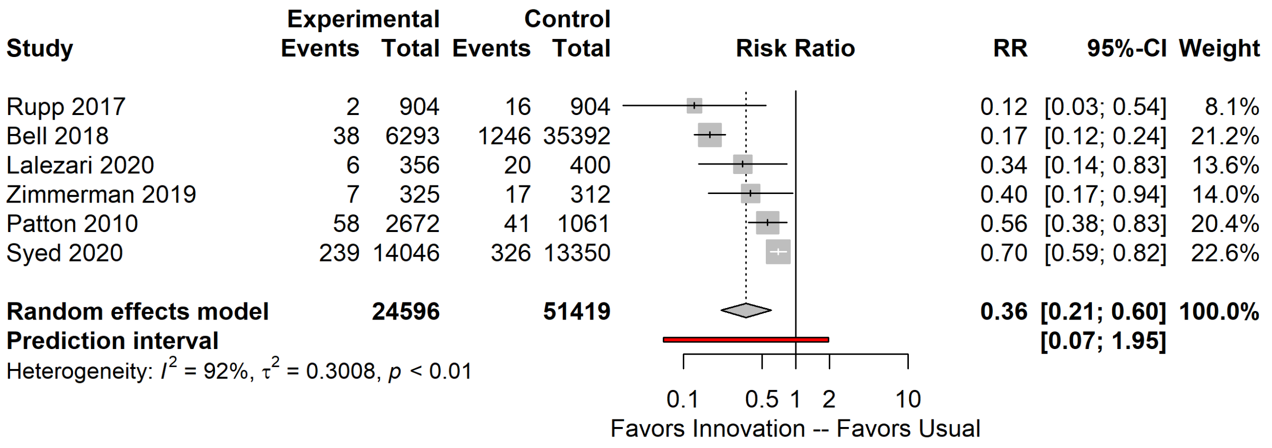

Education and Training

Now by education and training interventions (present v absent).

```
# Focus on just the Education/Training group

EDTrain_BCR <- dplyr::filter(BCC_Outcomes1a,`Interv_cat` %in% c("Education/Training"))

# Perform meta-analysis on Education/Training for Risk Ratio

BCC_edtrain.bin <- metabin(Ee,
                             Ne,
                             Ec,
                             Nc,
                             data = EDTrain_BCR, #the data focused on Education/Training
                             studlab = paste(Author, year),
                             comb.fixed = FALSE,
                             comb.random = TRUE, #here I'm doing a random effects model
                             method.tau = "PM", #I'm going to use a PM first
                             hakn = FALSE,
                             prediction = TRUE,
                             incr = 0.1,
                             sm = "RR")

BCC_edtrain.bin
```

```
## Number of studies: k = 16
## Number of observations: o = 481726
## Number of events: e = 9619.994
##
##                          RR           95%-CI     z  p-value
## Random effects model 0.4799 [0.3650; 0.6310] -5.26 < 0.0001
## Prediction interval         [0.1494; 1.5417]
##
## Quantifying heterogeneity:
##  tau^2 = 0.2766 [0.1317; 0.7315]; tau = 0.5259 [0.3630; 0.8553]
##  I^2 = 92.6% [89.6%; 94.8%]; H = 3.69 [3.10; 4.38]
##
## Test of heterogeneity:
##       Q d.f.  p-value
##  203.87   15 < 0.0001
##
## Details on meta-analytical method:
## - Inverse variance method
## - Paule-Mandel estimator for tau^2
## - Q-Profile method for confidence interval of tau^2 and tau
## - Prediction interval based on t-distribution (df = 14)
```

```
png(file = "C:/Users/Scott/OneDrive - Rutgers University/ASM/ASM BCC Project/3.0 Data/3.4 BCC Meta-An
alyses/3.3.3 Plots/edtrain_forest.png", width = 2800, height = 2000, res = 300)

forest(BCC_edtrain.bin, sortvar = TE, xlab = "Favors Innovation -- Favors Usual")
dev.off()
```

```
## png
##   2
```

```
knitr::include_graphics("C:/Users/Scott/OneDrive - Rutgers University/ASM/ASM BCC Project/3.0 Data/3.
4 BCC Meta-Analyses/3.3.3 Plots/edtrain_forest.png")
```

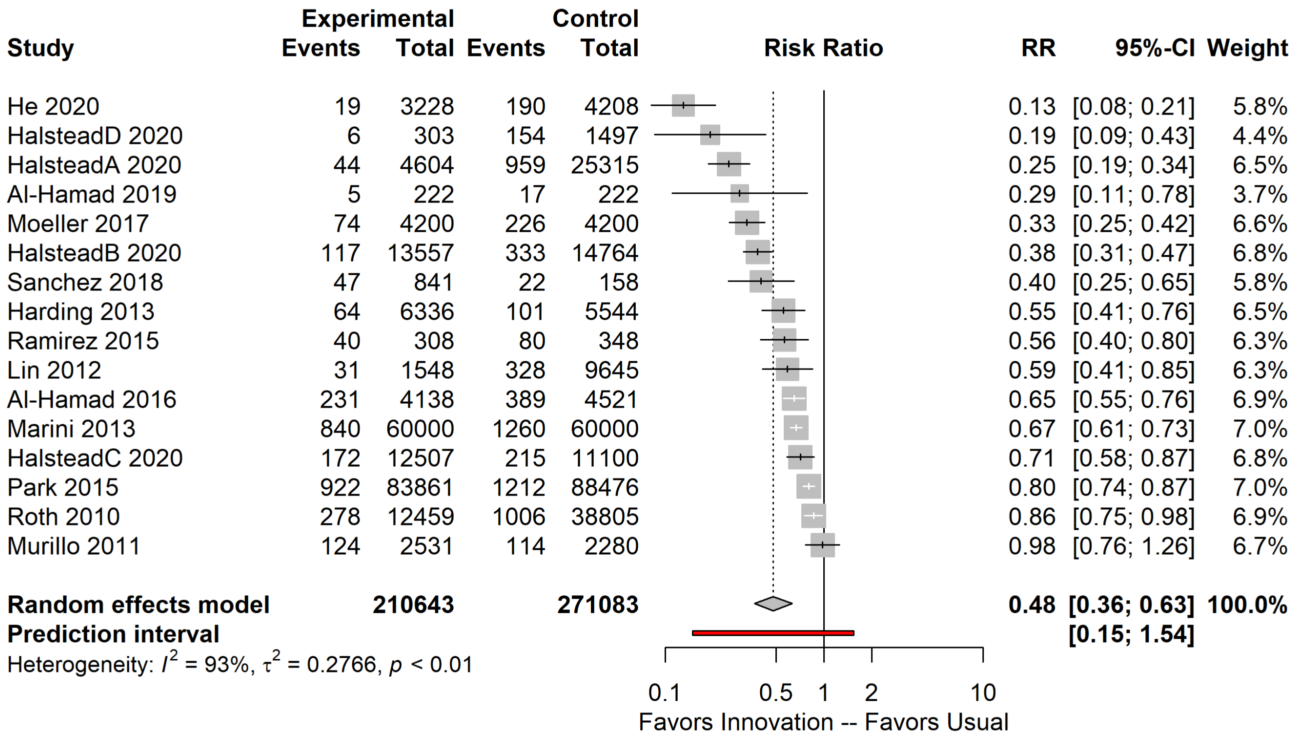

Phlebotomist

Now compare use of phlebotomists v not.

```
# Focus on just the Phlebotomy Team group

Phleb_BCR <- dplyr::filter(BCC_Outcomes1a,`Interv_cat` %in% c("Phlebotomy Team"))

# Perform meta-analysis on Phlebotomy Team for Risk Ratio

BCC_phleb.bin <- metabin(Ee,
                         Ne,
                         Ec,
                         Nc,
                         data = Phleb_BCR, #the data focused on Phlebotomy Team
                         studlab = paste(Author, year),
                         comb.fixed = FALSE,
                         comb.random = TRUE, #here I'm doing a random effects model
                         method.tau = "PM", #I'm going to use a PM first
                         hakn = FALSE,
                         prediction = TRUE,
                         incr = 0.1,
                         sm = "RR")

BCC_phleb.bin
```

```
## Number of studies: k = 2
## Number of observations: o = 162770
## Number of events: e = 582
##
##                          RR           95%-CI     z  p-value
## Random effects model 0.5916 [0.5008; 0.6989] -6.17 < 0.0001
## Prediction interval
##
## Quantifying heterogeneity:
##  tau^2 = 0; tau = 0; I^2 = 0.0%; H = 1.00
##
## Test of heterogeneity:
##     Q d.f. p-value
##  0.04    1  0.8406
##
## Details on meta-analytical method:
## - Inverse variance method
## - Paule-Mandel estimator for tau^2
```

```
png(file = "C:/Users/Scott/OneDrive - Rutgers University/ASM/ASM BCC Project/3.0 Data/3.4 BCC Meta-An
alyses/3.3.3 Plots/phleb_forest.png", width = 2800, height = 1200, res = 300)

forest(BCC_phleb.bin, sortvar = TE, xlab = "Favors Innovation -- Favors Usual")
dev.off()
```

```
## png
##   2
```

```
knitr::include_graphics("C:/Users/Scott/OneDrive - Rutgers University/ASM/ASM BCC Project/3.0 Data/3.
4 BCC Meta-Analyses/3.3.3 Plots/phleb_forest.png")
```

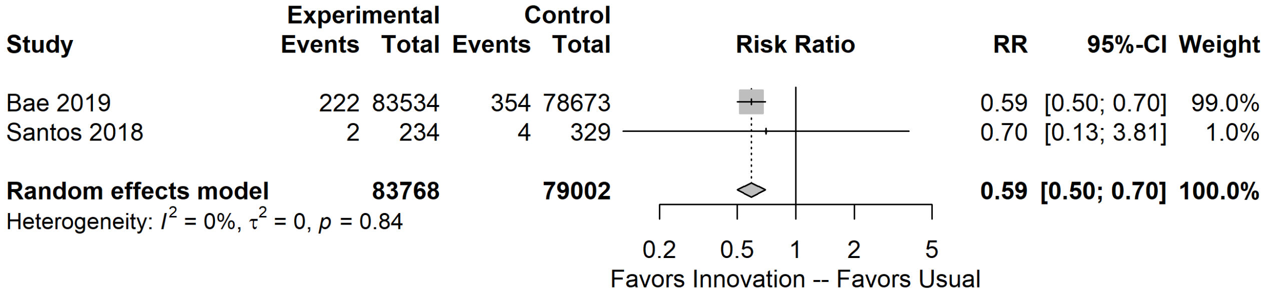

Sterile Technique

Effectiveness of using some sort of enhanced sterile technique

```
# Focus on just the Sterile technique group

Sterile_BCR <- dplyr::filter(BCC_Outcomes1a,`Interv_cat` %in% c("Sterile technique"))

# Perform meta-analysis on Sterile technique for Risk Ratio

BCC_sterile.bin <- metabin(Ee,
                       Ne,
                       Ec,
                       Nc,
                       data = Sterile_BCR, #the data focused on Sterile technique
                       studlab = paste(Author, year),
                       comb.fixed = FALSE,
                       comb.random = TRUE, #here I'm doing a random effects model
                       method.tau = "PM", #I'm going to use a PM first
                       hakn = FALSE,
                       prediction = TRUE,
                       incr = 0.1,
                       sm = "RR")

BCC_sterile.bin
```

```
## Number of studies: k = 7
## Number of observations: o = 45717
## Number of events: e = 1212
##
##                          RR           95%-CI     z  p-value
## Random effects model 0.4401 [0.3488; 0.5554] -6.91 < 0.0001
## Prediction interval         [0.2317; 0.8360]
##
## Quantifying heterogeneity:
##  tau^2 = 0.0482 [0.0000; 1.2986]; tau = 0.2196 [0.0000; 1.1396]
##  I^2 = 49.6% [0.0%; 78.6%]; H = 1.41 [1.00; 2.16]
##
## Test of heterogeneity:
##      Q d.f. p-value
##  11.90    6  0.0643
##
## Details on meta-analytical method:
## - Inverse variance method
## - Paule-Mandel estimator for tau^2
## - Q-Profile method for confidence interval of tau^2 and tau
## - Prediction interval based on t-distribution (df = 5)
```

```
png(file = "C:/Users/Scott/OneDrive - Rutgers University/ASM/ASM BCC Project/3.0 Data/3.4 BCC Meta-An
alyses/3.3.3 Plots/sterile_forest.png", width = 2800, height = 1400, res = 300)

forest(BCC_sterile.bin, sortvar = TE, xlab = "Favors Innovation -- Favors Usual")
dev.off()
```

```
## png
##   2
```

```
knitr::include_graphics("C:/Users/Scott/OneDrive - Rutgers University/ASM/ASM BCC Project/3.0 Data/3.
4 BCC Meta-Analyses/3.3.3 Plots/sterile_forest.png")
```

Study	Experimental Events	Experimental Total	Control Events	Control Total	RR	95%-CI	Weight
Frota 2021	2	200	20	200	0.10	[0.02; 0.42]	2.4%
Self2 2014	17	1860	63	2509	0.36	[0.21; 0.62]	11.6%
Self 2013	111	6590	321	7389	0.39	[0.31; 0.48]	23.5%
Hall 2013	35	2153	212	5402	0.41	[0.29; 0.59]	17.4%
Krajčinović 2015	8	105	22	134	0.46	[0.22; 1.00]	7.0%
Self1 2014	142	5238	165	3417	0.56	[0.45; 0.70]	23.2%
Kim 2011	34	5265	60	5255	0.57	[0.37; 0.86]	15.0%
**Random effects model**		**21411**		**24306**	**0.44**	**[0.35; 0.56]**	**100.0%**
**Prediction interval**						**[0.23; 0.84]**	

Heterogeneity: $I^2 = 50\%$, $\tau^2 = 0.0482$, $p = 0.06$

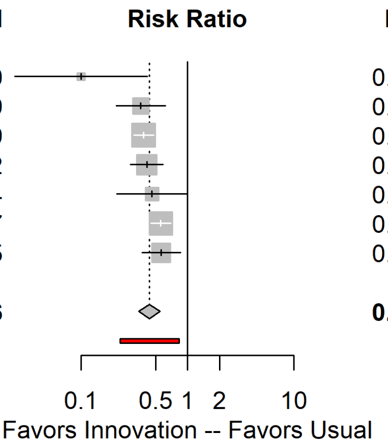

Supplement: BCC Meta-Analyses — Codes used for the main analyses, hypothesis tests, sources for heterogeneity, analysis for GRADE, interventions and hypothesis tests. [file cmr.00087-24-s0004.pdf]
